# Evolution of Mutational Robustness in the Yeast Genome: A Link to Essential Genes and Meiotic Recombination Hotspots

**DOI:** 10.1371/journal.pgen.1000533

**Published:** 2009-06-26

**Authors:** Philipp J. Keller, Michael Knop

**Affiliations:** European Molecular Biology Laboratory (EMBL), Cell Biology and Biophysics Unit, Heidelberg, Germany; Fred Hutchinson Cancer Research Center, United States of America

## Abstract

Deleterious mutations inevitably emerge in any evolutionary process and are speculated to decisively influence the structure of the genome. Meiosis, which is thought to play a major role in handling mutations on the population level, recombines chromosomes via non-randomly distributed hot spots for meiotic recombination. In many genomes, various types of genetic elements are distributed in patterns that are currently not well understood. In particular, important (essential) genes are arranged in clusters, which often cannot be explained by a functional relationship of the involved genes. Here we show by computer simulation that essential gene (EG) clustering provides a fitness benefit in handling deleterious mutations in sexual populations with variable levels of inbreeding and outbreeding. We find that recessive lethal mutations enforce a selective pressure towards clustered genome architectures. Our simulations correctly predict (i) the evolution of non-random distributions of meiotic crossovers, (ii) the genome-wide anti-correlation of meiotic crossovers and EG clustering, (iii) the evolution of EG enrichment in pericentromeric regions and (iv) the associated absence of meiotic crossovers (cold centromeres). Our results furthermore predict optimal crossover rates for yeast chromosomes, which match the experimentally determined rates. Using a *Saccharomyces cerevisiae* conditional mutator strain, we show that haploid lethal phenotypes result predominantly from mutation of single loci and generally do not impair mating, which leads to an accumulation of mutational load following meiosis and mating. We hypothesize that purging of deleterious mutations in essential genes constitutes an important factor driving meiotic crossover. Therefore, the increased robustness of populations to deleterious mutations, which arises from clustered genome architectures, may provide a significant selective force shaping crossover distribution. Our analysis reveals a new aspect of the evolution of genome architectures that complements insights about molecular constraints, such as the interference of pericentromeric crossovers with chromosome segregation.

## Author Summary

Sexual life cycles constitute a costly alternative to vegetative modes of reproduction. Two categories of hypotheses seek to explain why sexual life cycles exist: those investigating the selective advantages that have driven the evolution of individual parts of this life cycle and those rationalizing the advantages sexual life cycles may offer as a whole, e.g., in extant species. Sex and recombination can be understood as efficient ways to interact with mutations and their consequences. Mutations occur at random and are mostly either deleterious or neutral. A prominent hypothesis suggests that sex and recombination are advantageous since they enhance the purging of such deleterious mutations and create individuals with a lower than average deleterious load. Deleterious mutations should co-determine the parameters that govern recombination of genomes in meiosis. Using an evolutionary computer simulation of diploid, unicellular sexual populations, we show that recessive lethal mutations can drive the evolution of chromosome architectures, in which essential genes become genetically linked into clusters. Evolved architectures exhibit structural properties and fitness similar to digitized yeast chromosomes and provide mutational purging capabilities superior to those of randomly generated or unclustered architectures. Our study demonstrates the importance of sexual cycles in the context of lethal mutations.

## Introduction

Mating and meiosis are the masterpieces of an evolutionary invention thought to meet the challenges of changing environmental conditions that need to be solved by mutational inventions. Among the many hypotheses that govern the various benefits of mating and meiosis [1], two main hypotheses stand out: enhanced purging of deleterious mutations [2] and the combination of beneficial alleles into one genome [3]. It remains a matter of discussion, however, which of these advantages constitutes the main reason for the evolution of sexual recombination and, furthermore, its continuing prevalence in most eukaryotic life forms [4],[5].

Mutations take the form of DNA lesions that are caused by environmental factors, e.g. radiation, but they are also a natural byproduct of DNA replication. Genotypes that exhibit an elevated mutation rate are frequent in nature and can be induced in studies on experimental evolution. The complex interplay of factors that govern the adaptive significance of “mutator alleles” (i.e. alleles that cause higher mutation rates) has been studied in experimental and theoretical work in unicellular organisms [6]–[8] and during cancer progression [9],[10]. In asexual yeast populations, a selective advantage of mutator alleles has been demonstrated, serving as a prerequisite for expanding the spectrum of mutations typically not accessible in non-mutator genotypes [11]. In this study with yeast, the mutator advantage was found to be more prominent in diploid rather than haploid cells, which can be explained by the presumed dominance of beneficial mutations [11],[12] and by the recessive nature of most deleterious mutations in yeast [13]. Furthermore, the accumulation of recessive deleterious mutations in yeast may not significantly decrease the fitness of the genotype or population growth rates, as long as the diploid nuclear condition is maintained [14].

*S. cerevisiae* and many other yeast and fungal species seek to maintain the diploid state of the genome, if possible; after meiosis this usually happens by immediate mating of the gametes following germination. Mating occurs mostly between closely related spores, either among products of the same meiosis, or between spores from related cells according to population structure [15]. Outcrossing between unrelated strains [16] and even between closely related species of the *sensu stricto* yeast group does occur [17]; these events appear to be extremely rare, but they might be important for generating new persisting lineages. However, whether these rare events suffice to create a selective pressure towards maintaining a sexual cycle is doubtable.

Upon inbreeding, high rates of homozygotisation occur at various loci. This is reduced for loci linked to the *MAT* locus, since mating type heterozygosity is the prerequisite for a mating event. A *MAT*-linkage to centromeres is frequently observed in yeast and other fungi [18]. In *S. cerevisiae*, the genetic distance between the two loci is in the range of 18–30 cM [19], which is much smaller than expected from the physical length and caused by a region intervening these two loci that is cold for meiotic recombination [20]. In *Neurospora tetrasperma MAT* linkage to the centromere is enforced by crossover suppression in a long region of the chromosome, which correlates with an extensive unpaired region at pachytene [21]. Reasoning for such arrangements is provided by population genetic models that suggest a selective advantage arising from shielding of recurrent deleterious load via linkage to the *MAT* locus [22]. Population genetic modifier models that investigate alteration of the inbreeding frequency predict the evolution of high inbreeding rates, in particular for spores from the same tetrad (i.e. automixis), and the linkage of load loci to the *MAT* locus [23],[24]. These *MAT* linked chromosomal recombination abnormalities are believed to have initiated the evolution of sex chromosomes, which was then continued by expansion of the recombination suppressed region through the recruitment of other sex-related factors [25],[26].

Non-random distribution of meiotic recombination throughout the genomes into cold and hot regions has been reported for many species [27], but the molecular mechanisms as well as the selective forces that generate these patterns are still not fully understood.

Similarly, the distribution of genes along chromosomes appears to be non-random, and in many species a significant clustering of essential genes (“housekeeping genes”) has been reported [28],[29]. In budding yeast, a prominent genome-wide enrichment of essential genes has been observed in regions that are cold for meiotic recombination [30]. This finding is consistent with the observation of a slight enrichement of essential genes near the centromeres [31], which are known to be cold for meiotic recombination [32],[33]. Current models speculate about mechanisms of co-expression and reduction of gene expression noise as the driving force that shaped these patterns [29], [30], [34]–[36]. However, the selective advantage of such a scenario has not been demonstrated, and proof that single rearrangements associate with an advantage sufficient for their selection has not been provided.

An alternative mechanism that may pool essential genes into clusters with a reduced probability of disruption by frequent crossovers could be a selection based on their common denominator. This appears to be their essential nature only, as no functional correlation of essential genes within the same cluster has been observed [30].

Meiotic recombination is favorable for purging deleterious load from populations. We hypothesize that clustering of essential genes may further enhance purging efficiency of lethal load from sexual populations, since non-uniform distributions of essential genes and crossover sites change the global genetic linkage relationship of all essential genes (as compared to situations with uniform or random distributions). In the context of a scenario with more than one lethal mutation in the genome, this will influence the segregation of mutations during meiosis and subsequent mating.

**Figure 1:**
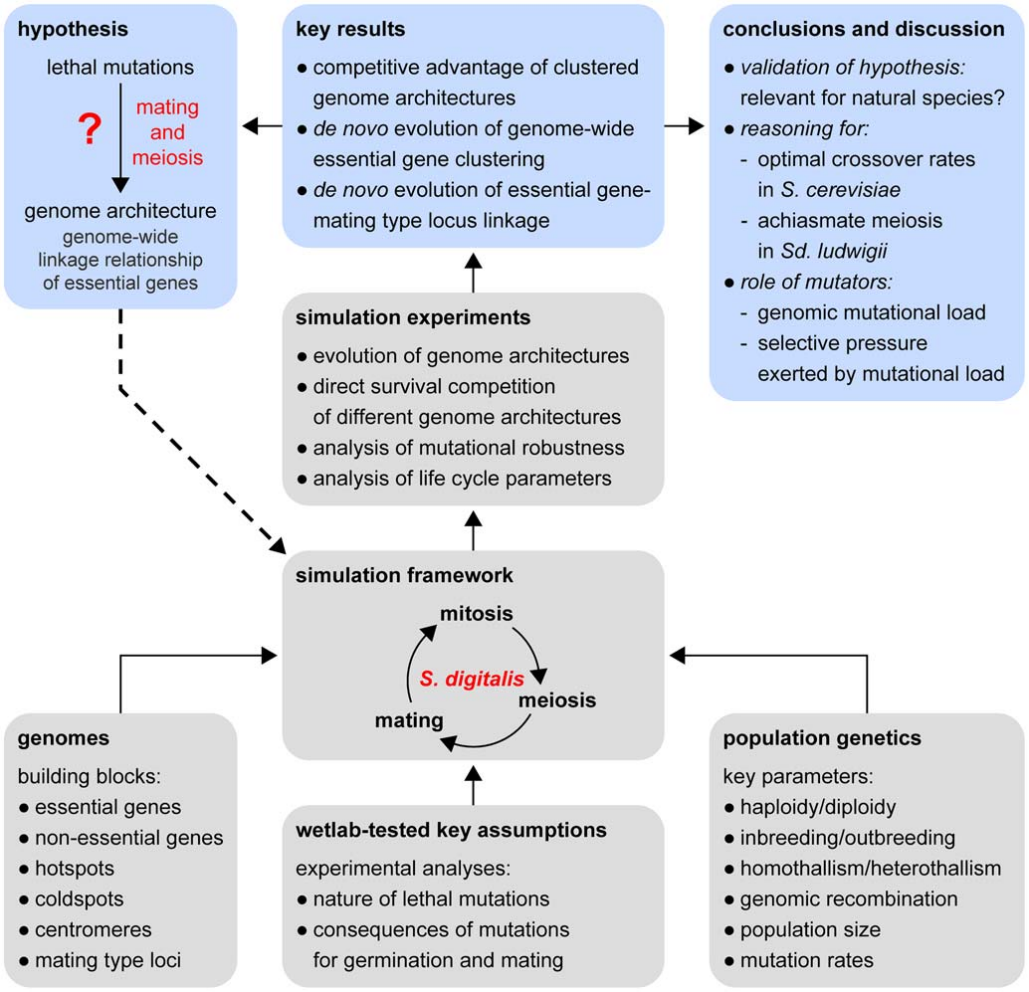
Conceptual overview of the methods and main results of this study. We developed a computer simulation (*S. digitalis*) to test the hypothesis of a feedback between sexual processes (meiosis and mating) and genome architecture that would facilitate purging of lethal mutations in populations of unicellular organisms (such as baker's yeast, *S. cerevisiae*). The implementation of the simulation is based on population genetic concepts and on our results from mutation-accumulation experiments with an Msh2 mutator strain (see main text). Experiments performed with digitally implemented populations of yeast genomes revealed a competitive advantage of yeast-alike chromosome architectures and enabled the evolution of genomes with yeast-alike fitness. These investigations provide reasoning for several hallmarks of yeast genome evolution and for various parameters that describe the population genetics of yeasts.

In order to address this question, we computationally studied the correlation between non-random distributions of essential genes and meiotic recombination with lethal load affecting essential genes. A Monte-Carlo-simulation of breeding diploid yeast populations and chromosome architectures, termed *S. digitalis*, allowed us to investigate the fitness of any genome architecture upon exposure to lethal mutations. We find that several hallmarks of yeast chromosome crossing over during meiosis are consistent with natural selection imposed by recessive lethal mutations affecting essential genes (see Figure 1 for an overview of our approach and the results).

Our simulations imply that lethal phenotypes are frequently caused by single essential gene inactivation. Alternatively, lethal phenotypes may arise from genetic interactions between only weakly deleterious mutations. We explored both possibilities using a conditional yeast mutator strain and analyzed the causes of the accumulation of haploid lethal phenotypes. By determining the global effect on germination and mating, we tested whether the associated load is being transmitted into the next round of diploid growth.

Our combined results suggest an evolutionary history for yeast where sex and meiosis fulfilled a need for efficient purging of mutational load in important/essential genes.

## Results

### Simulation of digital yeast genomes with *S. digitalis*

**Figure 2:**
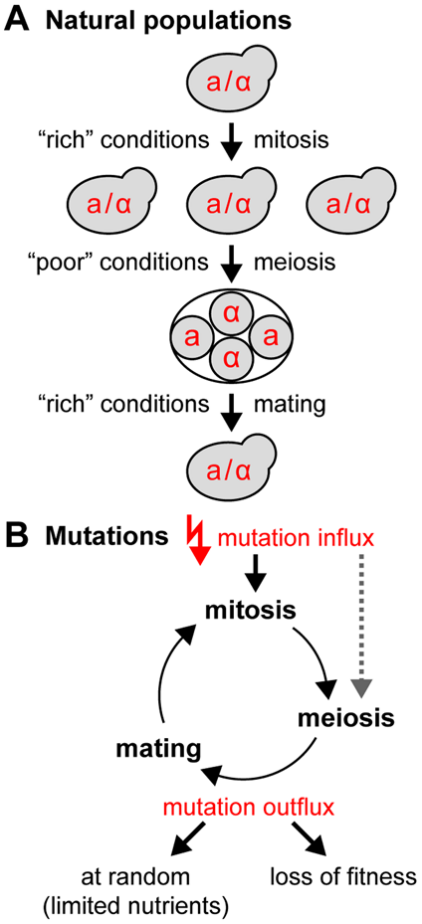
Yeast life cycles and mutation homeostasis. (A) The key elements of natural yeast life cycles. Life cycles in natural yeast populations are regulated by changes in the environment, which either permit mitotic growth or induce the formation of spores via meiosis. All *S. cerevisiae* isolates from nature were found to be diploid, indicating a return to a diploid lifestyle immediately following germination. This may occur by intra-tetrad spore-mating (inbreeding/automixis), or by mating of spores from different tetrads (outbreeding/amphimixis). Optionally, mating type switching followed by mother-daughter mating may generate diploid cell lines (see corresponding sections in Results and Discussion). Predominant diploid life cycles can also be found for many other species throughout the yeasts [91]. (B) Loss of individuals from a population occurs either at random or due to a decrease in fitness caused by mutations. The simulation implements populations that are cycling through vegetative and sexual stages of the life cycle (mitosis, meiosis and mating). The genomes are constantly exposed to mutations. A homozygotisation of recessive lethal mutations (mutations that inactivate essential genes) leads to the death of an individual. Alternatively, individuals may be removed at random from the population due to limitations in the nutritional supply (starvation). In a dynamic equilibrium, in which the average population growth equals zero, the influx of new recessive lethal mutations is equal to the outflux associated with the death or removal of individuals. For more details on the fitness impact of mutations, see main text and Supplementary Figure 1A and 1B in Text S1.

We sought to assay the consequences of non-random distributions of essential genes and meiotic recombination hotspots for the fitness of populations upon frequent inactivation of essential genes by lethal mutations. This analysis requires a direct comparison of the fitness of yeast strains with the same genomic content but different arrangements of the genetic elements. Conducting an experiment such that this question can be addressed in isolation from the many other possible consequences of human-designed genomic architectures is far from trivial. Therefore, we developed a computer simulation of populations of digital genomes subjected to digital life cycles of mitosis, meiosis and mating, modeled according to a simple yeast life cycle (Figure 2A). We used this simulation to study the relationship between genome architecture and the fitness of populations as well as the evolution of genome architectures upon exposure of populations to essential gene inactivating mutations (Figure 2B).

Haploid lethal mutations are frequently observed in yeast and constitute approximately 40% of all deleterious mutations [37],[38] (see also below). The remaining fraction of deleterious mutations has been reported to exhibit a weak impact on fitness [37] and both types of mutations are shielded well in heterozygous diploids [39]. Therefore, we decided to focus on heterozygous lethal mutations, which confer a lethal phenotype either on the level of haploids or upon homozygotisation of mutations in diploids (Supplementary Figure 1A in Text S1).

**Figure 3:**
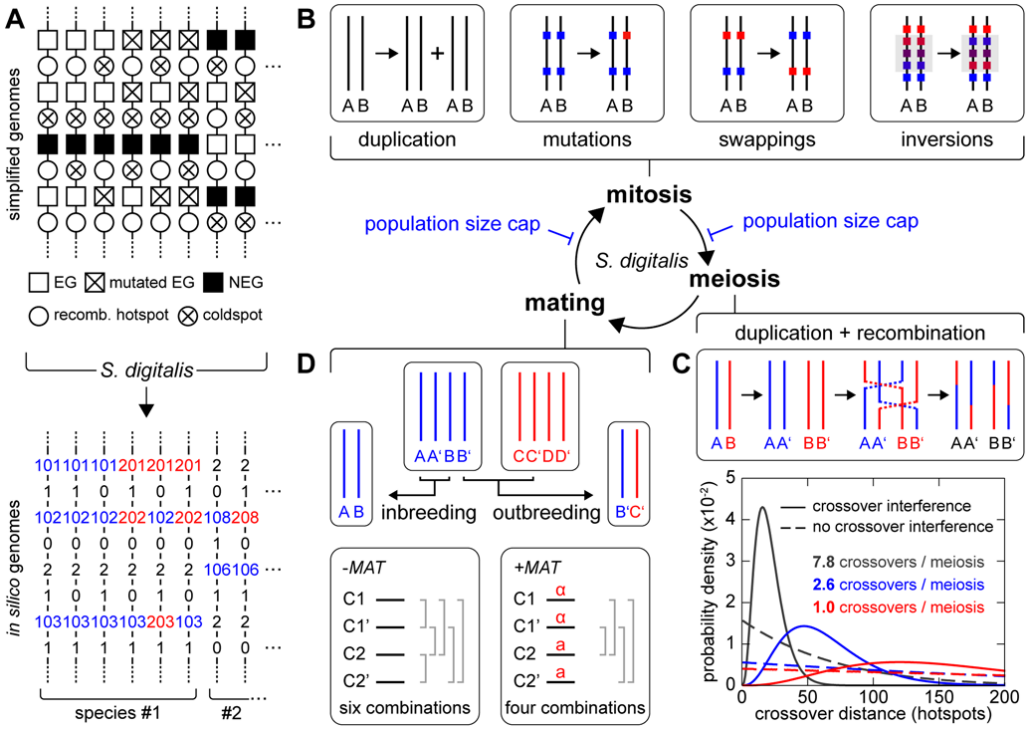
Digital genomes and the life cycle of digital populations. (A) Schematic illustration of the genetic building blocks of the digital genomes (top): essential genes (wild type, white squares; and mutated, crossed squares); non-essential genes (black squares); intergenic elements (circles) are either crossing over proficient (recombination hotspots, white circles) or silent (crossed circles). Unique identifiers represent essential genes. In the simulation, each individual possesses two copies of one single chromosome. Such a diploid genome is considered viable if it contains at least one functional copy of each individual essential gene. The simulation framework implements entire populations of individuals with diploid genomes, which are arranged in a population matrix (bottom). Individuals with different chromosome architectures belong to different species. (B) The life cycle of digital populations. See main text for details. (C) Crossover interference is implemented using an Erlang probability density function with a shape factor *k* = 4 (F. Stahl, personal communication, and references [46],[92],[93]). The function is based on a genetic distance definition (hotspot density). Rescaling of the Erlang function along the distance axis allows adjusting the crossover frequency per chromosome. (D) Breeding can occur between haploid gametes from the same meiosis (intratetrad mating/inbreeding) or between gametes from different meioses (outbreeding). Mating types are optional. See main text for details.

*S. digitalis* simulates populations of diploid digital organisms with one chromosome, which consists of different building blocks: genes, intergenic elements, centromeres and mating type loci (*MATa* and *MATα*). The digital genes are either non-essential or essential. Each gene of the latter category carries a unique identifier. Genes are separated by intergenic elements (IE). IEs are either cold for crossing over ( = coldspots) or hot ( = hotspots). Each feature is represented by an element in the matrix of the population genome, and mutations only affect elements that represent essential genes (Figure 3A). Typical natural yeast chromosomes contain a few hundred genes, and so do our digital counterparts. Populations have a finite size and typically consist of a few hundred to ten thousand diploid individuals.

Mitosis yields a copy of the original genome. It differs from the template genome by mutations, which are introduced at random and lead to the inactivation of essential genes (Figure 3B). The statistical frequency of essential gene mutations per diploid genome and mitosis is given by the genomic recessive lethal mutation rate *R* [40] (for example, *R* = 1 corresponds to an average of one essential gene inactivation per diploid genome and mitosis).

Genomic rearrangements can be simulated. They manifest themselves either as positional swapping of genes and associated intergenic elements, or as segmental inversions (Figure 3B). Genomic rearrangements occur in mitosis and always affect both homologous chromosomes.

In meiosis, the genome duplicates and the homologous chromosomes undergo meiotic recombination (crossing over). The distribution of crossover sites considers meiotic recombination hotspots and crossover interference based on a genetic distance definition (hotspot distribution). The crossover frequency can be adjusted by the shape factor of the Erlang distribution that is used to describe crossover interference (Figure 3C). The four meiotic haploid progenitor genomes constitute a tetrad.

Mutations are allowed to occur in mitosis (see Text S1, section “Supplementary Results and Discussion” for an analysis of meiotic mutations). This implementation considers one single mitotic cycle between consecutive meiotic cycles. This mimics a situation in which many consecutive rounds of mitoses occur without exponential growth. This applies to scenarios where a high loss of individuals occurs (e.g. many individuals eaten by predators or washed away into non-fertile grounds) that keeps the size of a local population more or less constant. Under circumstances where deleterious mutations are recessive and do not influence the fitness of the cells (as indicated by literature [39]), this would lead to the accumulation of mutations during the vegetative period of the life cycle. This simplified scenario should come close to a realistic description of natural *S. cerevisiae* that is consistent with the absence of reports of large natural cultures of *S. cerevisiae* (outside of human-engineered fermentation processes). Moreover, this approximation allows us to simulate large numbers of complete life cycles, which would otherwise be inaccessible due to computational limitations.

Upon germination, the haploid genomes directly engage in mating with other haploid genomes (Figure 3D). Mating can occur between genomes from the same meiosis, which is called intratetrad mating and more generally referred to as automixis or inbreeding. Mating between haploid genomes from different tetrads can also occur, and is referred to as amphimixis or outbreeding. In this article, we use the terms inbreeding and outbreeding to distinguish between the two principal types of mating partner selection in the simulation: mating inside and outside the tetrad. Outbreeding events may nevertheless bring closely related genomes together, simply due to the finite size of the simulated populations. The total fraction of inbreeding matings per round of mating can be specified. Mating optionally considers mating types, of which two exist (*MATa* and *MATα*). The *MAT* locus can be placed anywhere on the chromosome. In this case, the modeled chromosome can be considered to be the sex chromosome. Alternatively, the simulation can employ a virtual second chromosome that contains the *MAT* locus next to its centromere. In intratetrad mating, this causes a linkage of the *MAT* locus to the centromeric region of the investigated chromosome [41].

Diploid genomes with different gene order belong to different species. Individuals from different species are not able to mate with each other. These species represent sub-populations, which emerge in simulation scenarios with genomic rearrangements. Alternatively, different sub-populations can be specified at the beginning of the simulation, e.g. in order to compare the fitness of different genome architectures (species) in survival competition assays.

Fitness of the individuals is assessed in the diploid stage before mitotic or meiotic cell division. We furthermore assumed that mating is not prevented by a haploid lethal mutation in an essential gene (this assumption was experimentally tested; see below, section “Mating rescues genomes associated with lethal mutations”). We decided to use a simple fitness denominator for individuals: a 1 is assigned for diploid genomes that contain at least one functional copy of each essential gene, while a 0 is assigned for genomes, in which both copies of at least one essential gene are non-functional. Individuals with a fitness of 0 are removed. Hence, the only criterion underlying the loss of an individual due to mutations is the homozygotisation of a mutated essential gene. This can occur in two different ways: a new mutation inactivates the second wild type copy or a mating event brings together two chromosomes that both contain a mutated allele at the same position.

Populations were limited in size according to a defined maximum (the population size cap). Excess individuals are removed at random before the next round of mitotic or meiotic division. This simulates limited availability of nutrients. As a result, a selective pressure is introduced that has the potential of driving the evolution of species ( = different genome architectures) that are better adapted to handle lethal mutations. Supplementary Figure 1A and 1B in Text S1 provides an overview of the mechanisms of the simulation.

The simulation provides modules for different types of experiments, including mutational robustness benchmarks of populations with specific genomic architectures, selection advantage assessments with two or more isolated populations that compete for nutrients and evolution experiments of large in- and out-breeding populations that constantly undergo genomic rearrangements (see Text S1). Detailed descriptions of all simulation modules and the implementation of yeast and model chromosomes are provided in Text S1.

### *S. cerevisiae* chromosome architecture provides enhanced mutational robustness

Using *S. digitalis* we first assessed the maximum mutation rate *R* populations with random distributions of genes and recombination hotspots can resist before becoming extinct (the mutational robustness *R*_max_). We compared the results with the mutational robustness obtained for the *S. cerevisiae* chromosome IX architecture, which deviates significantly from a random arrangement of essential genes and meiotic recombination hotspots distribution [30]. This revealed a superior mutational robustness of the yeast chromosome architecture for the entire spectrum of inbreeding fractions (Supplementary Figure 4A; Supplementary Figure 2A in Text S1). We obtained the same result when allowing mutations to occur in meiosis only, or both in mitosis and meiosis (Supplementary Figure 2B and 2C in Text S1).

**Figure 4:**
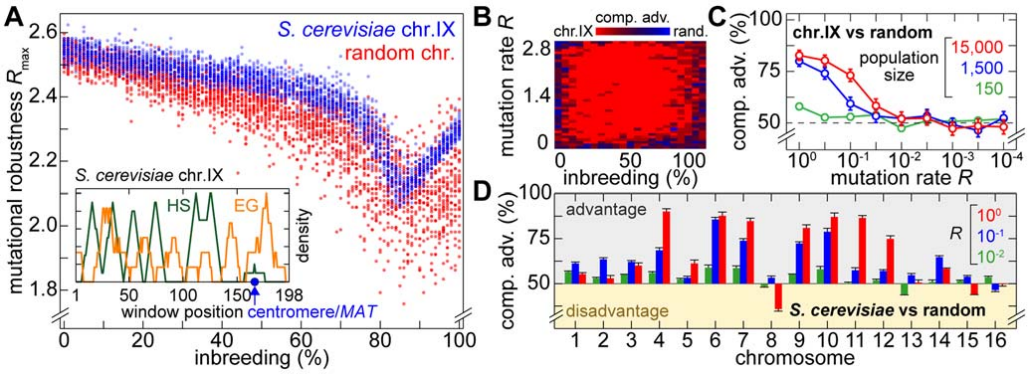
Competitive fitness advantage of yeast chromosomes in the presence of lethal mutations. (A) Mutational robustness *R*_max_ of digital populations with random chromosomes or with *S. cerevisiae* chromosome IX, respectively, at different inbreeding fractions. 3,000 experiments were performed per chromosome configuration. A different random architecture composed of the same total number of meiotic recombination hotspots and essential genes was generated for each reference experiment. A histogram summarizing these results is provided as Supplementary Figure 2A in Text S1. The digitized chromosome IX architecture is based on essential gene data obtained from www.yeastgenome.org as well as on data on the genome-wide distribution of meiotic recombination double strand break sites obtained from [32]. The representation in the inset shows meiotic recombination hotspot (HS) and essential gene (EG) densities of chromosome IX in a sliding window analysis (see Text S1). The population size cap was set to 200 individuals. (B) Results of survival competition experiments for *S. cerevisiae* chromosome IX versus randomly generated chromosome architectures (*n* = 10 experiments per grid point), for the entire inbreeding/outbreeding domain and for high mutation rates up to *R_max_*. The level of dominance is color-encoded (bright red: chromosome IX wins in all experiments, bright blue: random architecture wins in all experiments). The population size cap was set to 2,000 individuals. (C) Results of survival competition experiments for the architectures described in (B), for a wide range of mutation rates (in logarithmic steps, *R* between 10^−4^ and 1) and for different population size caps (150, 1,500 and 15,000). The competitive advantage (“comp. adv.”) indicates the percentage of chromosome IX wins over random architectures. This percentage is formed as an average over the entire inbreeding/outbreeding domain (bars indicate SD). (D) Results of survival competition experiments of the sixteen yeast chromosomes versus randomly generated chromosome architectures (*n* = 170 experiments per bar; population size cap: 10,000), averaged over the entire inbreeding/outbreeding domain. Mutation rates are color-encoded; green: 10^−2^, blue: 10^−1^, red: 1. A competitive advantage of 100% indicates that the yeast chromosome architecture always outperformed the random architectures. A value of 50% indicates that the yeast chromosome exhibited a performance identical to that of random architectures. For simulation details and statistical information see Text S1, section “*S. digitalis* Simulation Settings.”

Using a survival competition assay, we directly compared the persistence of populations with random chromosomes and of populations with yeast chromosome IX at different mutation rates and for different population sizes. The competition experiments revealed a clear selective advantage of the yeast chromosome IX architecture for most regions of the investigated parameter space (Figure 4B). A stalemate situation was only observed at low mutation rates *R*<0.01 and for extreme inbreeding fractions (*i* = 0 and *i* = 1) (Figure 4B and 4C). We performed a control experiment to demonstrate that the quantitative outcome of the survival competition assay is unaffected by the choice, in which life cycle state mutations are simulated (mitosis and/or meiosis) (Figure 2D in Text S1).

An analysis of the recently recorded distribution of 4,300 single crossover events in 50 meioses of yeast [42] indicated non-random distributions of crossovers and essential genes for the entire yeast genome. We found that the resulting average level of clustering is more than 2.5σ higher than the level expected for random distributions (Table 1 in Text S1). Using *S. digitalis*, we obtained comparative data by subjecting digitalized implementations of all chromosomes (Text S1) to a survival competition against randomly generated chromosomes. The simulation outcome attests a superior fitness to almost all of the yeast chromosomes (Figure 4D).

Taken together, our data suggest that yeast-like chromosome architectures contain evolved features consistent with selection imposed by lethal mutations.

### *CEN*-linked and peripheral essential gene clusters provide cumulative benefits

**Figure 5:**
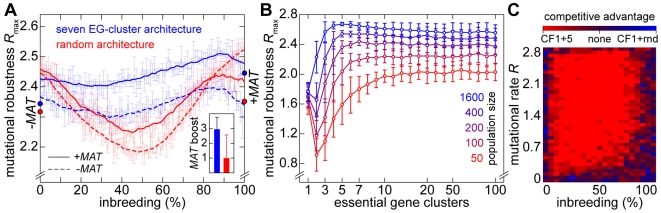
Effect of essential gene clustering on mutational robustness. (A) Mutational robustness *R*_max_ as a function of the inbreeding ratio (*n* = 3 per inbreeding ratio; error bars indicate SD), for a clustered model chromosome (seven EG clusters separated by regions containing meiotic recombination hotspots) and for random chromosomes. Each experiment was performed with a different randomly generated chromosome containing the same number of elements (400 NEGs, 100 EGs, 166 recombination hotspots). The inset shows the average increase of *R_max_* and its SD over the inbreeding/outbreeding domain in response to the introduction of a *MAT* locus. The red and blue dots on the *R_max_*-axis indicate the average over all inbreeding fractions (left: without *MAT*, right: with *MAT*). In clustered model chromosomes, the *MAT* locus was placed in the first EG cluster; in random chromosomes, the *MAT* locus was placed in the centre of the chromosome. The population size cap was set to 200 individuals. (B) 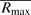 (average of the entire inbreeding/outbreeding domain, bars indicate SD) as a function of the level of EG clustering (ranging from perfect clustering (1) with all EGs joined in one single continuous cluster, to a maximally unclustered architecture (100) with each pair of EGs separated by at least one meiotic recombination hotspot). The population size cap is color-encoded (ranging from 50 to 1,600 individuals). Simulation time was conservatively assigned; a further increase in the maximum number of generations did not change the simulation outcome significantly. (C) Results of survival competition experiments for model chromosomes with one *MAT*-linked cluster and five clusters in the chromosomal arm region (CF1+5) versus model chromosomes with one *MAT*-linked cluster and a random EG distribution in the arm region (CF1+R), for the entire inbreeding/outbreeding domain and high mutation rates up to *R_max_* (*n* = 10 experiments per grid point). The competitive advantage is color-encoded (bright red: CF1+5 won all competitions, bright blue: CF1+R won all competitions). The population size cap was set to 1,000 individuals. For simulation details and statistical information see Text S1, section “*S. digitalis* Simulation Settings.”

The fitness advantage for the structure of chromosome IX relative to randomly structured chromosomes may result from essential gene clusters that are either centromere-linked or peripheral to the chromosome, or cumulatively from both. We designed synthetic chromosome architectures to discern the potential impact of these structural relationships. First, in simulations without *MAT* loci, the highest average *R*_max_ were obtained for synthetic chromosomes, in which essential genes were distributed in a few large clusters (Figure 5A). Moreover, the variability of *R*_max_ as a function of the inbreeding ratio was larger in the case of the chromosomes with less essential gene clustering. The number of clusters providing the best performance depends on the population size: the larger the population the smaller the optimal number of clusters the pool of essential genes must be distributed to (Figure 5B). For clustered architectures, the introduction of a *MAT* locus (which constitutes an obligatory heterozygosity) into one of the clusters increased the persistence compared to genotypes without a *MAT* locus (Figure 5A, inset; and Supplementary Figure 3 in Text S1). Using survival competition assays in genomes with *MAT*-linked clusters, we compared the fitness of genotypes with peripheral essential genes either in clusters or in random distributions. We obtained a fitness advantage of peripheral clustering for a wide range of inbreeding ratios and mutation rates (Figure 5C). Thus, *MAT*-centromere-linked clusters and peripheral essential gene clusters provide cumulative fitness benefits.

### Achiasmate meiosis enhances the mutational robustness of digital genomes

**Figure 6:**
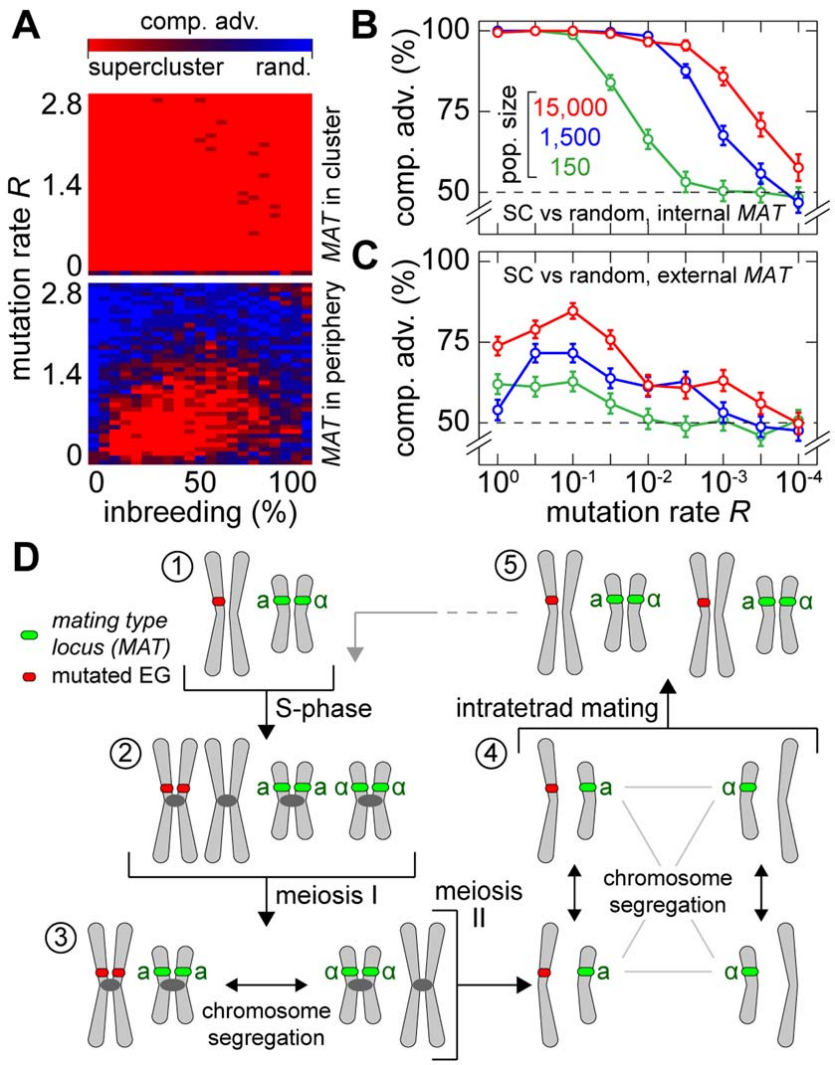
Fitness advantage of achiasmate meiosis. (A) Results of survival competition experiments for random chromosomes versus achiasmate chromosomes (super-cluster architectures) with all EGs in one cluster not linked to (top panel) or linked to the *MAT* locus (lower panel). The matrix shows the color-encoded level of dominance for the entire inbreeding/outbreeding domain and for high mutation rates up to *R_max_*. The population size cap was set to 2,000 individuals. (B) Results of survival competition experiments for achiasmate chromosomes versus random chromosomes, for a wide range of mutation rates (in logarithmic steps, *R* between 10^−4^ and 1) and for different population sizes (150, 1,500, and 15,000). The *MAT* locus was placed on the same chromosome. The competitive advantage indicates the percentage of achiasmate chromosome wins over random architectures. This percentage is formed as an average for all inbreeding/outbreeding ratios (bars indicate SD across the inbreeding/outbreeding domain). (C) As for (B), but with the *MAT* locus positioned next to the centromere on a different chromosome in the genome. (D) Autosomal chromosomes are linked to the *MAT* locus via their centromeres in the case of intratetrad mating. The cartoon illustrates a simple scenario with one autosomal chromosome and the sex chromosome. The autosomal chromosome contains a heterozygous mutation (1). The mutation may be linked to the centromere; this is always the case for achiasmate meiosis. Also the *MAT* locus may be linked to the centromere; this is the case for *S. cerevisiae* and for achiasmate meiosis. If both loci are linked to the corresponding centromere, intratetrad mating (from (4) to (5)) will involve chromatids that are each derived from one of the homologous chromosomes that are segregated in meiosis I (from (2) to (3)). This will reconstitute the heterozygous situation of all sites in the genome that fulfill this criterion. In population genetic terminology, this type of mating is called intratetrad mating with first division restitution. It has been demonstrated to occur in many species, including some fern, flies and fungi [22] and may have constituted a driving force for the evolution of sex chromosomes. For simulation details and statistical information see Text S1, section “*S. digitalis* Simulation Settings.”

The tight physical linkage of all essential genes into a single, large cluster is not a very likely configuration for natural genomes. However, achiasmate meiosis (the absence of meiotic crossovers) results in a comparable situation, since it genetically links together all essential genes on a chromosome. In the following, we will use the word “achiasmate” to denote the absence of meiotic crossing over between all essential genes present on a chromosome. Meiosis without crossovers has been reported for several species [43] and was also suggested to occur in the hemiascomycete yeast *Saccharomycodes ludwigii* [44],[45]. Using our survival competition assay we found that achiasmate meiosis exhibits a high mutational robustness *R*_max_ (Supplementary Figure 3 in Text S1, genomes with one essential gene cluster and with mating types, +*MAT*) and provides a particularly strong fitness advantage in the entire investigated parameter space (considering the inbreeding fraction *i* and the mutation rate *R*) when a *MAT* was present (Figure 6A, top panel).

Generally, no advantage of achiasmate meiosis would be expected for pure outbreeding. The advantage of achiasmate meiosis observed in our simulations in the pure outbreeding domain can be explained by the finite population size, which implies that all individuals are related to a certain degree.

Without *MAT* linkage to the essential gene cluster, the advantage is reduced, but significant for mutation rates *R*<1.5 and non-extreme inbreeding fractions (0<*i*<1) (Figure 6A, lower panel). Using direct competition, we found a strong advantage of achiasmate meiosis over random chromosomes for mutation rates *R* between 10^−4^ and 1, which further increases with increasing population size (Figure 6B and 6C). In achiasmate meiosis, linkage to the *MAT* locus either occurs physically (on the chromosome where the *MAT* locus is located) or via the centromeres (for all other chromosomes due to intratetrad mating). This preserves heterozygosity at autosomal centromeres (see Figure 6D). The resulting selection advantage may provide population-genetic reasoning for the secondary loss of meiotic crossing over in *Saccharomycodes ludwigii*.

### *De novo* evolution of essential gene clustering in complex chromosomes

Our experiments demonstrated a fitness advantage of clustered chromosome architectures when exposed to deleterious mutations. This fitness advantage is the result of the cumulative effects arising from pre-existing essential gene clusters, but it does not allow us to deduce whether clustered genomes can evolve from unclustered or random architectures solely due to the exposure to lethal mutations. For example, alternative and potentially synergistic mechanisms are conceivable (see Discussion). In order to constitute a driving force, the presence of deleterious load would have to cause the emergence of clusters in a self-organized manner, exclusively based on the rules that govern the evolutionary process of genomes in the context of unicellular breeding populations subjected to deleterious mutations.

**Figure 7:**
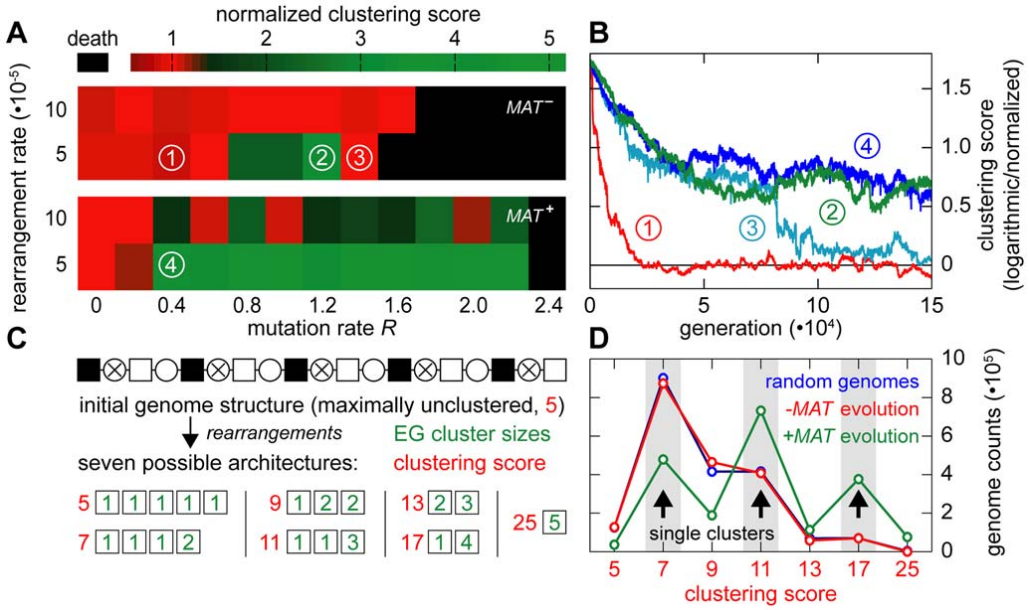
Maintenance and *de novo* evolution of essential gene clustering. (A) Maintenance of EG clustering in inbreeding populations. Initially, EGs and meiotic recombination hotspots were arranged in two separate clusters. Chromosomes were allowed to rearrange by positional swapping of genes and meiotic recombination hotspots during an evolution period of 150,000 generations. The level of essential gene clustering was determined at the end of each experiment using the clustering score analysis (as described in Text S1). Color scheme: green indicates significant clustering after 150,000 generations. A high clustering score indicates a high level of essential gene clustering in regions of low meiotic recombination hotspot density. (B) Time-course of the clustering value in four different regimes in (A). The evolution of populations 1 and 2 is visualized side by side in Video S1. (C) Schematic illustration of a small model genome with five essential genes, which we designed to study the mechanisms involved in the evolution of clustering (see (D)). The clustering scores (red) of all possible architectures (green) are shown below the scheme. Symbols: light box = EG; black box = NEG; circle = meiotic recombination hotspot; circle with cross = meiotic recombination coldspot. (D) Distributions of clustering scores in inbreeding populations evolved from the initial genome shown in (C). The simulation was performed in the presence of a *MAT* locus (green line) as well as without a *MAT* locus (red line) (*n* = 1,000 experiments for each configuration). The distribution of clustering scores for random architectures is shown in blue and serves as a reference (*n* = 2·10^6^). In the presence of a *MAT* locus, clustering evolved over a broad range of rearrangement rates (Supplementary Figure 4 in Text S1). For simulation details and statistical information see Text S1, section “*S. digitalis* Simulation Settings.”

In our investigation of the *in silico* evolution of clustered genomes, we first dissected the complementary process: the maintenance of essential gene clusters in the context of chromosomal rearrangements and the linkage of the *MAT* locus to a gene cluster. We designed an initial genome that contained all essential genes in one large cluster and all meiotic recombination hotspots outside this cluster. Scenarios with and without *MAT* loci were considered in an inbreeding-only domain (full intratetrad mating), which is least favorable to successful persistence of the lineage in the situation without a *MAT* (see Figure 6A). For +*MAT* scenarios, the *MAT* locus was placed outside the essential gene cluster. Populations were evolved using different rearrangement rates (*r*). For rearrangement rates *r*<10^−4^, simulated architectures both with and without *MAT* loci preserved highly significant levels of essential gene clustering and anti-correlated meiotic crossover distributions over periods of at least 150,000 generations (Figure 7A and 7B, Video S1). Without *MAT*, high preservation of essential gene clustering was only observed in a relatively narrow range of mutation rates (0.6<*R*<1.4). In the presence of *MAT* loci, however, clustering was well maintained over a significantly broader range of mutation rates (*R*≥0.2) and also for higher rearrangement rates (Figure 7A). We found that the mating type locus had always relocated to a position inside the cluster by stochastic rearrangement (usually within the first 1,000 generations, *n* = 20 experiments) and had remained in the cluster afterwards. This experiment demonstrates that a significant level of clustering can be preserved in the presence of lethal mutations. Less well-clustered architectures that arise in the presence of destructive forces (genomic rearrangements) are quenched due to a selective pressure towards the better-performing clustered architectures, as long as the rearrangement rate is not too high.

In the next step, we investigated the *de novo* evolution of *MAT*-linked essential gene clusters in small model genomes containing five essential genes, five non-essential genes and four recombination hotspots (Figure 7C). We found that genomes evolved *MAT* linked essential gene clusters over a broad range of mutation rates and rearrangement rates, which is consistent with the fact that inbreeding preserves *MAT*-linked heterozygosity (Supplementary Figure 7D; and Supplementary Figure 4 in Text S1). Architectures with a single cluster (2+1+1+1, 3+1+1, 4+1 or 5 genes) were consistently favored over multi-cluster architectures (2+2+1 or 2+3 genes) (Figure 7C and 7D; grey rectangles indicate single-cluster architectures in Figure 7D).

**Figure 8:**
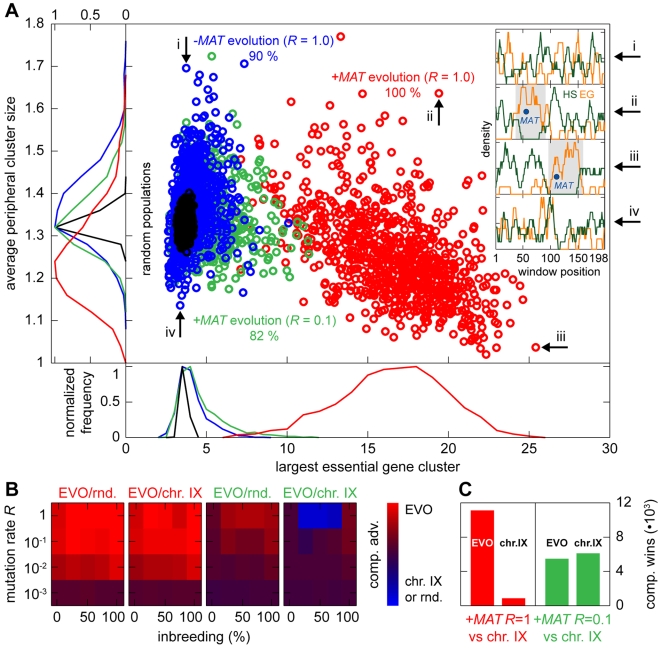
Evolution of yeast-like essential gene clustering. (A) Evolution of chromosome architectures with a yeast chromosome IX-like size and genetic content. Beginning with completely unclustered chromosomes, populations with and without *MAT* were allowed to evolve at different mutation rates *R* as indicated (*n*_red_ = 830, *n*_green_ = 826, *n*_blue_ = 839 experiments). Clustering was scored by measuring both the size of the largest essential gene cluster and the average size of the remaining clusters. For genomes containing a *MAT*, the largest cluster was always observed to be linked to the *MAT*. The scatter plot shows the scores obtained after an evolution period of 100,000 generations. Score distributions for the different populations are spanned along the axes (including reference distributions for randomly generated populations of the same size). The “clustering score” *ν* quantifies the level of essential gene clustering. It is defined as the sum of squared sizes of all essential gene clusters (maximum-sized groups of essential genes not disrupted by a hotspot), normalized by the total number of essential genes and meiotic recombination hotspots. Random architectures with a chromosome IX genetic content score *ν* = 107±17 (SD), whereas the chromosome IX architecture itself scores *ν* = 224. The percentages indicate the fraction of experiments yielding genomes with a clustering value *ν* at least 2σ above the average score of randomly generated genomes (*ν*≥141). The insets show meiotic recombination hotspot and essential gene densities obtained by sliding window analyses of four selected genomes (indexed i to iv). (B) Survival competition of the evolved genomes shown in (A) versus chromosome IX and random genome architectures. The matrices show the average statistical results for all evolved genomes of the green and red groups in (A). (C) The bars indicate the total number of wins of the evolved architectures and of chromosome IX respectively in the competition experiments shown in (B). For simulation details and statistical information see Text S1, section “*S. digitalis* Simulation Settings.”

In order to investigate the evolution of large yeast-like chromosome architectures we switched to domains with 50% inbreeding, since no advantage of chromosome-peripheral clustering was apparent for the extreme breeding domains at *i* = 0 and *i* = 1 (Figure 4 and Figure 5C). For this series of experiments, we implemented a species barrier in the simulation (see also first section of Results, and Text S1, section “Supplementary Results and Discussion”). Thereby, each genomic rearrangement leads to the formation of a new species. This scenario mimics reproductive isolation due to meiotically incompatible chromosomes. A new species might eventually dominate the population or become extinct depending on the reproductive success arising from the fitness (dis-)advantage of its particular genome architecture. We found that the simple life cycle of mitosis, meiosis and mating was sufficient to reproducibly evolve genomes with *MAT*-linked as well as non-random peripheral essential gene distributions (Figure 8A and Video S2). In order to obtain good statistics on this phenomenon, we parallelized the assay by using grid computing, which allowed us to simultaneously evolve many unrelated populations (*n* = 3,000 experiments). On average after 15,000 generations, high-*R* +*MAT* populations reproducibly evolved a level of clustering 2σ above the mean level encountered in random architectures. The evolution of the same level of clustering in low-*R* +*MAT* populations and in −*MAT* populations required a 2–3 fold longer period. Importantly, all high-*R* +*MAT* populations and even a small fraction of the other populations eventually arrived at the level of clustering of the natural yeast chromosome IX (emerging after 30,000–70,000 generations, statistics are provided in the legend of Figure 8A). Genomes with one *MAT*-linked cluster dominated at high mutation rates (*R* = 1), whereas genomes with several clusters, one of which associated with the *MAT* locus, typically evolved at lower mutation rates (*R* = 0.1).

We must point out that the parameter space explored in the evolution of clustering constitutes a compromise enforced by limitations in computation time. The computation of all events during one generation requires approximately ten seconds of CPU time in a population of 4,000 individuals. In order to be able to perform a statistically meaningful number of experiments under different conditions, we applied relatively high rearrangement rates close to a “destructive regime”, in which any emerging cluster quickly became scrambled. This setting allowed us to simulate genomic restructuring as would quantitatively occur over long evolutionary timescales using reasonable amounts of computation time. However, up-scaling the rearrangement rate also necessitates up-scaling the mutation rate, in order to arrive at a selective pressure on par with the potential destructive force introduced by the random rearrangements. If computation time was unlimited, we would also expect a qualitatively comparable outcome for lower values of *R* in the context of lower rearrangement rates.

We were able to qualitatively reproduce our results with respect to the evolution of essential gene clustering in three additional series of experiments. In these experiments, we provided the simulation framework with unclustered architectures assembled from the genetic building blocks of *S. cerevisiae* chromosomes VI, VII and X (Supplementary Figure 5A in Text S1 and data not shown). Even in large chromosomes with chromosome VII- and X-like sizes, highly significant levels of essential gene clustering were reproducibly established. Moreover, similar results were obtained when using an externally linked mating type locus as well as when using lower rearrangement rates over longer evolution periods (*r* = 10^−5^ for 200,000 generations, Supplementary Figure 5A in Text S1).

Taken together, these *in silico* experiments demonstrate that deleterious mutations inactivating important genes can provide a sufficient driving force to reproducibly evolve chromosome architectures resembling their natural counterparts with respect to essential gene distributions, meiotic recombination hotspot distributions and *MAT*-centromere linkage. Therefore, a recurrent exposure to lethal mutations can select for genome architectures in order to account for the associated load in the context of a sexual cycle.

### Competitive advantage of *de novo* evolved genome architectures

Any evolutionary process, when successful, should generate individuals that perform better under the conditions of their evolution than their ancestors. In order to determine the level of success of our evolutionary simulation, we performed survival competition experiments between the evolved genomes discussed above and random chromosomes or yeast chromosome IX. We observed a strong fitness advantage of +*MAT* genomes evolved at *R* = 1.0 when competing with random chromosomes and yeast chromosome IX (Figure 8B). As expected from the presence of large *MAT*-associated essential gene clusters in these genomes, the fitness advantage results for a wide range of mutation rates (*R*≥10^−3^) and for the entire inbreeding/outbreeding domain. The genomes of +*MAT* populations evolved at *R* = 0.1 also performed significantly better than random architectures, but only slightly better than the chromosome IX architecture, with the exception of mixed breeding ratios and high mutation rates (*R* = 1.0), for which chromosome IX performed better. In the statistical average, low-*R* +*MAT* populations exhibited almost the same overall performance as the chromosome IX architecture (Figure 8C).

To further expand this analysis, we also performed competitions of the chromosome X-like products of the evolution experiment shown in Supplementary Figure 5A in Text S1 with random architectures as well as with the actual *S. cerevisiae* chromosome X, using a chromosome description derived from [42]. The results of the competition experiments were qualitatively comparable to those obtained for chromosome IX (Supplementary Figure 5B and 5C in Text S1). The reproduction of our results in the context of chromosome X is particularly striking, since the digitalized chromosome X architecture exhibits the highest level of essential gene clustering of all sixteen yeast chromosomes (see Table 1 in Text S1) and therefore constitutes a particularly challenging opponent for the evolution products in the survival competition.

We conclude that, in the context of our reference life cycle, architectures with chromosome IX-like purging evolve in the regime characterized by the lower mutation rate. The high-*R* regime promotes the evolution of single large gene clusters, the extreme of which represents achiasmate meiosis.

### Mating type switching influences the lethal load in populations

So far, we have investigated the correlation between lethal mutations in essential genes and the parameters that govern population fitness with respect to crossing over and population genetics via simulations of simple life cycles. There are also other processes that may influence the lethal load present in populations (see also Discussion). In particular, mating type switching followed by mating of daughter cells (termed “haplo-selfing”) with their respective mothers is a prominent feature of the life cycle of *S. cerevisiae* (but not of all yeasts, see Discussion). Mating type switching leads to a homozygous diploid, but only if it involves a haploid genome that is free of lethal mutations. Hence, if occurring at significant rates, haplo-selfing would be expected to decrease the lethal load in populations. However, if the mutagenic load in the population is too high, there is only a small probability of generating viable diploids by haplo-selfing. Comparing pre-loaded chromosomes with and without haplo-selfing revealed a sharp transition of the competitive advantage in favor of non-switching populations for a load higher than two lethal mutations per diploid (Supplementary Figure 6 in Text S1). In this regime, already a small percentage of diploidisation via haplo-selfing (2%) constituted a strong disadvantage.

**Figure 9:**
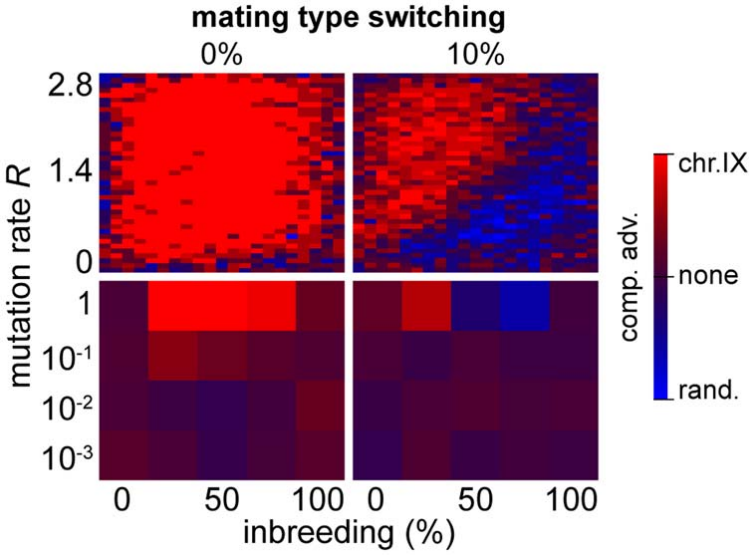
The effect of mating type switching on population fitness. Results of survival competition experiments for *S. cerevisiae* chromosome IX versus randomly generated chromosome architectures (*n* = 10 (top row) or 100 (bottom row) experiments per grid point), in the presence (10%) and absence of mating type switching. Different breeding conditions and mutation rates (between 10^−3^ and *R_max_*) were considered. The matrixes in the top row show the outcome of a high-resolution analysis of the high-*R* regime, for which the most pronounced differences were observed. The level of dominance is color-encoded (bright red: chromosome IX wins in all experiments, bright blue: random architecture wins in all experiments). The population size cap was set to 2,000 individuals. For simulation details and statistical information see Text S1, section “*S. digitalis* Simulation Settings.”

To assay the impact of haplo-selfing on the fitness of different chromosome configurations, we performed several analyses. First, we compared the mutational robustness (*R*_max_) of chromosome IX and of random chromosomes for different levels of haplo-selfing. A high haplo-selfing rate of 50% leads to a significant increase in the mutational robustness *R*_max_, both for random architectures and for chromosome IX. At the lower rate of 10% haplo-selfing, the advantage of the chromosome IX architecture remained, but the mutational robustness decreased as compared to the situation without haplo-selfing (see Supplementary Figure 2A and 2E in Text S1). We further performed competitive advantage experiments of chromosome IX vs. random chromosomes at 10% haplo-selfing and for mutation rates between 10^−3^ and *R*_max_. In the absence of a mutagenic pre-load, we noticed the emergence of a phase transition in parameter space at high mutation rates, indicating a region that is dominated by random architectures (Figure 9). However, even in this extreme scenario the chromosome IX architectures, on average, still performed better than the random architectures (57% competition wins of chromosome IX vs. 43% competition wins of random architectures).

### Meiotic crossover rates in *S. cerevisiae* are optimal

**Figure 10:**
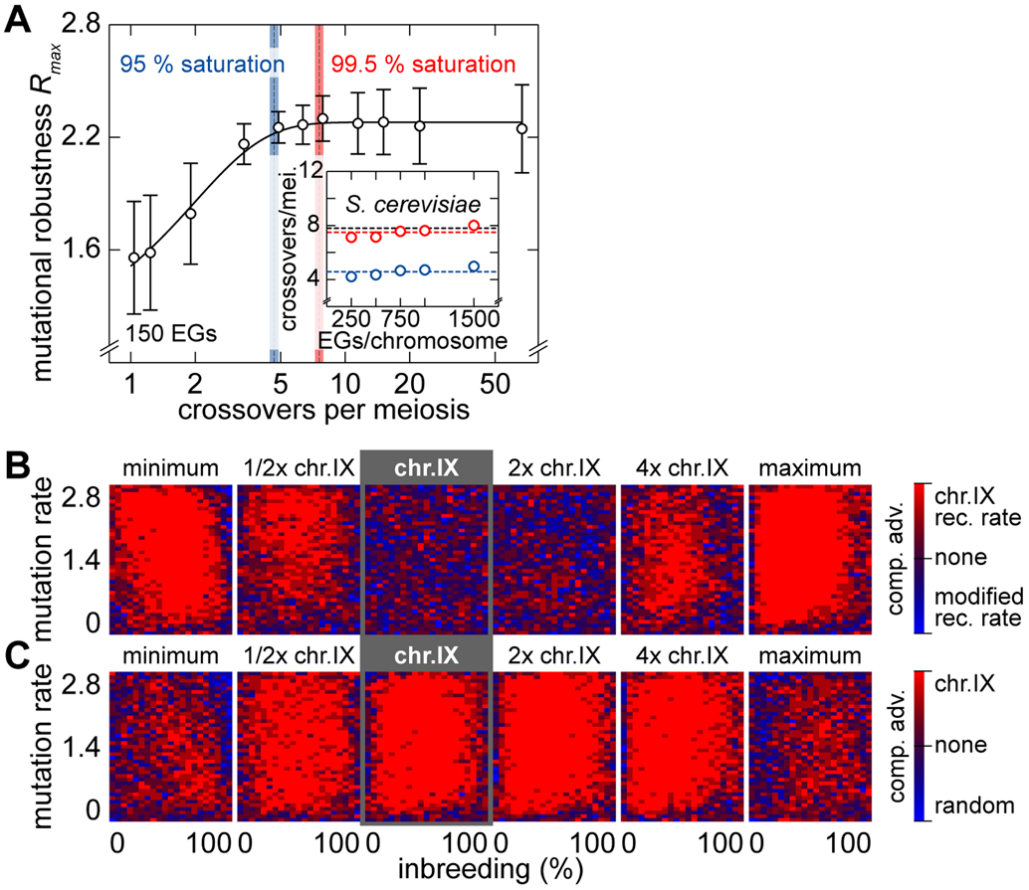
Natural crossing over rates for yeast chromosomes are optimal. (A) 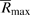 as a function of the number of crossovers per meiosis (logarithmic scale) for populations of random chromosomes with 750 genes (20% EGs). Bars indicate the SD of *R*_max_ for the entire inbreeding/outbreeding domain. Inset: Number of crossovers per meiosis required to reach 95% and 99.5% respectively of the maximum mutational robustness *R*_max_, for chromosomes of different lengths (250, 500, 750, 1,000, and 1,500 genes) and random configurations. (B) Results of survival competition experiments between two chromosome IX populations subjected to different crossing over rates (*n* = 5 experiments per grid point), for mutation rates *R* up to *R_max_* and for the entire inbreeding/outbreeding domain. The color-code indicates the winning chromosome architecture. (C) Results of survival competition experiments between yeast chromosome IX and random chromosomes at different crossing over rates (*n* = 10 experiments per grid point). Same experimental conditions as in (B). Supplementary Figure 8 in Text S1 shows plots for average fitness advantages of the results in (B) and (C). “minimum” = one crossover per chromosome and meiosis; “maximum” = all 58 hotspots are active in each meiosis. For simulation details and statistical information see Text S1, section “*S. digitalis* Simulation Settings.”

Using *S. digitalis*, we determined the influence of the meiotic crossover rate of chromosomes on fitness and on the ability to purge mutational load. In yeasts, crossover rates vary considerably, from 0 (achiasmate meiosis) up to approximately 20–40 crossovers per chromosome in *S. pombe*. The genomic mean for all *S. cerevisiae* chromosomes is 5.6 crossovers per meiosis (derived from the genetic map, www.yeastgenome.org) [46]. This value was confirmed by the direct assessment of crossover frequency and distribution [42]. The chromosome-specific number of crossovers scales linearly with the number of genes (*R* ^2^ = 0.89), in a range of 2.5–9 crossovers for individual chromosomes (Supplementary Figure 7 in Text S1). We used random genomes of different sizes (250–1,500 genes) to assess the mutational robustness *R*_max_ as a function of crossover frequency and inbreeding fraction. This revealed an increase of *R*_max_ with increasing crossover rates, reaching saturation levels (95–99.5%) in the range of 4.5–8 crossovers. The observed variability of *R*_max_ as a function of the inbreeding fraction was minimal in the 95–99.5% saturation interval (Figure 10A). A slight dependency on chromosome length was apparent (Figure 10A, inset). Direct competition of chromosome IX populations subjected to the natural crossover rate with chromosome IX populations at modified crossover rates demonstrated that crossing over rates higher or lower than the naturally observed average lead to a decrease in the fitness advantage (Figure 10B; see Supplementary Figure 8 in Text S1 for a plot of the average performance). Moreover, when assessing competition experiments of chromosome IX versus random architectures as a function of the crossover rate, chromosome IX performed best in a regime of yeast-like crossing over rates (Figure 10C; see Supplementary Figure 8 in Text S1 for a plot of the average performance). This suggests that the natural rates of meiotic crossovers in *S. cerevisiae* are adapted to handle deleterious load.

### Lethal mutations are frequently associated with single loci

Haploid lethal phenotypes may be caused by mutational inactivation of one essential gene, or they may arise as a consequence of cumulative effects of non-lethal mutations in essential and non-essential genes. Dissecting synthetic lethal relationships demonstrated a 0.8–4% chance of lethality when two non-essential genes are deleted [47]; a simple extrapolation predicts a 50% chance for a lethal phenotype upon inactivation of approximately 7–14 non-essential genes, assuming scalability (and ignoring the possibility of non-linear network properties, such as positive epistasis [48]). In this case, however, most of the cells would have died before reaching such a high load, due to inactivation of one of the 19% essential genes. This rather empirical analysis might indicate that many haploid lethal phenotypes are caused by inactivation of a single essential gene, as we hypothesized for the purpose of our investigation.

In order to test this hypothesis directly, we generated a diploid strain containing *MSH2* under control of the weak and fully repressible *GalS* promoter in order to perform mutation accumulation experiments. Deletion of the mismatch repair gene *MSH2* leads to greatly elevated levels of point mutations [49],[50]. Additionally, reduced sequence specificity for homologous recombination was observed [51],[52], leading to increased levels of recombination between similar sequences at separate loci (ectopic recombination). Mismatch repair deficient strains accumulate high levels of lethal mutations, which are however not associated with an increase in gross chromosomal rearrangements [53].

**Figure 11:**
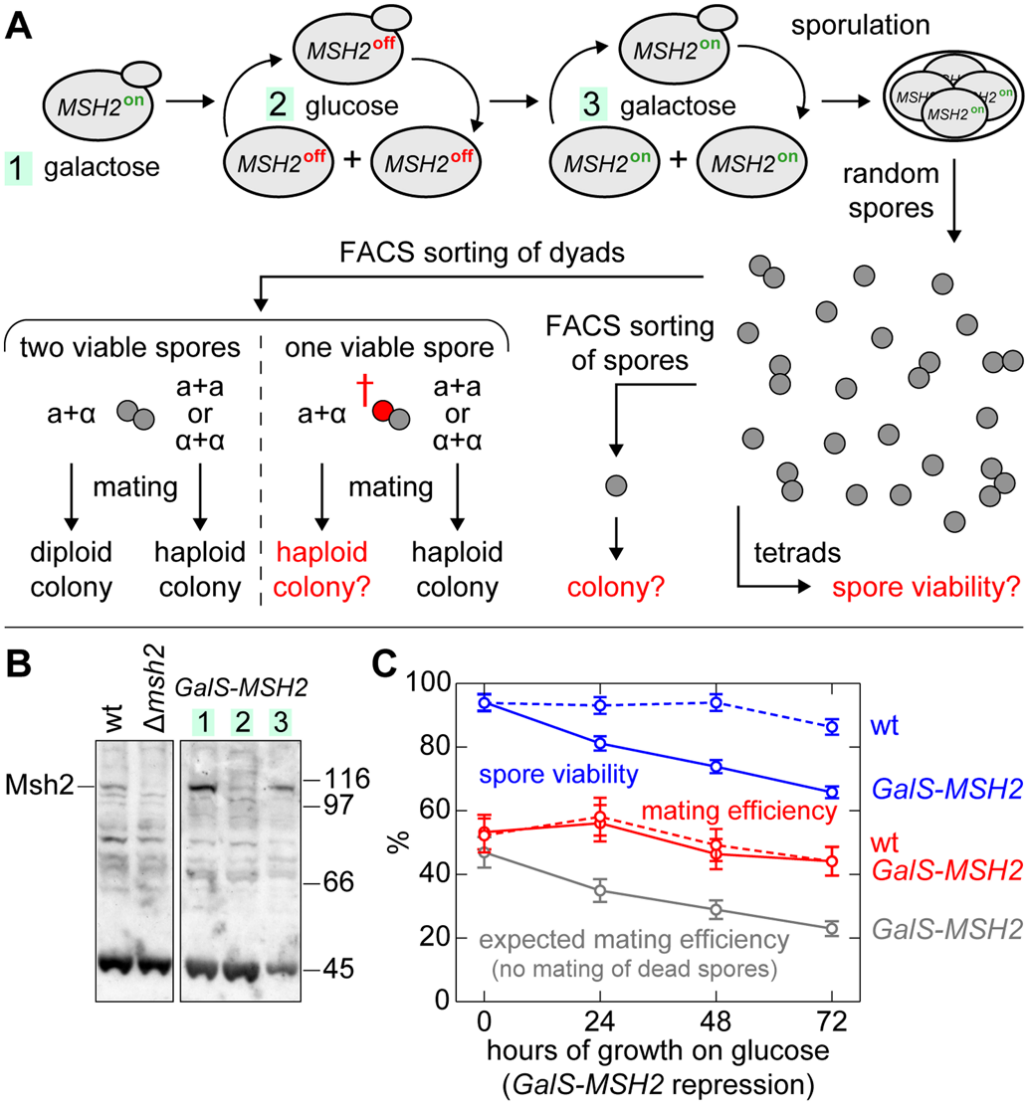
Mutation accumulation and genetic analysis of lethality and mating. (A) Outline of the mutation accumulation experiment. Diploid *GalS-MSH2/GalS-MSH2* cells were grown in the presence of glucose to repress *MSH2* expression (*MSH2* ^off^) for three growth periods of 24 hours (1/1000 dilution between the cultures). After each growth period, an aliquot was removed and the *GalS* promoter was induced by spreading the cells on galactose-containing plates for 24 hours in order to prevent a further accumulation of mutations. Upon sporulation, the products of meiosis were analyzed for viability using tetrad analysis (results are given in the main text) and sorting of single spores or dyads by FACS. The ability of mating was analyzed by testing the mating types of sorted dyads in order to determine whether the formed colonies were haploid (one spore unviable, no mating) or diploid (mating). (B) Msh2 levels in *GalS-MSH2* and control cells were grown first on galactose (1), then on glucose for 24 hours (2) and finally on galactose again for 6 hours (3); detection via Western blotting and antibodies specific to Msh2 (Santa Cruz, Msh2 (yC-15): sc-26230). (C) Viability of spores and dyads. For each time point 1,536 spores and 384 dyads were FACS-sorted in array patterns onto YP-Gal plates (140 mm diameter). Spore viability was determined by counting the number of formed colonies. Mating efficiency of spores in dyads was determined by counting the fraction of the colonies that consisted of diploid cells. In wild type cells and at 0 h in the *GalS-MSH2* strain, this fraction was 53%. The probability of having two viable spores of opposite mating type in a dyad was calculated from the time-dependent spore viability and from the fraction of diploid formation at 0 h. These data allowed us to calculate the probability of diploid formation (mating), if unviable spores were unable to mate (dashed line).

Growth of this conditional mutator strain on glucose-containing medium led to depletion of Msh2 from the cells (Figure 11A and 11B) and to the accumulation of mutant phenotypes with reduced viability. In order to restrict the accumulation of mutations to vegetative growth, we shifted the cells to galactose-containing medium to induce *MSH2* expression prior to sporulation (Figure 11A), which also prevents aberrant post-meiotic segregation events and ectopic recombination associated with the *MSH2* deletion [54]. When grown exclusively on galactose-containing medium, the *GalS-MSH2* strain exhibited wild type spore viability (Figure 11C). Upon mutation accumulation for approximately 30–36 generations (three growth periods of one day each), tetrad analysis (*n* = 400) revealed little 3-spore or 1-spore viability (8% and 15.5%). The majority of tetrads with unviable spores contained two viable spores (36%), which could be caused by single locus events leading to haploid lethality. Alternatively, some 2-spore viability may have resulted from meiosis I non-disjunction. We tested this option by measuring the linkage of the lethal load in 2-spore viable asci to the heterozygous *leu2/LEU2* locus, which itself is centromere-linked (5 cM), and found that the lethal load exhibited on average only partial linkage to *leu2* (27 cM). Since the load locus is different in each analyzed tetrad, only in approximately 25% of all cases crossing over between the centromere/*leu2* locus is prevented (see Text S1, section “Supplementary Results and Discussion”), either due to tight centromere linkage of the load or as the consequence of homologous non-disjunction in meiosis I. Therefore the prominent fraction of tetrads with two viable spores is likely to be caused by freely segregating single mutagenic events affecting an essential function/gene. Importantly, the frequency of tetrads with only one dead spore was low, indicating low cumulative lethal effects of non-lethal mutations recombined into a haploid genome during meiosis. Taken together, these data suggest that losses of genomes associated with random mutagenic events are frequently associated with single events leading to a lethal haploid phenotype.

### Mating rescues genomes associated with lethal mutations

In this scenario, any linked mutation providing an advantage is lost as soon as the lethal mutation becomes exposed, e.g. by haploid growth following meiosis or homozygotisation. However, the genome with the lethal mutation may be preserved via mating with a spore containing the wild type allele. In order to test whether haploid lethal mutations have a high or a low chance to render spores unable to mate, we investigated the rates of diploid colony formation of single dyads using fluorescence activated cell sorting (FACS) after different mutation accumulation periods (Supplementary Figure 9 in Text S1). The observed frequency of diploid colonies did not change significantly as a function of accumulated mutations, when compared to the wild type. If spores associated with lethal mutations were frequently unable to mate – either due to the lethal mutation itself or due to co-accumulated non-lethal mutations – a greater than two-fold decrease of diploid colonies during the course of the experiment would have been expected (Figure 11C). Consistently, we found that only a very minor fraction of viable spores were impaired in mating and that dead spores would still germinate and often form microcolonies (see Text S1, section “Supplementary Results and Discussion”; and Supplementary Figure 10 in Text S1 for a histogram of the colony sizes). This latter result is also consistent with our observation that the majority of lethal phenotypes are not caused by meiosis I non-disjunction, as spores that lack entire chromosomes usually fail to germinate. Taken together, our results show that the majority of randomly occurring lethal mutations did not prevent the spores from mating.

## Discussion

We investigated the influence of deleterious mutations on the evolution of genome architectures. In breeding unicellular diploid organisms subjected to high mutation rates, the frequent inactivation of important genes selects for non-random distributions of important/essential genes and meiotic recombination hotspots. In simulated experiments architectures evolve, which bear a striking resemblance to the architectures of natural yeast chromosomes and provide a competitive advantage over a wide range of mutation rates.

### Purging of deleterious mutations

The selective advantage of clustered genomes arises from multiple effects that cumulatively improve the fitness of such genomes. Initially, the average mutational load (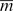) in a simulated population subjected to a mutation rate *R* increases until an architecture-dependent equilibrium is reached. In this equilibrium, the influx of new stochastic mutations is equal to the outflux associated with loss of individuals from the population (Figure 2B). Upon elimination of an individual, all mutations associated with its genome are also removed from the population (Supplementary Figure 1A in Text S1). Within the simulation framework, the loss of individuals occurs via two factors. First, the random removal of excess genomes due to the population size cap is entirely neutral, since it affects all evolved sub-populations with the same probability. Second, and most importantly, genomes are lost due to homozygotisation of a mutant locus. The probability of a homozygotisation via a new mutation during the vegetative division is proportional to the mutational load present in a genome, irrespective of the genome architecture. A higher mutational load in a population therefore lowers its fitness in mitosis due to the increased sensitivity to new mutations, even if the heterozygous load is entirely neutral. Therefore, the only process whose outcome can be improved is meiosis and the loss of individuals due to homozygotisation after mating. Since homozygotisation of any single recessive lethal mutation removes the whole genome from the population, including the associated mutations, the effect of clustering can be explained generally by a reduction of the total number of individuals that are lost after mating. This maximization of mutation purging is caused by the evolutionary optimization of the genome in the context of a sexual lifestyle. Supplementary Figure 11 in Text S1 illustrates a simple scenario.

Clustering increases the level of genetic load that can be maintained in the genome. In order to obtain a positive overall effect, clustering needs to compensate for increased mitotic losses, which in simulations of random chromosome architectures constitute approximately 20% of all homozygotisation losses during one life cycle. In those simulation scenarios, in which a large EG cluster evolved (*R* = 1.0), mitotic losses can reach the same magnitude as meiotic losses (Supplementary Figure 12 in Text S1).

In conclusion, the advantage of clustered genomes must arise from an increase in reproductive fitness in mitosis and meiosis. This results from an optimized balancing of all factors that influence the frequency, with which homozygotisation of a lethal load locus occurs (Supplementary Figure 1B in Text S1 and Supplementary Figure 11 in Text S1). In mitosis, homozygotisation of lethal mutations is dependent on new mutations and the global load in the population, while in meiosis, homozygotisation results after mating. This is influenced by crossover distribution and frequencies, the breeding behavior and the genetic linkage relationship between crossover sites and essential gene loci.

In several experiments, we applied mutation rates higher than the average deleterious mutation rate reported for some *S. cerevisiae* strains [37],[55],[56]. However, as noted in the description of the simulation (see Results, section “Simulation of digital yeast genomes with *S. digitalis*”), we did not assume an exponential growth of our populations, but rather implemented a scenario where species compete for a limited pool of nutrients. In this scenario our implementation of only one round of mitosis between consecutive meioses mimics a scenario of many mitoses without population growth. Although this may not properly describe the growth of a population in a local niche for a short period of time, it certainly does recapitulate longer evolutionary periods that include many consecutive sexual cycles. It is reasonable to assume that natural yeast populations undergo more mitoses than meioses. Hence, if the mutation rate is to be compared to the natural mutation rate, it must be divided by the average number of consecutive mitoses. This number, however, is currently not known.

### Crossing over suppression near centromeres and the hotspot–crossover relation

Crossover suppression near centromeres may also be caused by the interference of pericentromeric crossovers with proper meiotic chromosome segregation [57]. Alternatively, it has been proposed that cold centromeres may be caused by a requirement to protect centromeric repeats [58]. These considerations relate to a general issue in evolutionary biology: frequently, several independent or interacting mechanisms exist or are at least conceivable to explain an observation. As a result, it is often difficult to precisely define the scenario in which one or the other mechanism is dominant and how different mechanisms interact with each other. The situation is further complicated, since there are usually exceptions to each explanation (e.g. species, in which certain aspects are different): yeast does not exhibit centromeric repeats; there are species, in which crossovers occur preferentially in centromere-proximal regions [59], etc. The fact that there are molecular mechanisms that provide support for different scenarios (crossovers close to or further away from centromeres) makes it difficult to deduce the causal connection.

Exploring whether a specific mechanism is singularly able to provide a valid explanation constitutes one strategy to tackling this issue. In the case of *S. cerevisiae*, our evolutionary scenario is able to recapitulate the evolution of crossover suppression and essential gene enrichment in pericentromeric regions. Our simulation provides evidence that lethal mutations and chromosome evolution interact. But, of course, there is no record for the true historical events that enforced this constellation. The complete answer could be given by exploring the (derived) genomes of all ancestors of *S. cerevisiae* along with the evolution of the molecular mechanisms that govern centromeric crossover protection (which is still subject to investigation).

While we took advantage of recent data from Mancera *et al.* [42] in many experiments involving the digitalization of natural yeast chromosomes, our digital implementation of chromosome IX was derived using the hotspot mapping data from Gerton *et al.* [32]. In the meantime, additional hotspot data sets have been published [60],[61]. Using data sets about double strand breaks to predict the distribution of crossovers is accompanied by the limitation that DSB frequencies do not translate linearly into crossover frequencies. In this sense the Gerton *et al.* data set (which was the only data set available at time we began our analysis) was very helpful [32], since it provided a correct description of cold centromeres, as confirmed by the yeast genetic map. Two more recent studies [60],[61] detected more DSBs near centromeres, which, however, do not convert into crossovers (www.yeastgenome.org, see Text S1, section “Supplementary Results and Discussion” for a quantitative assessment of the cM-to-kb relationship in pericentromeric regions). These studies do not deviate so much from the Gerton *et al.* study in the rest of the genome (except for the subtelomeric regions, which are not so relevant in our context).

### The mutator scenario for genome evolution

Natural isolates of *S. cerevisiae* were reported to exhibit large diversity in terms of mutagenic load, yielding a significant fraction of isolates with low to extremely low spore viability. Some proportion of this load, which must have accumulated during mitotic growth and preserved during inbreeding [62],[63], may have been caused by mutator phenotypes. Widespread occurrence of natural variation that can give rise to mutator phenotypes has been reported [64],[65]; this or a similar type of variation may be the reason for the formation of natural yeast strains with highly unstable karyotypes [66]. These may be caused by impaired recombinational repair, as indicated by a certain dependency on Rad52 [67]. Interestingly, these strains can give rise to meiotic offspring with stable karyotypes.

Based on these considerations we speculate that genomic rearrangements, which are associated with the formation of new species or lineages, are frequently also associated with high mutation rates that occasionally – and in particular during the periods that shape the genome – exert a selective pressure by means of high levels of lethal mutations.

The yeast evolutionary history governs several hundred million years and includes many species with (at least nowadays) predominantly diploid life cycles [19],[68],[69]. Although the average frequency of meioses is unknown (from the general perspective as well as for individual species), a more than sufficient number of sexual cycles and genomic rearrangements must have taken place to allow for the maintenance and selection for particular genome architectures.

### Mating type switching and loss of heterozygosity (LOH)

In yeast, mating is a highly favored process that occurs whenever two cells of opposite mating type meet − in dense populations or on the level of the spores of a tetrad [31],[70]. Even considering that *S. cerevisiae* sometimes aborts the formation of one to three spores during sporulation (due to limited availability of nutrients), the overall formation of spores with a mating partner available from within the same tetrad is well above 80% for a broad range of conditions [31]. This circumstance as well as the possibility of mating of spores from different tetrads indicates that mating type switching is most likely not the dominant way of diploidisation in *S. cerevisiae*. Thus, it appears safe to assume that the relative effects we observed at a level of 10% haplo-selfing represents an overestimation of the actual situation, rather than an underestimation.

Mating type switching was one of the key inventions of lifestyle variation that emerged around the time point of the whole genome duplication [68],[71]. One may wonder about the reasons for this evolutionary invention in the first place, and what causes its recurrent secondary loss (mating type switching deficient *S. cerevisiae* may constitute up to 10% of the strains isolated from natural wine fermentation, [72]). Lifestyles seem to evolve towards a preference for diploid stages [73], in which heterozygosity can be maintained. An associated need for efficient purging of load may have influenced the evolution of mating type switching. Occasionally, when mutation rates become too high or meiotic cycles too infrequent, a resulting high lethal load may also be able to select against mating type switching. Diploidy in the context of lethal load can enforce the evolution of very high levels of intratetrad mating in unicellular eukaryotes [23], as observed in the non-switching pre-WGD duplication yeast species *Saccharomyces kluyveri* [74] and *Saccharomycodes ludwigii* [45],[75]. These considerations together do not exclude additional or other roles for mating type switching and intratetrad mating, but they suggest that lethal mutations and mutagenic load may frequently accompany diploid life cycles in unicellular organisms.

Additional mechanisms exist that influence the lethal load in diploid populations. Loss of heterozygosity associated with mitosis acts in a position-specific manner with increased frequency further away from the centromere. It could therefore provide an additional reason for essential gene enrichment in pericentromeric regions. However, its frequency is low (approximately 0.5 to 10 per 10,000 cell divisions in young cells and 50 to 500 in old mother cells [76]) and it is therefore unlikely to significantly influence the global lethal load of the populations.

### “Survival of the flattest” and how to improve further

In a race for beneficial mutations upon exposure to new conditions, essential genes would effectively reduce genetic drift, if their inactivation caused a significant fitness reduction in the diploid organism. Consequently, the chance of acquiring new mutations not immediately accessible to the original genome would decrease, e.g. reducing the frequency of beneficial mutations that are accompanied by a mutation that causes the loss of an essential function.

The occurrence of deleterious mutations is predicted to select for the evolution of properties that increase robustness, even if the evolved genomes have a lower maximum fitness in the mutation-free environment. This is compensated by the higher mean fitness of the variants present in a mutant population, known as “survival of the flattest” [77]. Support is provided by studies of viruses and bacteria, but also by studies using digital organisms [78]. An important body of literature is summarized in Wilke and Adami (2003) [79]).

In yeast, several mechanisms exist that account for buffering mutational load. One is global buffering or positive epistasis of the fitness reduction in combinations of non-essential gene deletions [48]. Essential genes exhibit less expression noise than non-essential genes [80],[81]. Batada and Hurst [36] have proposed that the evolution of essential gene clustering was driven by their accumulation into chromosomal regions of low average nucleosome occupancy (open chromatin), which are domains with lower expression noise and which coincide with the domains of low meiotic recombination [82]. Although this idea is intriguing, no support has been provided that this scenario results in a fitness advantage sufficient to select for the relocation of an essential gene into a cluster. *S. cerevisiae* grows predominantly as diploid. Since the individual intrinsic noise from one copy of a gene is uncorrelated to the noise from the other copy, a lower total noise level is present in diploids as compared to haploids [83]. Selection of low noise for essential genes would thus be less effective in diploids than in haploids. Only about 9% of heterozygous deletion strains of essential genes exhibit haplo-insufficiency in *S. cerevisiae* [84], indicating that low noise may be particularly important in a diploid situation, where one copy of an essential gene is inactivated due to a mutation. This would minimize noise in the context of an overall reduced protein level. This result would suggest that a significant interaction may exist between the low noise model and purging of mutations from diploids, which could act synergistically towards improving the clustering of essential genes.

Proliferation of tumor cells depends on the inactivation of tumor suppressor genes by subsequent multiple mutations. Due to early-acquired mutations in DNA mismatch repair and other pathways required for genetic stability [10], [85]–[87], mutator phenotypes have the potential of accelerating the progression of cancer development by enhancement of the variability upon which Darwinian selection can act [9],[10],[88]. In this scenario, evolved robustness towards haploid lethal load is an important factor that has implications for the understanding of genetic instability in cancer development.

### Conclusions

Purging of deleterious load depends on the tendency to accumulate load and on the presence of alleles that increase the rate at which mutations occur (i.e. mutator alleles). The robustness inherent to the genome architecture of yeast therefore indicates that high mutation rates and genetic load have played an important role during evolution, when the ability to deal with deleterious load co-evolved with better suited genome architectures. Altogether there is increasing evidence that deleterious load is common to evolving yeast populations and that many aspects of the cellular physiology evolved to allow survival as fit but “flat” species.

## Methods

### Yeast mutator experiment

A diploid strain carrying chromosomal *MSH2* under control of the inducible *GalS* promoter was constructed [89] in the well-sporulating SK1 genetic background [90]. The cells were constantly maintained under *GalS* inducing conditions (YP-galactose/raffinose (YP-Gal), see also Text S1, section “Supplementary Methods”) and exhibited wild type spore viability. In order to allow random mutations to occur, the *GalS* promoter was repressed using glucose while cells were grown for 24, 48 and 72 hours (24 hours≈10–12 generations) using serial dilution. At each time point, an aliquot of the cells (5·10^7^ cells) was plated on YP-Gal for 16 hours. The cells were then washed off, plated on a sporulation plate (1% KAc, 0.02% raffinose, 0.02% galactose) and incubated for 40 hours at room temperature. Ascus formation occurred in all cases with frequencies >99%. After disrupting the asci, FACS sorting was used to spot single spores and dyads on large YP-Gal plates (1536 spores or 384 dyads per plate). Sorted spores and dyads were analyzed for colony formation (viability) and mating type (*MATa*, *MATα* and no mating type = diploid cells) using mating type tester strains and a halo assay (Text S1, section “Supplementary Methods;” and Supplementary Figure 9 in Text S1). The viability of the spores from 400 different tetrads was analyzed by tetrad dissection.

### Simulation framework of yeast genome evolution—*S. digitalis*

The source code of the simulation *S. digitalis* is provided as Protocol S1. A detailed description of the simulation is included in Text S1, section “The Computer Simulation *S. digitalis*;” and in Table 2 in Text S1.

## Supporting Information

Text S1 The computer simulation *S. digitalis*; supplementary results and discussion; supplementary materials and methods; supplementary figures and tables; *S. digitalis* simulation settings.
(1.26 MB PDF)

Protocol S1 *S. digitalis* simulation. Matlab source code. Version 1.79 (2005–2009), Philipp J. Keller, EMBL Heidelberg.
(0.05 MB ZIP)

Video S1 Maintenance of EG clustering at low and at high mutation rates. Maintenance of essential gene clustering in evolving inbreeding populations for two of the data points in Figure 7A in Main Text. The right panel corresponds to data point #1, while the one to the left corresponds to data point #2. The graphs at the top show the genomic element densities for the entire population. Chromosomes are vertically aligned. The color-coding reports the differences in density of EGs and recombination hotspots (sliding window analyses of individual genomes, window size is 20 elements). Histograms of the sliding window analyses are shown at the bottom. Initially, all EGs are located on one side of the chromosomes, while recombination hotspots form a cluster in the middle of the non-essential genes (at the other end of the chromosome). At high mutation rates, the EG clustering is maintained (left, movie shows a period of 100,000 generations). At low mutation rates, EG clustering is not maintained and the EGs become distributed in random patterns (right). Both simulations used identical values for all parameters except for R.
(1.26 MB MOV)

Video S2 Evolution of EG clustering. Visualization of the evolution of clustered genome architectures as a function of time in a population representative for the high-R +MAT evolution experiment shown in Figure 8A in Main Text. The top left panel shows a density difference sliding window plot of all genomes (red = only essential genes, blue = only hotspots, green = equal densities of essential genes and hotspots). The bottom two panels show essential gene and hotspot densities in the entire population. The average clustering score in the population (white, SD in red) is indicated in the top right panel (dashed white line = level of clustering in random architectures [107±17]). The simulation starts with a maximally unclustered genome architecture. The inbreeding percentage is 50%. Movie playback speed: 6,000 generations per second (400 generations per frame).
(7.17 MB MOV)
