# Supplementary material for: Evolution of Mutational Robustness in the Yeast Genome: A Link to Essential Genes and Meiotic Recombination Hotspots
Source: PLoS Genet. 2009 Jun 26;5(6):e1000533. doi: 10.1371/journal.pgen.1000533 (PMC2694357; doi:10.1371/journal.pgen.1000533)
Supplement: Text S1 — The computer simulation S. digitalis; supplementary results and discussion; supplementary materials and methods; supplementary figures and tables; S. digitalis simulation settings. (1.26 MB PDF) [file pgen.1000533.s001.pdf]

Evolution of mutational robustness in the yeast genome: A link to essential genes and meiotic recombination hotspots

Philipp J. Keller and Michael Knop
European Molecular Biology Laboratory (EMBL)
Cell Biology and Biophysics Unit
Meyerhofstrasse 1, D-69117 Heidelberg, Germany

Supplementary Information

A) The Computer Simulation *S. digitalis*

1. Overview of the computer simulation
2. The simplified concept of genomic structure in the simulation framework
3. Digitalization of yeast chromosome architectures
4. Mutations and genomic rearrangements in mitosis
5. Computation of crossovers
6. Inbreeding, outbreeding, the mating type locus and mating type switching
7. Determination of the mutational robustness R_{max}
8. The clustering value
9. Evolution of complex architectures under variable in-/outbreeding conditions
10. Survival competition experiments
11. Simulation protocols and sliding window analyses
12. Open and internal simulation parameters
13. Estimating the mutational robustness of asexual populations
14. Computation

B) Supplementary Results and Discussion

1. Nature of the lethality caused by depletion of Msh2 during vegetative growth
2. Essential gene clustering in pericentromeric regions
3. Mitotic versus meiotic mutations

C) Supplementary Materials and Methods

D) Supplementary Figures, Tables and Videos

Suppl. Figure 1: Mutations and selection for reproductive fitness in *S. digitalis*
Suppl. Figure 2: R_{max} of *S. cerevisiae* chromosome IX and random chromosomes
Suppl. Figure 3: The effect of EG clustering on R_{max} at different inbreeding rates
Suppl. Figure 4: Evolution of pericentromeric EG clustering requires a mating type
Suppl. Figure 5: Evolution of EG clustering in *S. cerevisiae* X-like chromosomes
Suppl. Figure 6: Competition analysis of mating type switching populations vs. non-switching populations at different levels of deleterious pre-load

Suppl. Figure 7: Number of crossovers and ORFs in *S. cerevisiae* chromosomes
Suppl. Figure 8: Competition analysis of crossing over rates in yeast chromosomes
Suppl. Figure 9: FACS sorting of spores and dyads
Suppl. Figure 10: Germination efficiency and colony size distribution of FACS-sorted single spores
Suppl. Figure 11: Mating success rates in the presence of mutational load
Suppl. Figure 12: Purging and survival rates in the evolution of clustering experiment

Table 1: Essential gene clustering in *S. cerevisiae*
Table 2: The parameters and simulation modules of *S. digitalis*

Video S1: Maintenance of EG clustering at low and at high mutation rates
Video S2: Evolution of EG clustering

E) *S. digitalis* Simulation Settings

F) References

A) The Computer Simulation *S. digitalis*

1. Overview of the computer simulation

The computer simulation termed *S. digitalis* has been designed to model the basic life cycle of populations of unicellular diploid individuals containing simplified descriptions of their genomes. In this section, a general outline of the simulation is given, including descriptions of all simulation rules. Since the simulation source code is included as supplementary online material, a detailed documentation of all modules (including some optional features not used in the present study) will be provided in the following sections (see Sections A2-A12, Table 2), allowing users to set up their own *in silico* experiments using *S. digitalis*.

The life cycle of the simulation considers diploid individuals that are subjected to alternating rounds of vegetative (mitosis) or sexual (meiosis) divisions. Meiotic progenies immediately return to a diploid life cycle via mating. Mating type loci (*MAT*), of which two opposite types exist (*MATa* and *MAT*α), are optional. If active, mating requires that engaged individuals have opposite *MAT* loci. Mating can occur between individuals from the same meiosis (inbreeding, automixis, intratetrad mating) or between individuals from different meioses (outbreeding, amphimixis).

The framework of the simulation represents each individual by a genome that consists of one diploid chromosome. The basic building blocks of these chromosomes are essential genes (EGs), non-essential genes (NEGs) and intergenic elements (IEs) (see Section A2). IEs are either proficient for meiotic recombination (hotspots) or not (coldspots). Additionally, mating type loci (*MAT*) can be introduced. The position of the *MAT* loci is then either linked to a discrete location in the chromosome, or present externally on another chromosome. If present on another chromosome, the *MAT* is linked to the simulated chromosome via the centromere, which can be simulated at a specified position. If *MAT* loci are enabled, a diploid individual contains one *MAT* locus of each type on each chromosome of the homologous pair at identical positions.

The population of individuals is implemented as a two-dimensional matrix. Each pair of adjacent columns in this matrix defines the structural composition of one specific genome present in one diploid individual in the population (see Figure 3A in Main Text). Each element within a column accounts for one gene or one intergenic element. Genes and intergenic elements alternate. EGs exhibit identity; a viable individual needs at least one functional copy of each essential gene. NEGs do not exhibit identity.

An initial set of genomes (the "seeding genome") is provided at the start of the simulation and generated either by the user or by an integrated random genome generator that constructs genomes according to specifications (number of elements, specific or random distribution of individual elements). In some experiments (see Figure 4D in Main Text), we analyzed the performance of digital yeast chromosomes under mutagenic stress. These chromosomes were modeled according to genome-wide data on crossing over sites published by Mancera *et al.* (2008) [1] (see Section A3). The size of the initial population is adjustable; however, at least one individual must be present at any time during the simulation. If the number of individuals is reduced to zero, the program stops and the population is considered extinct. Genomes are removed from the matrix, if one of the following situations arises: the genome lacks a functional copy of at least one EG, or a genome has been randomly selected for removal ("starvation") in an overpopulated matrix, i.e. in a matrix with a number of columns that is larger than the specified population size cap.

S. digitalis iteratively applies a simulated life cycle to the initial set of genomes (see Figure 3B-D in Main Text). The three main modules of this cycle are mitosis, meiosis and mating (in this order). Mitosis is initiated by a duplication of the genomes in the population matrix, i.e. a copy of each pair of adjacent columns is generated. Subsequently, mutations that lead to the functional inactivation of essential genes at a specified rate R are applied. In addition, structural rearrangements (optionally either swapping of genomic sites, which may either be genes or IEs, or inversions of entire chromosomal fragments within a column) can be applied to the population matrix at random (see Section A4). The structural fitness of each genome is then evaluated. The fitness is defined as "1", if at least one functional copy of each essential gene within a genome is present; otherwise it is defined as "0". Zero-fitness genomes are removed from the matrix. If the total number n of vital genomes is larger than a pre-defined limiting value m (the "population size cap"), n - m genomes are removed from the matrix at random.

Meiosis also starts with a duplication of the matrix's columns, i.e. four haploids are generated from each diploid genome, and subsequently mutations in essential genes can be applied. Unlike in mitosis, each quadruplet of haploids then undergoes meiotic recombination. In the recombination module, fragments are exchanged between the haploids. The locations of these fragments are determined by computation of crossover events between the haploids. A crossover can only occur at hotspots and only between non-sister chromatids. Three different algorithms are implemented for the computation of crossover events in the genome (see Section A5). The distribution of crossover events in the genome is modeled by the mathematical description of "crossover interference" that quantifies the likelihood of finding two crossover events at a certain distance to each other (Figure 3C in Main Text). Mathematically, crossover interference is described by an Erlang probability density distribution [2,3] (F. W. Stahl, personal communication). The total number of resulting crossover events is then statistically defined by this probability density distribution. Each crossover event is fully characterized by its location in the genome, the two participating

haploids and the parity of the resulting exchange of fragments (the decision of exchanging fragments upstream or downstream of the crossover location). The simulation framework also allows the user to switch off crossover interference, but this option was not used in the present work (see Section A5, paragraphs on "Erlang-based computation of random crossover locations" and "Random recombination").

Having determined the structural composition of all haploids on the basis of the calculated crossover events, new diploid genomes are defined by the mating module. Three mating concepts have been implemented: inbreeding, outbreeding and mating type switching (see Section A6). In inbreeding ("intratetrad mating" [4], amphimixis), non-sister or sister chromatids from the same parent individual are combined to form diploid genomes. Depending on the presence of a mating type locus, four (+*MAT*) or six (-*MAT*) different pairings of haploids are possible for each set of four haploids (see Figure 3D in Main Text). In outbreeding, haploids from different parent individuals, i.e. columns from different tetrad-subgroups in the population matrix, are combined. In mating type switching, haploids are selected at random, duplicated and combined with their own copy. The simulation framework allows defining an inbreeding fraction i (the probability of inbreeding used for determining the mating events) as well as (optionally) a mating type switching probability s. In scenarios involving outbreeding ($i < 1$), the outbreeding mating partners are chosen at random from the entire population matrix, but in agreement with the rules of inbreeding and outbreeding (see Section A6). Only haploids of opposite mating types can be combined, if a mating type locus is present. If mating results in a lethal structural constellation (see above) the corresponding pair of columns is removed from the matrix. At the end of each mitotic and meiotic cycle, the population size cap is evaluated and if necessary, random removals of genomes are performed in order to limit the number of genomes to the pre-defined maximum value ("starvation").

The simulated life cycle of the population matrix is iterated until the pre-defined number of cycles has been reached or the population becomes extinct (e.g. due to a high mutation rate R that does not sustain growth).

S. digitalis is able to perform a wide range of experiments. The major applications shown in this study are mutational robustness benchmarks (see Section A7), evolution/maintenance of essential gene clustering (see Section A8), evolution of complex genome architectures under variable inbreeding/outbreeding conditions (see Section A9) and survival competition scenarios (see Section A10). In mutational robustness benchmarks, the maximum mutation rate R_{max} a given genome architecture is able to endure is being determined. In evolution/maintenance of essential gene clustering experiments, the formation and disruption of essential gene clusters is monitored and analyzed (see Section A11), with a focus on the determination of the boundary conditions (properties of meiosis/recombination, breeding strategy and mating type, mutation and EG and hotspot distributions) that provide higher

fitness (competitive advantage) or mutational robustness (maximum value of R a population can withstand) or that allows for the evolution of particular non-random distributions of chromosomal elements. Survival competition experiments simulate the coexistence of two populations with different properties (different architectures or different mating/recombination behaviors) in an environment that sustains a (predefined) maximum total population (sum of the two individual subpopulations). Survival competition experiments allow to determine the population that is best designed for survival in a given environment (i.e. in a specific parameter space).

Most of the modules and parameters of the simulation can be modified by the user. Therefore, we provide an overview table that lists all properties as well as brief instructions on how to operate the program (see Section A12 and Table 2).

2. The simplified concept of genomic structure in the simulation framework

Each of the genomic elements in the population matrix is characterized by an integer identifier according to the following code: Recombination deficient intergenic elements (recombination coldspots) are represented by the value “0”. Recombination proficient intergenic elements (recombination hotspots) are indicated by the value “1”. Intergenic elements are always flanked by two genes. Since genes and intergenic elements alternate in each column, each genome with a total length of $2k+1$ structural components consists of $k+1$ genes and k intergenic elements. Genes are categorized as non-essential and essential genes. Since a deleterious mutation in a non-essential gene has no further effect within the simulation framework, both functional and non-functional non-essential genes are indicated by the same value (“2”) in the matrix. Essential genes, however, have a unique identity and are therefore represented by a unique identifier. Depending on their mutagenic state the identifier starts with a leading “1” (functional essential gene) or a leading “2” (mutated essential gene), followed by three digits that define the gene’s identity.

The structural composition of *S. cerevisiae* chromosome IX shall serve as an example. The digital version of this chromosome consists of 413 elements (n_{genes} = 207 genes and n_{IEs} = 206 intergenic elements). n_{EGs} = 35 of the 207 genes are essential genes and represented by the identifier “10XX” with “XX” ranging from “01” to “35”. The remaining 172 genes are non-essential genes. $n_{hotspots}$ = 58 of the 206 intergenic elements are recombination hotspots. One chromosome IX is represented by the following sequence of identifiers (reformatted into a row):

[2 0 2 0 2 0 2 0 2 0 1035 0 1034 0 2 0 2 0 2 0 2 0 2 0 2 0 2 0 2 0 2 1 2 1 2 1 2 0 2 1 2 1 2 1 2 1 2 1 2 1 2 0 1033 0 2 0 2 0 1032 0 2 0 2 0 1031 0 1030 0 1029 1 2 1 2 1 1028 1 2 1 2 0 1027 1 2 1 2 1 2 1 2 0 2 0 1026 0 2 0 2 0 1025 0 2 0 2 0 2 0 2 0 2 1 2 1 2 1 2 1 2 1 2 1 1024 1 2 1 2 0

2 0 2 0 2 0 1023 0 2 0 2 0 1022 0 2 0 1021 0 2 0 2 0 2 1 2 1 2 1 2 1 2 1 2 1 2 1 2 1 2 1 2 0
1020 0 2 0 2 0 2 0 2 0 2 0 2 0 2 0 1019 0 2 0 2 0 1018 0 2 0 2 0 1017 0 2 0 2 0 2 0 2 0 2 0 2 0
2 0 2 0 2 0 2 0 2 0 2 0 2 1 1016 1 2 1 2 1 2 1 2 1 2 1 2 1 2 1 2 1 2 0 2 0 1015 0 2 1 1014 1 2 1
2 1 2 1 2 1 2 1 2 1 2 1 2 1 2 1 2 0 2 0 2 0 2 0 2 0 2 0 2 0 2 0 2 0 1013 0 2 0 2 0 2 0 1012 0
1011 0 2 0 1010 0 2 0 2 0 2 0 2 0 2 0 2 0 2 0 2 0 2 0 2 0 2 0 2 0 2 1 2 0 1009 0 1008 0 2 0
2 0 2 0 2 0 2 0 2 1 2 0 1007 0 1006 0 2 0 2 0 1005 0 1004 0 1003 0 2 0 2 0 1002 0 2 0 2 0 2 0
2 0 2 0 2 0 2 0 1001 0 2 0 2 0 2 0 2 0 2 0 2 0 2 0 2 0 2 0 2 0 2 0 2]

Since the investigation of the impact of essential gene clustering in regions of low meiotic recombination is a major focus of this work, a module for the automated generation of clustered genome structures (which then function as seeding genomes) was implemented. Using this module, the user may provide the parameters n_{genes}, n_{EGs} and $n_{hotspots}$ as well as a fourth parameter, the number of essential gene clusters $n_{cluster}$ (with $n_{cluster} \leq n_{EGs}$). $n_{cluster}$ defines the total number of recombination hotspot-free genome fragments that are separated by at least one recombination hotspot. The essential genes are evenly distributed amongst these fragments, while the recombination hotspots are evenly distributed amongst the interfragment regions. If $n_{cluster} = n_{EGs}$, any two essential genes in the seeding genome are separated from each other by at least one recombination hotspot. This situation is referred to as a "maximally unclustered genome". If $n_{cluster} = 1$, all essential genes are located in one single large essential gene cluster that contains only recombination coldspots as intergenic elements. This situation is referred to as a "maximally clustered genome". Additionally, there are modules for the generation of randomly structured genomes (based on the layout parameters n_{genes}, n_{EGs} and $n_{hotspots}$) and for the initialization of seeding genomes from a user-provided database.

Finally, since the seeding genome does not have to contain all essential genes in two functional copies, all modules can be combined with a seeding mutation module that randomly mutates essential genes (indicated by the identifier "2XXX") according to a user-defined ratio prior to the start of the simulation.

3. Digitalization of yeast chromosome architectures

Genome-wide information about the position of genes, recombination hotspots and centromeres as well as the categorization of the genes (essential/non-essential character) were obtained from www.yeastgenome.org (for the positions of essential genes), from Gerton *et al*., (2000) [5] (for the positions of recombination hotspots) and from Mancera *et al*. (2008) [1] (for the positions of the break points of crossovers). We used the following algorithm to convert the Mancera *et al*. data into digital chromosomes in the *S. digitalis* framework:

1. For each chromosome, an array of the length ($2n_{genes}$ - 1) was generated as a structural template. The numbers "2" or "1XXX" were assigned to the odd-indexed slots from 1 to ($2n_{genes}$ - 1), depending on whether Mancera *et al*. defined the corresponding gene as being essential or not. As explained in Section A2, "2" represents non-essential genes, while a four-digit code (leading "1" followed by a unique identification number) represents the essential genes.
2. The numbers "0" (coldspot) or "1" (hotspot) were assigned to the even-indexed slots from 2 to ($2n_{genes}$ - 2), depending on whether Mancera *et al*. found at least one recombination event between the genes flanking the respective intergenic slot. It should be noted that by assigning the hotspot-character to each intergenic fragment with at least one detected recombination event, we are likely to underestimate the level of essential gene clustering in the real chromosome. Despite this conservative approach we find a significantly better performance of the digital yeast chromosomes if compared to random architectures in a survival competition assay.
3. The index of the gene or intergenic element closest to the measured centromere position was defined as the centromere position in the respective digital chromosome.

An analogous algorithm was applied to the data from Gerton *et al*. (2000) [5], which we initially used for digitalization of chromosome IX (at this time point of the work the data by Mancera *et al*. (2008) [1] was not yet available).

4. Mutations and genomic rearrangements in mitosis

Deleterious mutations and structural arrangements occur at random at the end of mitosis. Alternatively, mutations may also be activated after the duplication step in meiosis, or optionally both in mitosis and meiosis. The mutation rate R defines the average number of essential gene inactivating mutations per mitosis and per genome (or per life cycle and per genome, if the meiotic module is active as well). The statistical probability defined by R is assumed to be identical for all genes. Intergenic elements cannot mutate. Optionally, the simulation framework allows for an adaptive mutation rate (a feature that was not studied in this work). In this scenario, the mutation rate is continuously adjusted during simulation run-time, based on an evaluation of the population size and the speed of population growth. The framework subjects the population to the maximum mutagenic stress that still allows for the population's survival. In other words, the mutation rate is increased if the population grows rapidly, while it is decreased if the population is in danger of becoming extinct. While this type of experiment in principle allows for the determination of the mutational robustness R_{max} of genome architectures, we employed a different and more robust approach for this purpose (see Section A7).

Similarly, the genomic rearrangement rate *r* defines the probability of a restructuring event per genomic element and per mitosis. Rearrangements can be applied in two different ways, either by site swapping or fragment inversions. Both mechanisms are related, since the effect of a site swapping can also be achieved by a pair of fragment inversions (see below). The simulation framework provides a switch that allows the user to assign the active module. For this work, we restricted the simulation to the application of the swapping module.

If a swapping event is applied to a genomic element e_1 (either a gene or an intergenic element), a random swapping target e_2 is determined within the same column of the population matrix (i.e. on the same chromatid). The algorithm then determines the homologous site e_1' on the second chromatid of the same chromosome. If e_1 is an essential gene, the algorithm defines the genomic element with the same identity on the second homologue as e_1'. In any other case (identifiers "0", "1" or "2") the geometrically closest region with the same identifier is assigned as the homologous region e_1'. e_2' is determined in the same way. In the last step, the algorithm swaps the identifiers at the positions e_1 and e_1' as well as at e_2 and e_2'.

Fragment inversions are performed similarly. Since genomic rearrangements can lead to a decrease in the level of homology of the two strands of a chromosome in outbreeding situations (but not in inbreeding situations), fragment inversions are only allowed in inbreeding experiments (see below). If an inversion event is assigned to a genomic element e_1, a second element e_2 is determined in the same column of the population matrix in order to mark the end point of the inversion. The homologous elements e_1', e_2' are then determined as described above and the sections [e_1 e_2] on the first homologue and [e_1' e_2'] on the second are inverted.

Additional rules apply for outbreeding in the presence of genomic rearrangements (a scenario that was not applied in this work). We observed amplification of EGs as a consequence of outbreeding in populations containing individuals with different chromosome architectures. To prevent or restrict this phenomenon additional parameters have to be provided, including the required number of NEGs and the reproductive barrier (the level of homology that chromosomes require in order to allow a faithful meiosis I). While such parameters can in principle be specified in *S. digitalis*, we did not activate these modules in the present work.

Mitotic rearrangements can also influence the position of the mating type locus. If the genomic location of the mating type locus is linked to a site that becomes subject of a swapping or inversion event, the mating type locus remains linked to this site and is therefore also repositioned.

5. Computation of crossovers

Computation of crossover events on the basis of crossover interference

The computation of crossover events on the basis of crossover interference is facilitated via an Erlang probability density distribution (see Figure 3C in Main Text). This mathematical model quantifies the probability p of measuring a distance d between two crossovers. The distance d between the two crossovers can be defined as the number of intermediate recombination hotspots (genetic distance definition) or as the total number of intermediate genetic elements (physical distance definition).

The Erlang distribution with the shape factor k is defined as follows:

$$p_k(d) = 2k \exp\{-2kd\} \frac{(2kd)^{k-1}}{(k-1)!} \tag{1}$$

A shape factor k = 4 describes crossover interference in *S. cerevisiae* best (information kindly provided by Frank Stahl). This results in the probability distribution:

$$p_4(d) = \frac{2048}{3} d^3 \exp\{-8d\} \tag{2}$$

An optional scaling factor s was implemented in the simulation. This factor allows adjusting the average number of crossover events per genome, a degree of freedom that was investigated in the analyses shown in Figure 10 in Main Text. Depending on the distance definition, the discrete distance unit u is normalized by $n_{max} = n_{genes}$ (physical definition) or $n_{max} = n_{hotspots}$ (genetic definition).

$$u = \frac{s}{n_{\max}} \tag{3}$$

In the simplified framework of the simulation, each recombination hotspot contributes equally to the computation of the distance d, i.e. all hotpots are assumed to be characterized by the same quantitative intrinsic ability of facilitating double strand breaks. In order to compute a probability distribution q_1 for the discrete distances of the simulation framework, the probability density distribution must be stepwise integrated.

$$q_1(n) = \int_{(n-1)\cdot u}^{n\cdot u} p_4(x)dx \text{ with } 1 \le n \le (n_{\max} - 1) \tag{4}$$

The boundary condition arises from the maximal possible distance:

$$q_1(n_{\max}) \equiv \int_{n_{\max}\cdot u}^{\infty} p_4(x)dx \tag{5}$$

A statistical estimate c of the average number of crossover events per genome can be directly computed from q_1:

$$c = \frac{n_{\max}}{\sum_{n=1}^{n_{\max}} n \cdot q_1(n)} \tag{6}$$

The integration over q_1 results in a probability distribution q_2 that returns the probability for observing the next crossover event within n discrete distance units.

$$q_2(n) = \sum_{k=1}^{n} q_1(k) \tag{7}$$

The occurrence of recombination is a statistical phenomenon in the simulation, i.e. in addition to activating crossover interference the user can define a probability per genome for the initiation of the computation of crossover events. The computation of a complete series of crossovers for a given tetrad starts with localizing all hotspots within the four haploids. One of these hotspots is chosen at random for the first crossover event. The probability distribution q_2 is then iteratively employed to determine the location of neighboring events (both up- and downstream of the initial event). If a recombination hotspot is present on all four haploids (as compared to only two haploids) at a specific genomic location and the distance definition mode is set to genetic distance, the probability of a crossover event at that position is doubled. The computation of crossover events is aborted, if the next target region is located outside the population matrix.

In the last step, the identity of the involved haploids is randomly determined for each crossover event. Four configurations are possible. For a tetrad A A' B B', which is created by duplication of the genome A B, the crossover constellations AB, AB', A'B, A'B' are

allowed. Crossovers cannot occur between sister chromatids. Having computed the entire set of crossovers and the corresponding haploid configurations, the algorithm sequentially evaluates the events (starting at one end of the chromatid). A random flag determines whether the first chromosomal fragment, i.e. the fragment that stretches from the chromatids' end to the first active recombination hotspot, is recombined or not. Subsequent fragments are then recombined in an alternating fashion. The mating types of haploids are swapped if the mating type locus is located on a chromosomal fragment that is affected by meiotic recombination.

Computation of random crossover locations in the absence of crossover interference

The simulation allows investigating the effect of crossover interference. We did not use this module in the current work, but, since it is provided in the code, a brief description of its implementation shall be given:

In order to achieve identical levels of crossover as compared to the crossover interference situation, the total number of crossover is first computed using the Erlang-based computation of crossovers. The actual positions of crossovers are discarded and replaced by locations that are computed from a uniform probability distribution within the set of coordinates provided by the recombination hotspots. This approach ensures that in the statistical average the number of crossovers is identical to a situation, in which crossover interference is active. The locations of the crossovers, however, are not affected by the Erlang distribution.

Random recombination with fixed crossover frequencies

In this scenario, the number of crossover events is not subjected to statistical fluctuations, but rather given by the statistical average c defined in equation 6. The locations of the crossover events are determined by a uniform probability distribution. This module was not employed in the experiments presented in this study.

6. Inbreeding, outbreeding, the mating type locus and mating type switching

Starting with n genomes at the onset of meiosis, the meiotic recombination module will provide $4n$ restructured haploids that subsequently undergo the mating procedure. The haploid $h_{p,q}$ (with $p \in [1;n], q \in [1;4]$) originates from the paternal genome p and is distinguished from the other haploids of the same tetrad by the identifier q. The simulation interface allows defining an arbitrary inbreeding/outbreeding-ratio i (with $0 \le i \le 1$). Thus, taking into account the optional presence of a mating type locus, four different mating situations can occur. The mating type locus introduces an additional parameter α that is either assigned as "-1" or "1", depending on the mating type of the haploid. The following rules apply to the six identifiers a, b, c, d, α and β of the two mating haploids $h_{a,b}$ (with mating type α) and $h_{c,d}$ (with mating type β):

1. inbreeding without a mating type locus: $a = b$; α and β are undefined.
2. inbreeding with a mating type locus: $a = b$; choice of c and d must result in $\alpha{\cdot}\beta = -1$.
3. outbreeding without a mating type locus: $a \neq b$; α and β are undefined.
4. outbreeding with a mating type locus: $a \neq b$; choice of c and d must result in $\alpha{\cdot}\beta = -1$.

Inbreeding and outbreeding events are computed in agreement with these four rules, but at random with respect to the existing degrees of freedom.

Optionally, a mating type switching probability s may be defined. If $s > 0$, an according number of haploids are picked at random and subjected to a module for mating type switching. These haploids do not participate in inbreeding or outbreeding. Instead, their architectures are duplicated and the haploids are combined with their own copy. Since this step will inevitably lead to the homozygotisation of any essential gene mutation, an individual with a diploid genome that has been created by mating type switching can only survive, if none of its essential genes are mutated.

The mating type can be exchanged between haploids by means of crossovers during meiosis and can be the target of structural rearrangements during mitosis. The genome duplications that occur at the onset of mitosis and meiosis transfer the identity of the mating type to the new chromatids.

The simulation framework provides the option to define an "external" mating type locus position. This option considers a scenario, in which the simulated chromosome is linked to the mating type located on another (non-simulated) chromosome in the same cell. Mathematically, this situation corresponds to a 50% probability per meiosis for an inversion of the mating type of the four haploids in a tetrad.

7. Determination of the mutational robustness R_{max}

The simulation interface provides the option to screen for the population survival boundary with respect to the mutation rate R. This parameter, the mutational robustness $R_{\max}$, is defined as the average mutation rate at which the transition from population survival to population extinction occurs. In other words, R_{max} is the largest average mutation rate at which the simulated population still survives for a predefined number of life cycles. If R_{max} can be approximated from previous investigations, the computation time for R_{max} can be significantly reduced by specifying the desired resolution R_{res} and an interval of mutation rates $\left[R_0; R_1\right]$ that includes the mutational robustness. The simulation algorithm then starts the first simulation run at the specified maximum mutation rate R_1 and iterates the experiment

with reduced mutation rates (using the step size R_{res}) until the first successful experiment is completed, i.e. until a population survives the predefined number of simulation cycles. The simulation returns the mutation rate that corresponds to this run.

Since R_{max} is subject to statistical fluctuations, the user can specify the level of averaging a performed in the calculation. If $a > 1$, the procedure of computing R_{max} is repeated $(a - 1)$ times and an array of the resulting mutation rate survival boundaries as well as the average mutation rate survival boundary $R_{max,a}$ and its standard deviation are returned.

8. The clustering score

The investigation of clustering of essential genes in genomic regions is a major focus of the simulation. Therefore, a parameter has been introduced that quantifies the level of clustering and allows comparing different structural constellations. This parameter is termed “clustering score” v. The purpose of the clustering score is the rating of genomes, providing a low score for unclustered genomes and a high score for strongly clustered ones. In a maximally unclustered genome, any pair of essential genes on a chromosome is separated by at least one recombination hotspot. This structure scores the lowest v-value. The highest level of clustering is reflected by a genome with one large essential gene cluster, i.e. a continuous fragment that contains all essential genes but no recombination hotspots as intergenic elements. This situation scores the highest v-value.

The clustering score analysis of a genome is performed as follows. The algorithm detects all positions e_i (i = 1..n) in the genome that at least one of the genome's two homologues has a recombination hotspot at. Additionally, the positions e_0 and e_{n+1} are defined as the two end coordinates of the genome. In the second step, the algorithm computes the numbers s_i (i = 1..(n + 1)) of essential genes that are located in each fragment $\left[e_{i-1};e_i\right]$. The clustering score v is then defined as:

$$v = \frac{n_{hotspots} + 1}{n_{EGs}} \sum_{i=1}^{n+1} s_i^2 \qquad (8)$$

The clustering score is normalized by the total number of essential genes n_{EGs} and the total number of hotspot-separated structural fragments $n_{hotspots} + 1$ in the genome as indicated in equation 8. The normalization factor allows comparing the level of essential gene clustering in different genome layouts.

The seeding genome of the "creation of clustering in small genomes" experiment shall serve as an example (see Figures 7C/D in Main Text). The genome layout is characterized by n_{EGs} = 5 essential genes and a total of n_{genes} = 10 genes and $n_{hotspots}$ = 4 recombination hotspots.

The maximally unclustered structure of the seeding genome (each pair of essential genes is separated by a recombination hotspot) is defined as follows:

[1001 0 2 1 1002 0 2 1 1003 0 2 1 1004 0 2 1 1005 0 2]

[1001 0 2 1 1002 0 2 1 1003 0 2 1 1004 0 2 1 1005 0 2]

The two homologues of the genome are identical and reformatted in a row representation. The scoring algorithm detects 5 clusters of essential genes (n = 4) with a size of one unique essential gene identity each (s_i = 1 for i = 1..5). Therefore the clustering score v results as $v = ((1 + 4) / 5) \cdot (1^2 + 1^2 + 1^2 + 1^2 + 1^2) = 5$.

Using the same layout parameters, we may define a maximally clustered genome as follows:

[1001 0 1002 0 1003 0 1004 0 1005 0 2 1 2 1 2 1 2 1 2]

[1001 0 1002 0 1003 0 1004 0 1005 0 2 1 2 1 2 1 2 1 2]

All essential genes are located in one large cluster. The clustering value v for this structure results as $v = ((1 + 4) / 5) \cdot (5^2 + 0^2 + 0^2 + 0^2 + 0^2) = 25$. In this very simple structural layout with five essential genes, the following clustering scores are possible: 5, 7, 9, 11, 13, 17 and 25. A larger clustering value indicates a higher level of essential gene clustering in the analyzed structure.

Due to the nature of its definition, the clustering score is particularly sensitive to large clusters. Some non-random distributions may yield relatively low scores, if they are accompanied by an unusually large number of single EGs that are flanked by hotspots. While this can be seen as the main disadvantage of the "clustering score", it should also be noted that this parameter is particularly robust, analytically accessible and suitable for comparing chromosomes of different lengths, due to the normalization on total hotspot and essential gene numbers. These were our main reasons to use this score. For a discussion of an alternative sore, the "grouping score", see Section A11 ("Simulation protocols and sliding window analyses").

9. Evolution of complex architectures under variable inbreeding/outbreeding conditions

This simulation module allows defining initial populations with arbitrary sizes and arbitrary levels of complexity. The aspect of complexity arises from the option of categorizing genomes with different architectures in sub-populations within the main population. Only individuals within the same sub-population can be partners in outbreeding. Arbitrary mutation rates, genome rearrangement rates and inbreeding/outbreeding ratios can be applied during the life cycle. The simulation framework keeps track of the modification of individuals by stochastic rearrangements and defines new sub-populations upon changes in the architecture that lead to repositioning of essential genes, recombination hotspots and coldspots and mating type loci. This dynamic grouping of the population matrix into sub-populations allows applying a global user-defined inbreeding/outbreeding-ratio without the need to consider a potential architectural incompatibility of outbreeding individuals. For all aspects of the life cycle other than mating (e.g. nutritional supply, stochastic mutations), the population matrix is considered as one entity irrespective of the sub-population groupings and all individuals are therefore subjected to the same conditions. As a consequence, this simulation module is particularly useful for the evolution and monitoring of large genome architectures under variable inbreeding/outbreeding conditions.

This aspect of the simulation has been implemented to facilitate the experimental analysis of the evolution of *MAT*-linked and peripheral essential gene clustering in large populations with yeast chromosome IX-like genome content (see Figure 8A in Main Text). Initially, the 35 essential genes, 172 non-essential genes, 58 recombination hotspots and 148 recombination coldspots of the chromosome IX-like genomes were arranged such that a genome architecture with a minimal level of clustering resulted. In this architecture, each pair of essential genes was separated by at least one recombination hotspot (clustering score = $n_{hotspots} + 1 = 59$). The evolution of the architectures in the initial seeding populations was then monitored over a time period of 100,000 generations with active/inactive mating types and at low and high mutation rates (see Video S2).

10. Survival competition experiments

Some of the results shown in this paper are based on survival competition experiments, in which two populations with different initial genomes compete for the same pool of nutrients. Nutrient limitation is mimicked by a maximum population size that applies to the sum of individuals from both sub-populations. Binary identifiers map the individuals in the initial population matrix to the sub-populations. The simulation then monitors and analyzes the evolution time-course of both populations. Mating occurs strictly within the sub-populations, but both populations together are subjected to the population size cap, i.e. if the size of one population stagnates but the other population grows rapidly, the stagnating population will also be affected by starvation. The populations can be subjected to different reproduction

mechanisms (vegetative/sexual) or different recombination rates. The mating types in the two populations can be positioned differently (e.g. externally in one population and internally in the other, or simply in different regions of the chromosomes).

Each competition experiment starts with the same number of individuals in both populations. There are four possible outcomes of the simulation run, which lasts either until all individuals of (at least) one of the sub-populations are extinct (cases 1, 2 and 4) or until a specified maximum number of generations is reached (case 3):

1. Population A prevails, i.e. population B is extinct and at least one individual of population A is still alive.
2. Population B prevails, i.e. population A is extinct and at least one individual of population B is still alive.
3. Both populations survive a predefined number of simulation cycles (generations).
4. Both populations simultaneously become extinct before a predefined number of simulation cycles is reached.

This competition scenario is a simple and straightforward way of comparing the mutational robustness provided by different genome architectures, mating behavior or recombination frequencies.

11. Simulation protocols and sliding window analyses

At simulation run-time, protocols are generated that provide an easy access to pre-processed data, which are directly obtained from the population matrix. In order to save disk space, the population matrix itself is stored on the hard disk only every one-hundredth generation. Using the dynamic protocols, every single event that occurred within the simulation framework and thus also the population matrix at any given time point can be reconstructed. The core protocols monitor the position of recombination hotspots in the matrix, the size of essential gene clusters, the “starvation” of individuals (i.e. the removal of genomes induced by the population size cap), the crossovers during meiotic recombination, mitotic rearrangements, mitotic mutations and the computation of mating partners.

Other protocols monitor the population size after mitosis and after meiosis, the average number of mutations in the population, the average number of functional deleterious mutations (affecting essential genes), the number of genome removals due to homozygous mutations during mitosis and during meiosis, the average number of rearrangements, the average number of recombination events, the number of attempted and successful inbreeding

and outbreeding events, the number of genome removals due to a lack of homology or due to a lack of recombination hotspots (only for outbreeding experiments in combination with a non-zero rearrangement rate), the amount of mutagenic load in the population during mitosis and meiosis and the amount of mutagenic load in deleted genomes during mitosis and meiosis (mutagenic purging).

Finally, two types of structural analyses are performed at simulation run-time: the determination of the average population clustering score (see Section A8) and a sliding-window histogram analysis of the genetic contents of the population matrix.

In the sliding-window analysis, each column in the population matrix is analyzed with respect to a local presence of essential genes and recombination hotspots. A sliding window filter, typically of the size n_{window} = 10 genetic units, is moved unit-wise from the top to the bottom of each column, while the total number of essential genes and hotspots is noted in two protocol arrays. In the histogram analysis the frequencies of the sliding window counts are determined. The "grouping score" g is defined as the standard deviation of the array that results by subtracting the sliding-window array for essential genes from the array for recombination hotspots. g is another measure for essential gene clustering in the population matrix. We found both the grouping score g and the clustering score v to be useful in the analysis of structural phenomena in the simulation. However, the clustering score v (in contrast to the grouping score g) gives access to a simple analytical assessment of the simulation's results (see e.g. Figure 7B-D in Main Text). Therefore, the results derived in this study are based on the clustering score v.

All protocols and analyses described in this section are stored in individual arrays in the sub-directories "clustering_plots" and "protocol_plots". Graphical visualizations in plots and histograms are also generated and provided as JPEG images. A movie of the evolution of inbreeding populations is provided as Video S1.

12. Open and internal simulation parameters

Most of the simulation's parameters are assigned at the command line when invoking the simulation program. An overview of all open and non-open parameters is provided in a table (see Table 2). The table contains the identifiers used in the simulation code, a brief description of the parameters, the valid numerical ranges and experimental values that are known from literature (if applicable).

13. Estimating the mutational robustness of asexual populations

A deactivation of meiosis in the simulation framework leads to a situation, in which the deleterious mutagenic load k of the genomes monotonously increases until the saturation level $k = n_{EGs}$ is reached (there are 2 n_{EGs} essential gene copies in the diploid genome). At this stage, any additional deleterious mutation will inevitably lead to the death of the concerned individual. Since recombination of the genomes is not possible, neither structural rearrangements nor mating types or recombination hotspots have any effect on the handling of deleterious mutational load. The mutational robustness of the population depends on the number of essential gene copies per diploid genome (2 n_{EGs}) and the size s of the population (expressed as the number of diploid genomes). We define m as the mutation rate per essential gene copy and per mitosis, i.e. $m = R / (2\ n_{EGs})$. The probability $p_{survival}(m)$ for an equilibrated genome (a genome that reached the saturation level of deleterious mutations) to not experience a mutation in one of the remaining functional essential gene copies at a mutation rate m is then

$$p_{survival}\left(m\right) = \left(1-m\right)^{n_{EGs}} . \tag{9}$$

Thus, $p_{survival}$ is the probability of survival for an equilibrated genome. In a population of s individuals, the probability $p_s(m,n)$ that precisely n genomes survive the random application of deleterious mutations is therefore:

$$p_s\left(m,n\right) = \binom{s}{n}\left(p_{survival}\left(m\right)\right)^n \left(1-p_{survival}\left(m\right)\right)^{s-n} \tag{10}$$

An estimate for the mutational robustness R_{max} in the population can be obtained from the mutation rate $m_r = R_{max} / (2\ n_{EGs})$, for which the first momentum of the probability distribution p_s with respect to n takes the value of s / 2:

$$\sum_{n=1}^{s} n p_s\left(m_r, n\right) = \frac{s}{2} \tag{11}$$

At this value of m in average half of the population size s is removed due to homozygous deleterious mutations. Since each mitotic cycle duplicates the population, the population is exactly at the edge of survival. The population does on average neither shrink nor grow.

The mutational robustness of an asexual population is independent of the population size *s* (a result that can also be directly derived from equation 9). Inserting equations 9 and 10 in equation 11 yields:

$$\begin{aligned}
& \sum_{n=1}^{s} n \binom{s}{n} \left(p_{survival}(m_r) \right)^n \left(1 - p_{survival}(m_r) \right)^{s-n} = \frac{s}{2} \\
\Rightarrow \quad & \sum_{n=1}^{s} \frac{n}{s} \frac{s!}{n!(s-n)!} \left(p_{survival}(m_r) \right)^n \left(1 - p_{survival}(m_r) \right)^{s-n} = \frac{1}{2} \\
\Rightarrow \quad & p_{survival}(m_r) \left(\sum_{n=0}^{s} \binom{s}{n} \left(p_{survival}(m_r) \right)^n \left(1 - p_{survival}(m_r) \right)^{s-n} \right) = \frac{1}{2} \\
\Rightarrow \quad & p_{survival}(m_r) = \frac{1}{2}
\end{aligned} \tag{12}$$

We obtain the formula for the mutational robustness R_{max} of an asexual population:

$$R_{\max} = 2n_{EGs} \cdot m_r = 2n_{EGs} \left(1 - \left(\frac{1}{2} \right)^{\frac{1}{n_{EGs}}} \right) \tag{13}$$

14. Computation

S. digitalis was developed in Matlab (v7.4, The Mathworks). Using the Matlab Compiler, the Matlab code was compiled for Unix platforms and executed on computer clusters at the European Molecular Biology Laboratory (EMBL) and at the Karlsruhe Institute of Technology (KIT).

B) Supplementary Results and Discussion

1. Nature of the lethality caused by depletion of Msh2 during vegetative growth

In order to analyze the deleterious mutations that accumulate during vegetative growth in cells depleted for Msh2, we used cells that have been grown on YPD three times for 10-12 generations using serial transfer in order to allow for accumulation of mutations. Thereafter, 2 x 10^7 cells were grown for approximately 1 day on YP-Gal/Raf plates, in order to induce the GalS-promoter followed by sporulation and tetrad dissection. Under these specific conditions we found that ascus formation occurred with a frequency of > 99% within a period of 36 hours. 400 tetrads were dissected and spore viability was scored. Replica plating onto YPD containing G418 or ClonNat was used to investigate the segregation of the two GalS-MSH2 loci, one marked with kanMX (which confers resistance to G418) and the other one with natNT2 (which confers resistance to ClonNat) and on SC-LEU medium to follow the segregation of the *leu2* allele. All tetrads that produced two viable spores were scored, and the linkage of the lethal phenotype was analyzed using the formula cM = 100/2(T/(PD+NPD+T)) [6]. The segregation of lethality in 131 tetrads with two viable spores PD:NPD:T=27:34:70 (versus *MSH2,* marked with kanMX and natNT2) and PD:NPD:T=21:18:92 (for *leu2*). T stands for tetratype tetrads. In our case we scored situations, in which the two viable spores had a different allele with respect to the investigated marker (*leu2*/*LEU2* and *MSH2-kanMX*/*MSH2-natNT2*). PD and NPD tetrads were scored when both viable spores had the same marker. In the case of *GalS-MSH2* (which is not centromere-linked), no linkage to the load was measured (35 cM, a linkage ≥ 35 cM cannot be calculated using this formula), whereas the lethal load scored a linkage of 27 cM to *leu2/LEU2*, indicating that some lethal mutations exhibited centromere linkage. Since spore lethality must be caused by a different mutation in each tetrad with two viable spores, the global linkage of 27 cM of the load to *leu2* represents an average. This average is composed of mutations in essential genes, which may themselves exhibit centromere linkage. Additionally, some linkage may be caused by meiosis I non-disjunction of a chromosome, which results in two (or zero, in the case of several non-disjoined chromosomes) viable spores.

In order to mate, a spore needs to be able to break open the spore wall and show some indication of germination. Microscopic inspection of single dead spores that failed to form visible colonies revealed that their majority (93.5%; n=200) was able to germinate. In most cases they formed micro-colonies of up to approximately 20 cells (the histogram of the observed colony sizes is shown in Supplementary Figure 10). This is likely due to maternal contribution of functional mRNA or protein. We moreover found that most of the spores that did not show signs of germination appeared nevertheless swollen and we could often see faint remnants of what may have been a spore wall (after touching the spore with the needle of the micromanipulator). This indicates some metabolic activity. But it is unclear whether those

spores would still be able to mate. Together, these results provides additional support for single mutations as the cause of the lethal phenotypes, since spores that lack entire chromosomes (either due to meiosis I non-disjunction or meiosis II chromosome miss-segregation) usually fail to germinate. For methods, see Section C below.

For successful mating, spores furthermore require a functioning complement of the proteins required for mating. In order to estimate the frequency of mutations that inactivate the mating machinery, we tested the viable spore colonies for pheromone secretion and mating. We found that 1.2% (5 out of 425) viable spore colonies were deficient in mating, four of which also failed to secrete pheromone. One spore colony was well secreting mating pheromone but completely mating deficient. One spore colony could be induced to undergo haploid meiosis, which could be due to an extra chromosome III. For the remaining three spore colonies we could not easily determine the defect that hindered them from mating.

These tests provide additional support for our claim that mutagenized yeast genomes accumulate mutations that, even though they may be lethal, do not prevent the transmission of genomes through meiosis in the vast majority of the cases, provided that the spores have access to a mating partner immediately after germination.

2. Essential gene clustering in pericentromeric regions

Our simulations predict a fitness advantage for a linkage of essential gene clusters to centromeres. While clustering can be caused by the absence of meiotic recombination in pericentromeric regions, an enrichment of essential genes near centromeres (our previous finding [7]) constitutes evidence for a force underlying chromosome organization that arises from the mutational robustness. In Taxis *et al.* (2005) [7], we have calculated the essential gene enrichment in a region of 10 kb on either side of the centromeres, which corresponds to the physical distance that shows significant centromere linkage (within 35 cM). The correlation between physical distance p (in kbp) and genetic distance g (in cM) in pericentromeric regions (up to approximately 40 cM) can be approximated using the formula:

$$g = -\frac{1}{0.228}\ln\left\{2\cdot\frac{42.2}{p+42.2}-1\right\} \tag{14}$$

The formula was derived from a compilation of the genetic and physical distances present in the yeast genome available at www.yeastgenome.org. Batada and Hurst [8] reported that the essential gene enrichment for this interval is not (or borderline) significant (P = 0.077), while we found it to be significant (P = 0.03) [7]. The deviation arises from the different statistical tests employed in both studies. Batada and Hurst used the non-parametric Mann-Whitney U

test to decide whether the two distributions are significantly different. Non-parametric tests have less statistical power than the corresponding exact tests, which conversely can only be used if the correct theoretical distribution is known. In our case, we are comparing independent counts in two samples, rendering the theoretical distribution hypergeometric, and thus employ Fisher's exact test. In conclusion, Batada and Hurst arrive at a *P*-value of 0.077 whereas we obtain the *P*-value 0.03 for the same data.

3. Mitotic versus meiotic mutations

For most *in silico* experiments we implemented mutations only in mitosis. With respect to all conclusions drawn from our simulation in the present work, this is qualitatively and quantitatively identical to the situation, in which mutations would occur during pre-meiotic DNA replication. This is demonstrated in a series of comparative and competitive benchmarks (Supplementary Figure 2A-D). However, our simplified simulation framework does not consider the possibility of other meiotic mutations (e.g. during meiotic recombination) that may have a quantitative or qualitative impact on the simulation readout. This possibility is disregarded, because there is currently no data available that reports on this specific class of mutations.

C) Supplementary Materials and Methods

Yeast mutator experiment

Haploid yeast strains NKY289 (*MATa lys2 ura3 ho::hisG*) and NKY292 (*MATα lys2 ura3 leu2::hisG ho::LYS2)* [9] in the well-sporulating SK1 background were used. The endogenous *MSH2*-promoter was substituted using PCR targeting and plasmids pYM-N30 and pYM-N31 as templates for PCR as described [10]. The correct integration of the PCR product was validated using PCR. Competent cells and selection of transformants were done using YP-medium containing 2% galactose and 2% raffinose (YP-gal/raff). Upon mating the spore viability of diploids was tested using tetrad dissection and was found to be identical to the wild type strains. For sporulation cells were grown for 24 h on YP-gal/raff plates at 30°C followed by sporulation on plates containing 1% KAc and each 0.02% of raffinose and galactose at 23°C for 36 – 48 hours. For sporulation of cells during a mutation accumulation experiment, 0.2 ml of cells grown in liquid YPD culture ($2x10^7$ cells) were plated on a YP-gal/raff plate, grown for 24 h, washed off with sterile water and plated on SPO plates. Random spores were prepared using washed-off cells in water and Zymolyase 100T (0.2 mg/ml, Seikagoku) and Sulfatase (10%, Sigma) for 1 hour, followed by vigorous vortexing of the cells with acid-cleaned glass beads (1 volume beads, 1 volume cells) for 6 min in total. Spores were then washed two times with 100 mM sodiumcitrate (pH 5.8) containing 1% Triton X100 and once with water. This procedure disrupted all non-sporulated cells and all asci, and also disrupted most of the interspore bridges that were reported to keep pairs of spores together [11]. Most of the dyads observed in the FACS formed after the Triton X-100 washes upon dilution of the spores in the buffer used for FACS, but a few remaining dyad pairs linked by interspore bridges may not have been disrupted. Interspore bridges are likely to keep spores together, which involve genomes separated during meiosis I. This explains why the rate of diploid formation resulted as 53% in sorted dyads in the contest of the *MAT*-centromere linkage and not exactly as 50% in both the wild type strain and at time point 0 h of the *GalS-MSH2* strain.

Germination of FACS-sorted spores was investigated following growth on YPD plates for three days by looking at the individual spores using a tetrad dissection microscope (Singer Instruments). Successful germination was scored when a spore had formed an extension (which may be the shape of a bud or more tube-like) of at least one spore diameter in size. In most cases spores formed micro-colonies that consisted frequently of cells of aberrant shape. A spore was considered to be “dead” if it failed to form a colony visible by eye. Counting or approximate estimation of the number of cells per colony was used to classify the lethal phenotypes. The experiment was conducted with two different clones of the *GalS-MSH2* strain, which both yielded identical results.

For mating testing, two assays were used. Pheromone secretion was tested using a halo-assay and by using pheromone-sensitive strains. Mating was tested using complementation of rare auxotrophic mutations.

D) Supplementary Figures, Tables and Videos

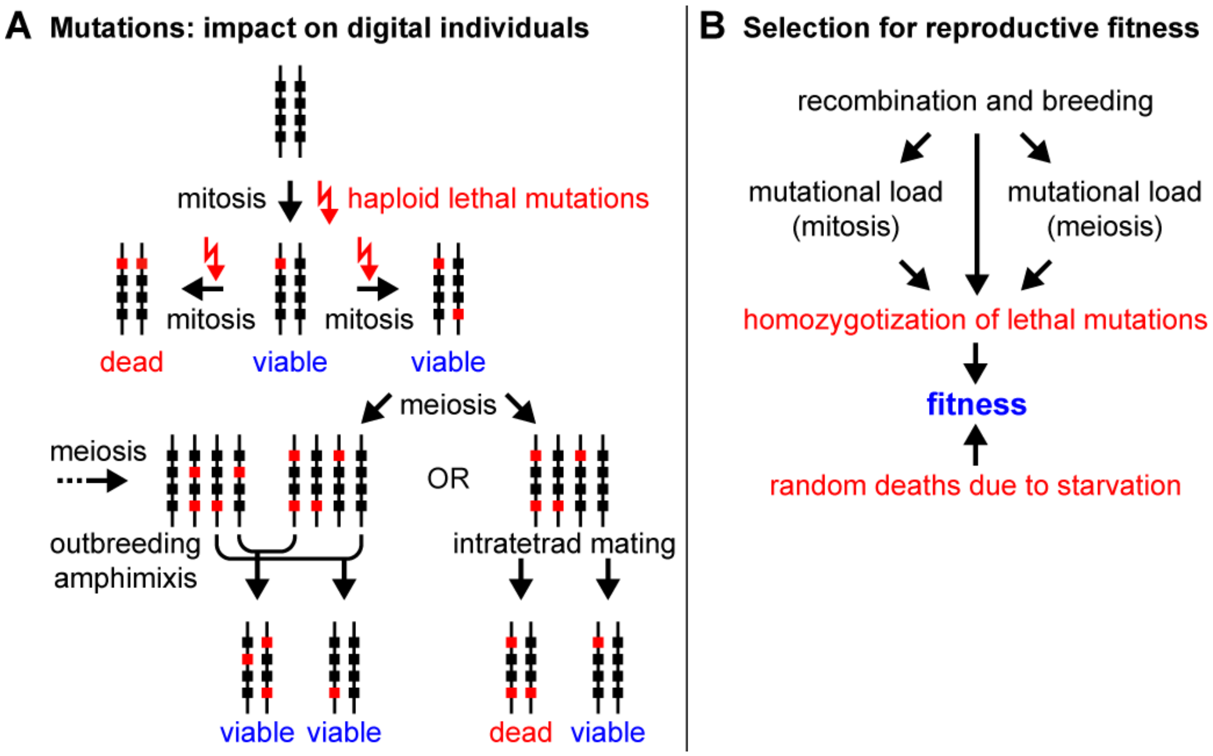

Supplementary Figure 1: Mutations and selection for reproductive fitness in *S. digitalis*

(**A**) Mutations that inactivate essential genes occur at random in mitosis. Mutation of both alleles of an essential gene leads to the death of the individual. Surviving individuals (that carry at least one functional copy of each essential gene) are subjected to meiotic recombination followed by mating of the meiotic products (spores) with other spores. Mating can occur within the tetrad (intratetrad mating/inbreeding) or among spores from different tetrads (outbreeding/amphimixis). The chromatids involved in mating carry a specific load of lethal mutations. Only homozygotisation of mutated essential genes leads to death of an individual.

(**B**) Two mechanisms lead to the death of individual in the simulation: a random removal of individuals due to limitations in the nutritional supply (starvation) and a selective removal of individuals with homozygous mutations in essential genes. The parameters that govern the recombination of genomes in meiosis and mating (chromosome architecture, inbreeding and outbreeding, crossover frequency) affect the mutational load and its distribution across the genome and thereby the frequency of homozygotisation of haploid lethal mutations.

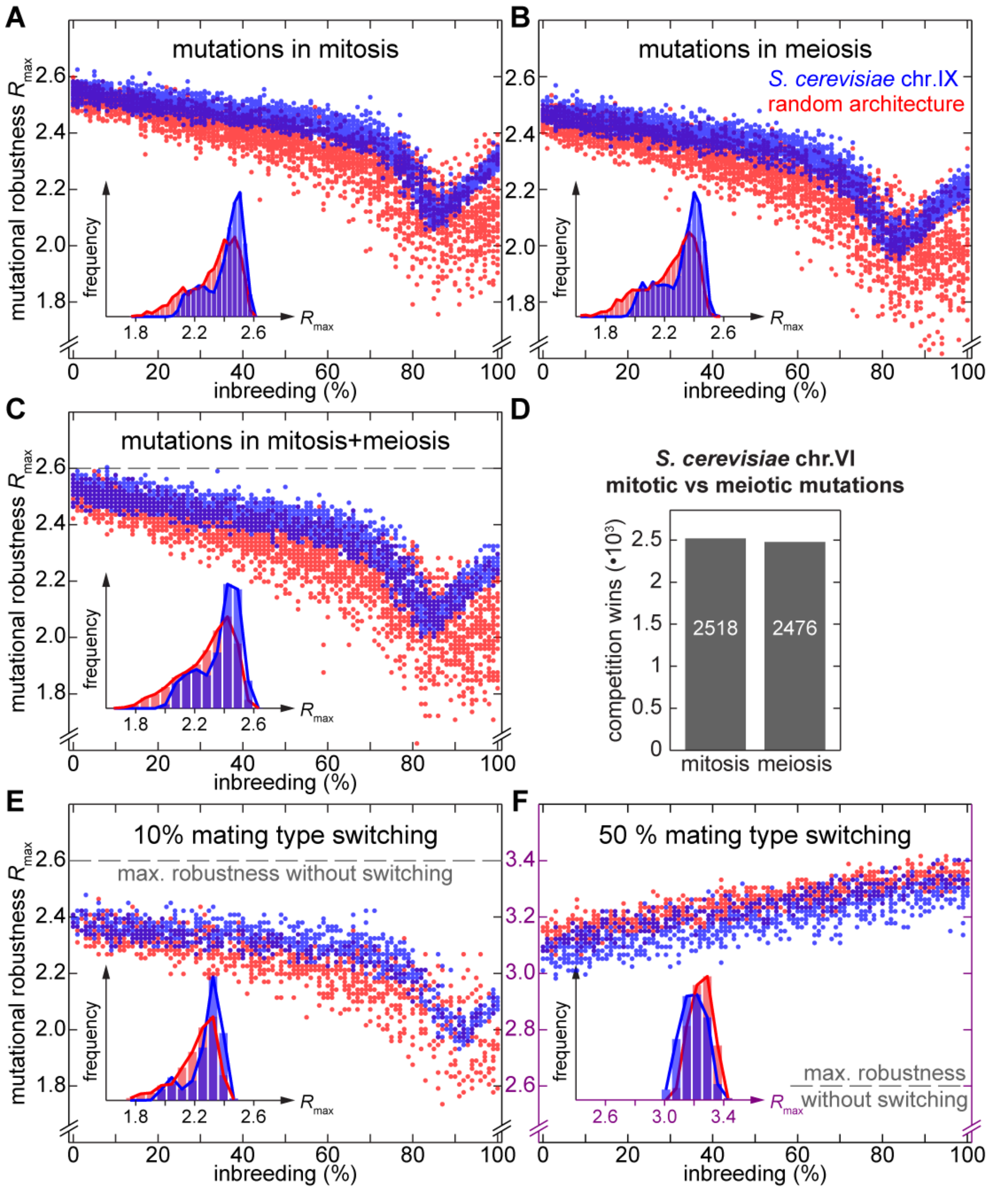

Supplementary Figure 2: Mutational robustness R_{max} of *S. cerevisiae* chromosome IX and random chromosomes

Scatter plots and histograms of mutational robustness (R_{max}) benchmarks performed for populations with *S. cerevisiae* chromosome IX and for populations with random architectures. In the first series of experiments, mutations were applied in mitosis (**A**), in meiosis (**B**) or in mitosis and meiosis (**C**). Notably, almost the same mutational robustness results for these different scenarios. As an additional test, two populations of *S. cerevisiae*

chromosome VI carrying individuals were subjected to a competitive advantage simulation. Mutations were applied exclusively in mitosis in population #1 and exclusively in meiosis in population #2. The histogram in (**D**) shows the competition wins, indicating comparable performance of both populations.

In the second series of experiments, populations with *S. cerevisiae* chromosome IX and populations with random architectures were analyzed for their mutational robustness (R_{max}), considering a contribution of mating type switching of either 10% (**E**) or 50% (**F**) during breeding following meiosis. As a reference, the maximal robustness in the absence of mating type switching is indicated by a grey dashed line. At 10% mating type switching the maximal robustness is decreased by 8%, while it is increased by 42% at 50% mating type switching.

Statistics: n = 3,000 (A-C) or 1,000 (E/F) experiments per chromosome configuration, n = 4,994 experiments (D).

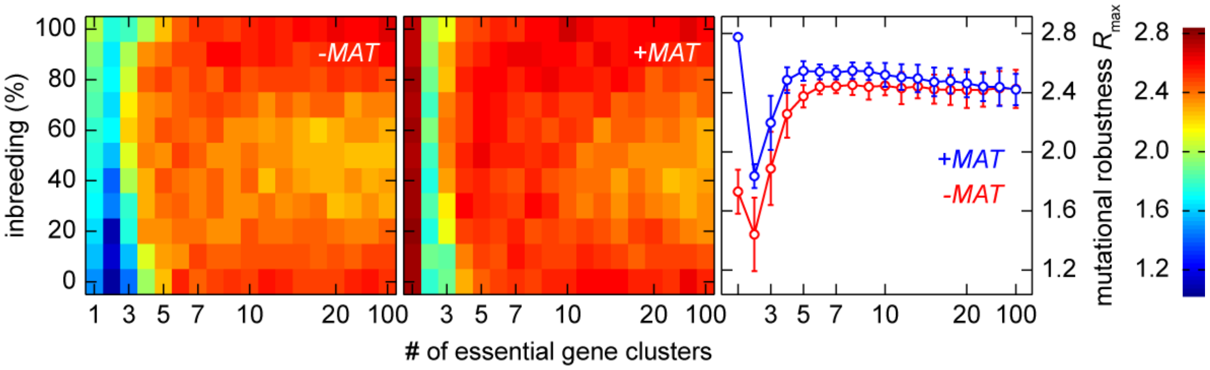

Supplementary Figure 3: The effect of essential gene clustering on mutational robustness at different inbreeding rates

Mutational robustness R_{max} of chromosomes with different levels of essential gene clustering (1-100 clusters, 100 EGs in total) for different mating type configurations (+/-*MAT*) and for different inbreeding fractions (0-100%). In a chromosome architecture with *n* essential gene clusters, each cluster contains *x*/*n* essential genes, where *x* is the total number of essential genes in the chromosome. Thus, the architecture with one essential gene cluster represents a maximally clustered genome, while the architecture with 100 essential gene clusters represents a maximally unclustered genome. R_{max} is color-encoded (left) and provided in dependency of the genome architecture and as a function of the inbreeding fraction. The plot (right) shows the average mutational robustness $\overline{R_{\mathrm{max}}}$ (average of $\mathrm{R}_{\mathrm{max}}$ for the entire inbreeding domain). Error bars indicate SD.

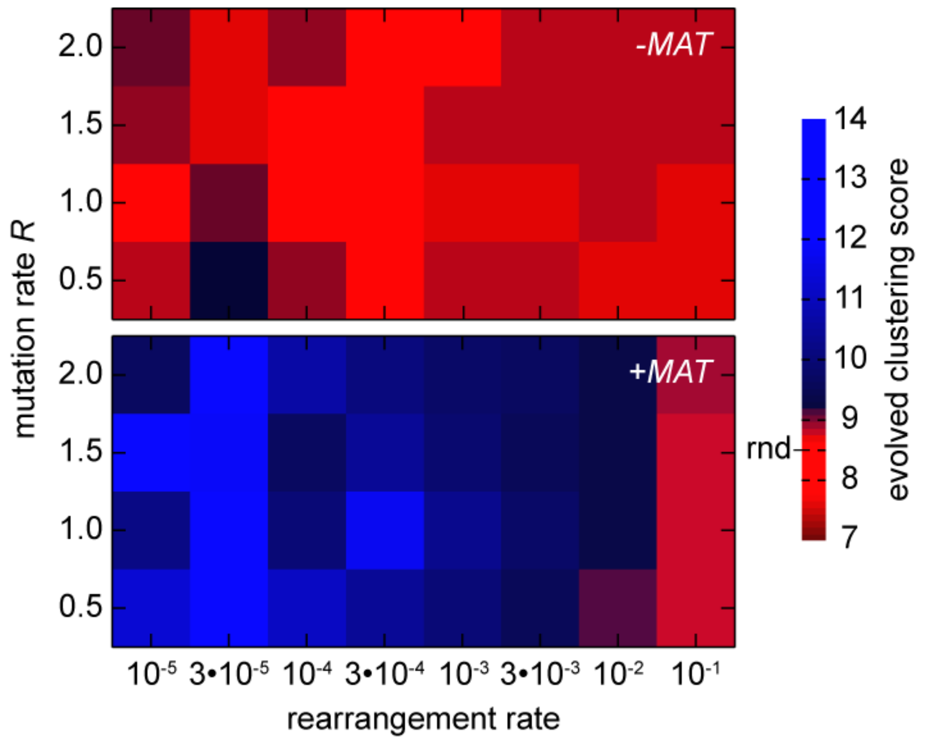

Supplementary Figure 4: Evolution of pericentromeric EG clustering requires a mating type

Levels of essential gene clustering obtained in the evolution of the small model architecture shown in Figure 7C in Main Text. The evolution experiment was performed for different rearrangement rates, mutations rates and mating type configurations (+/-*MAT*). The chromosomes contain five essential genes, five non-essential genes, four recombination hotspots and five recombination coldspots. The initial genome architecture is maximally unclustered (resulting in a clustering score of $\nu = 5$). The evolution of the architectures was simulated for 100,000 generations. Without a mating type, the clustering score does not increase beyond the level found in random populations (between 8 and 9). In the presence of a mating type, the architectures evolve towards a clustering score between 10 and 13 for rearrangement rates below 10^{-3} per genetic element and generation. The highest possible level of essential gene clustering is reflected by a clustering score of 25 (all five essential genes in a single cluster without recombination hotspots). “rnd” indicates the average clustering score of randomly generated genomes ($\nu = 8.5$).

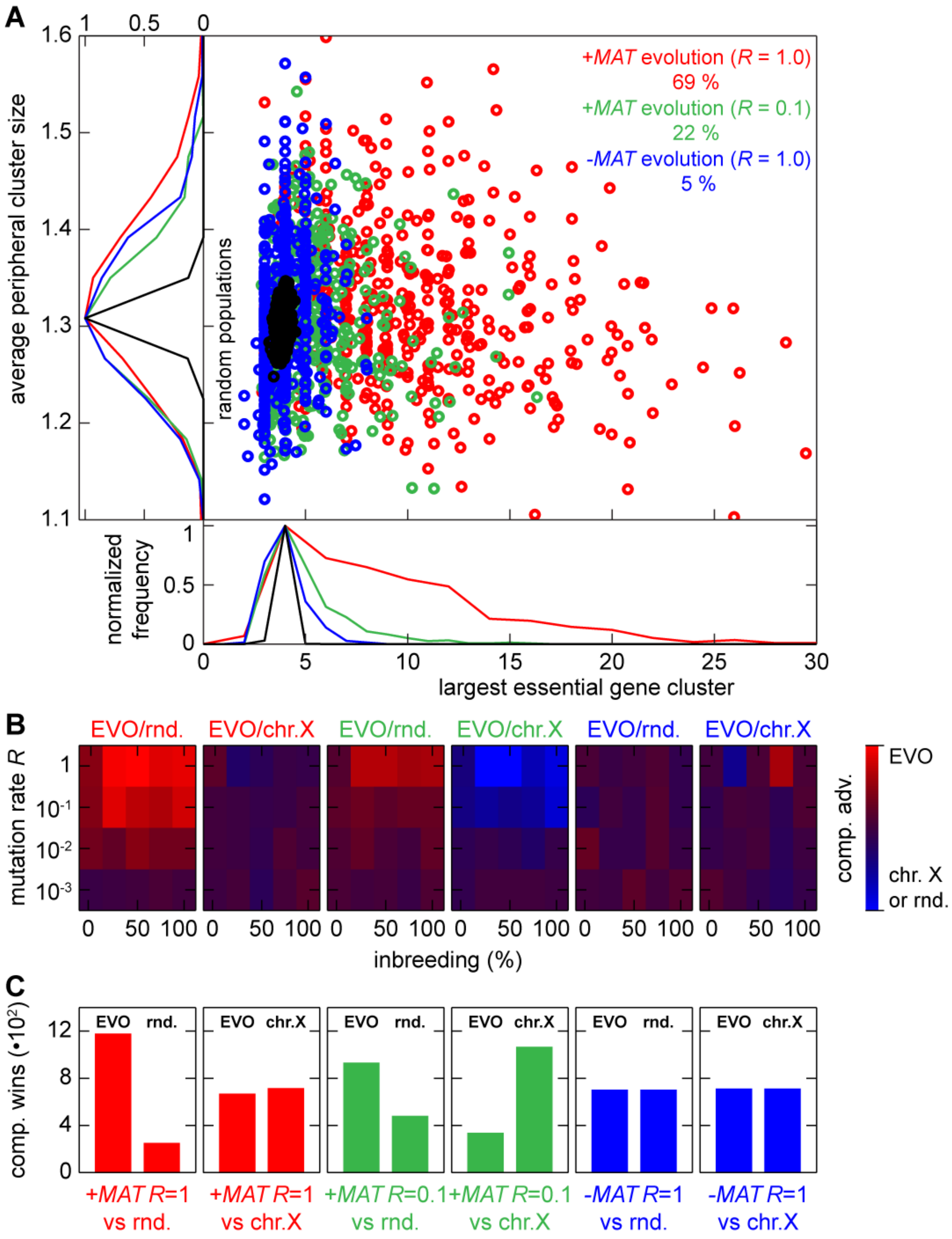

Supplementary Figure 5: Evolution of essential gene clustering in *S. cerevisiae* X-like chromosomes

(**A**) Evolution of chromosome architectures with a yeast chromosome X-like size and genetic content. Starting with completely unclustered chromosomes, populations with and without

MAT were allowed to evolve at different mutation rates R as indicated ($n_{red} = 495$, $n_{green} = 500$, $n_{blue} = 500$ experiments). Similar to the analysis in Figure 8A in Main Text, clustering was scored by measuring both the size of the largest essential gene cluster and the average size of the remaining clusters. For genomes containing a *MAT*, the largest cluster was always observed to be linked to the *MAT*. The scatter plot shows the scores obtained after an evolution period of 200,000 generations. Score distributions for the different populations are spanned along the axes (including reference distributions for randomly generated populations of the same size as in the +*MAT* scenario at $R = 1$). The unclustered starting architecture provides a clustering score of $v = 131$. Random architectures with a chromosome X genetic content provide a clustering score of $v = 221 \pm 23$ (SD), whereas the chromosome X architecture itself scores $v = 408$. The percentages indicate the fraction of experiments yielding genomes with a clustering value v at least 2σ above the average score of randomly generated genomes (i.e. $v \geq 267$).

(**B**) Survival competition of the evolved genomes shown in (**A**) versus chromosome X and random genome architectures. The matrices show the average statistical results for $n = 300$ evolved genomes picked randomly from the red, green and blue groups.

(**C**) The bars indicate the total number of wins of the evolved architectures and of chromosome X or the random architectures respectively in the competition experiments shown in (**B**).

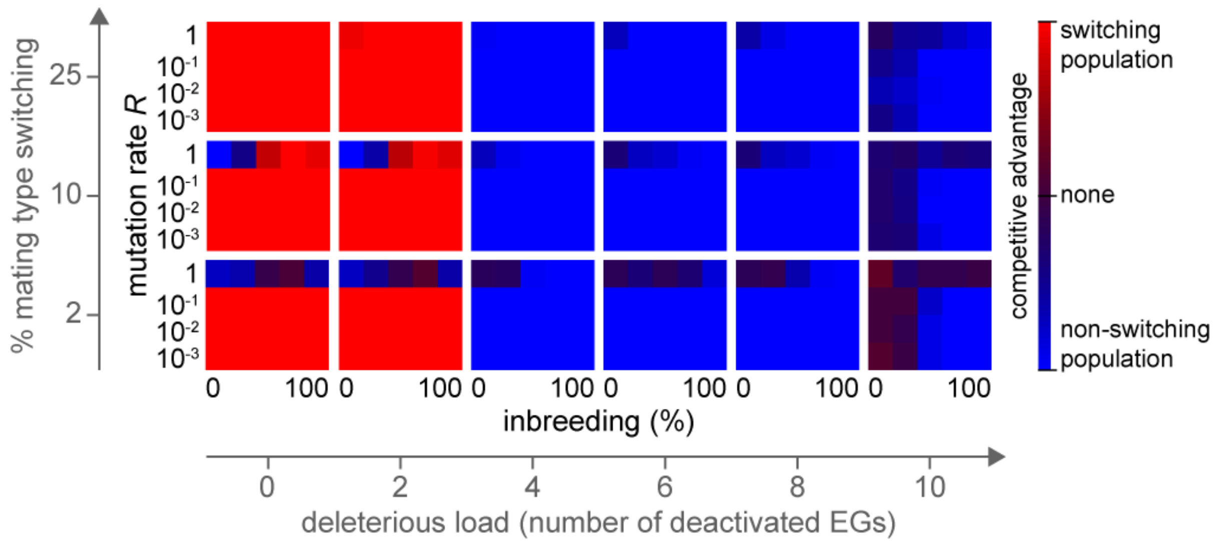

Supplementary Figure 6: Competition analysis of mating type switching populations vs. non-switching populations at different levels of deleterious pre-load

Survival competition experiments of populations with *S. cerevisiae* chromosome VI subjected to different levels of mating type switching (2%, 10% and 25%) versus populations that do not perform mating type switching (*n* = 2,000 experiments per matrix). Before the start of the competition, 0, 2, 4, 6, 8 or 10 essential genes were deactivated (distributed on both homologues). When starting with two or less essential gene mutations in the entire chromosome, the switching populations clearly outperformed the non-switching populations for mutations rates between 10^{-3} and 10^{-1}. The situation is reversed in the presence of more than two mutations in the chromosome: non-switching populations win against switching populations. At high mutation rates (R = 1), non-switching populations have a strong quantitative advantage, even in the absence of a mutational pre-load. Only when competing with populations subjected to high fractions of mating type switching (≥ 25%) with a mutational pre-load of less than four essential gene mutations, the switching populations outperform non-switching populations at R = 1.

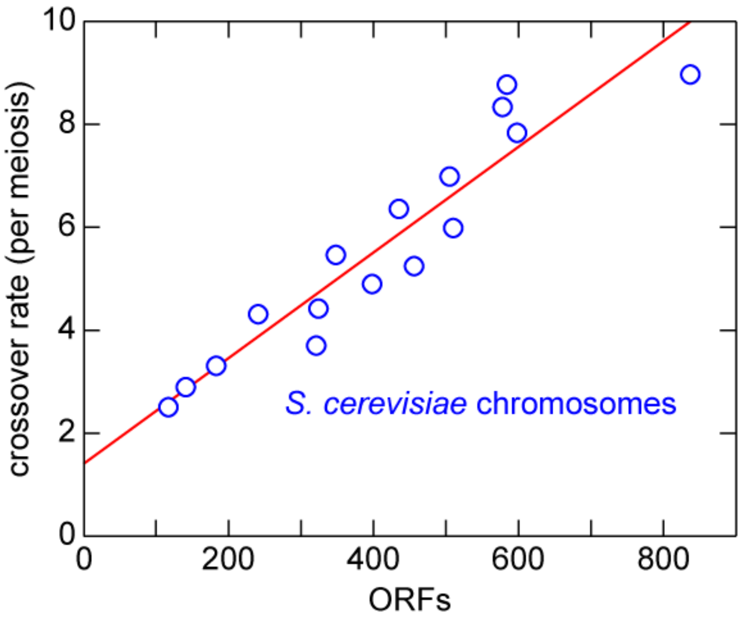

Supplementary Figure 7: Number of crossovers and ORFs in *S. cerevisiae* chromosomes

Crossover frequencies and ORF content of the 16 chromosomes of *S. cerevisiae*. Data was compiled from www.yeastgenome.org.

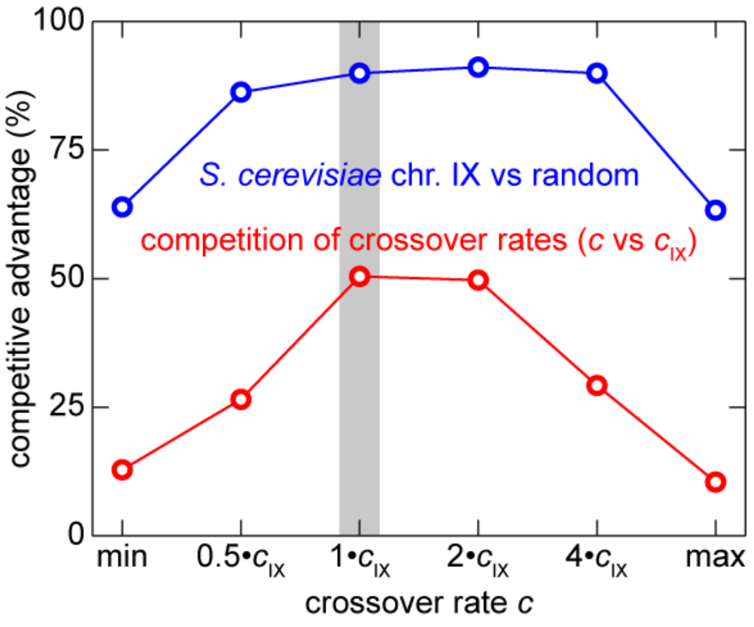

Supplementary Figure 8: Competition analysis of crossing over rates in yeast chromosomes

Average competitive advantage in the experiments shown in Figure 10B/C in Main Text. The direct competition of the *S. cerevisiae* chromosome IX crossover rate (c_{IX}) versus altered crossover rates (shown in red) reveals a global performance maximum at the *S. cerevisiae* chromosome IX rate. In this regime, *S. cerevisiae* chromosome IX also performs particularly well against random architectures (shown in blue). The SEM errors bars are smaller than the data circles.

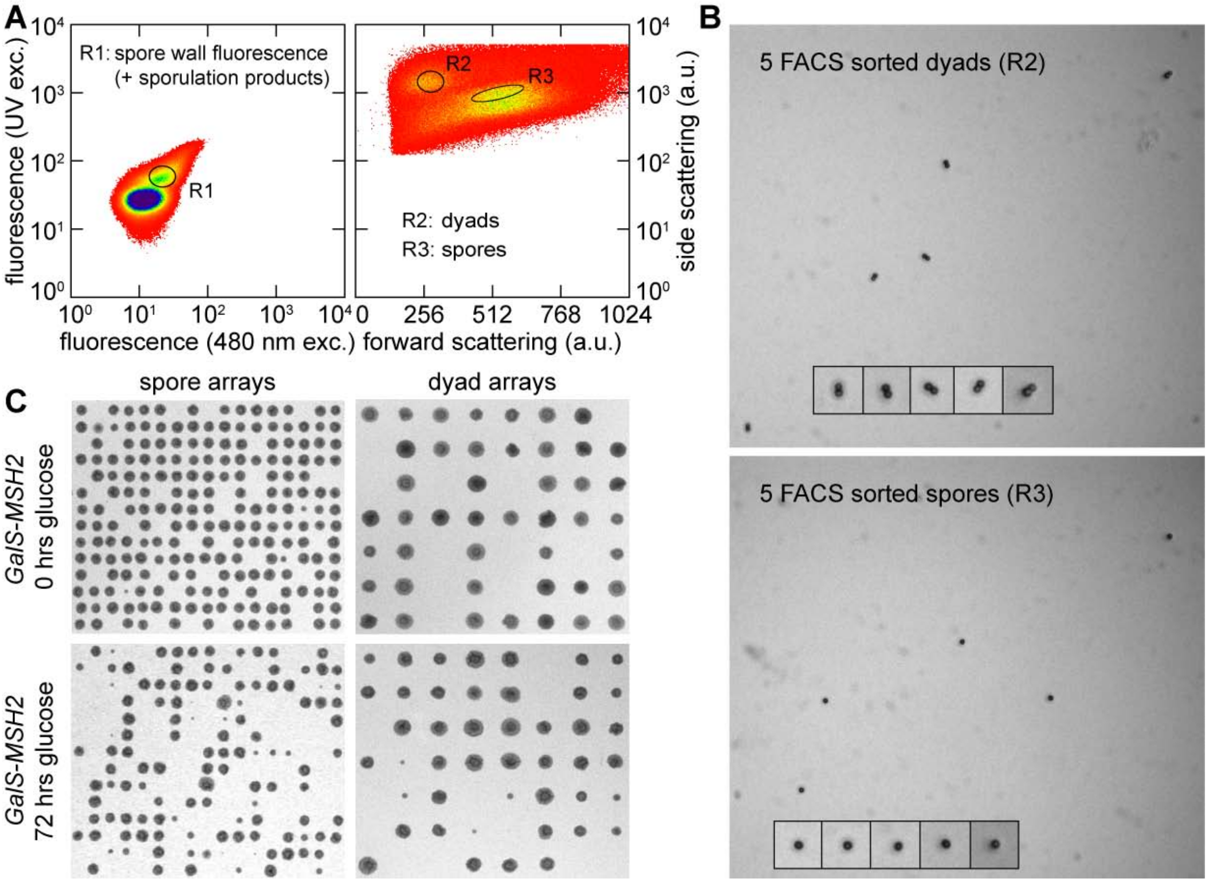

Supplementary Figure 9: FACS sorting of spores and dyads

(**A**) Spores were gated based on green autofluorescence of the cells and ultraviolet autofluorescence of the spore wall (R1, excitation/emission: 326/404 nm). Spores (R3) and dyads (R2) could be distinguished using shape parameters determined from side and forward scatter.

(**B**) To assess the sorting specificity, 500 spores and 500 dyads were sorted onto an agar plate in 50 groups of 10 and counted using a microscope equipped with a 20x magnification air lens. The dyad plate was contaminated with tetrads (0.2%), triads (3.2%) and single spores (0.7%), whereas the spore plate was effectively contamination-free.

(**C**) Examples of sorted spores and dyads grown on YP-galactose/raffinose (2% each) plates. Colony formation was scored using ImageJ (NIH, Bethesda) to count colonies in the plate images. A spore was considered to be viable when it formed a colony that could be clearly identified by the image analysis procedure.

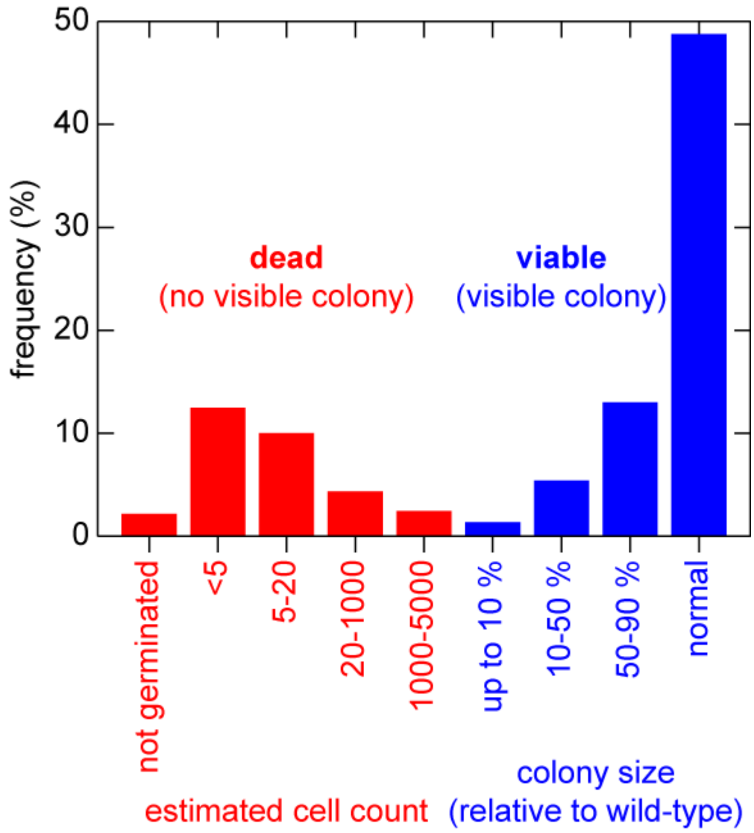

Supplementary Figure 10: Germination efficiency and colony size distribution of FACS-sorted single spores

Histogram of the distribution of colony sizes of sorted single spores upon mutation accumulation for 33-36 generations in the conditional Msh2 mutator strain. Sorted spores that did not give rise to visible colonies were investigated with microscopy and categorized according to their approximate cell counts. Visible colonies were categorized according to their diameter relative to the average wild type colony size (wild type = colonies with no obvious growth defect).

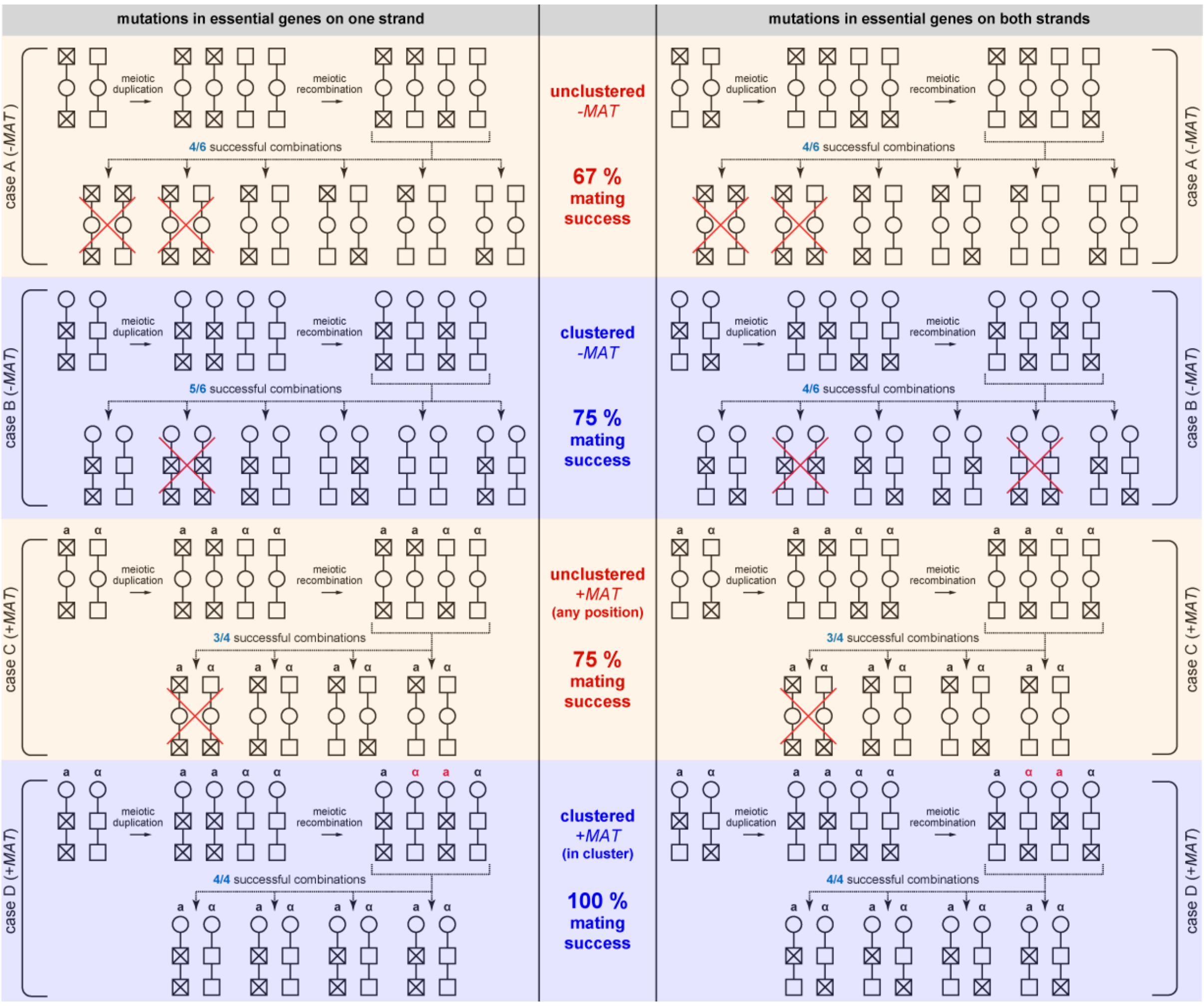

Supplementary Figure 11: Mating success rates in the presence of mutational load

A simplified model that illustrates the increase in mating success due to essential gene clustering and *MAT*-linkage in the case of pure inbreeding and a two-gene configuration with a single recombination hotspot. The model considers a static scenario of maximum load (50% inactivated EGs). The genome undergoes a meiotic duplication and a meitotic recombination of two sister chromatids. The six possible pairings of the resulting four haploids are shown as schematic illustrations. Large red crosses indicate lethal combinations. All possible architectures (unclustered = hotspot between genes, clustered = non-separated genes), mutation distributions (on one chromosome or on homologue strands) and mating type configurations (with/without *MAT*) were considered. Clustered architectures provide higher survival rates than unclustered architectures. The presence of a *MAT* further increases the chance of obtaining a viable combination. No lethal combination is possible for a clustered *MAT*-linked architecture (100% mating success).

This scenario was chosen to highlight the correlation between clustering and the viability after mating. It does not reflect the complex processes of the dynamic life cycle, in which the load is also affected by the linkage relationship of the essential genes, as determined by their distribution and the distribution of meiotic recombination hotspots. In the dynamic scenario,

populations with different chromosome architectures will accumulate different levels of load, which are specific to their architecture and the environmental conditions. This circumstance will affect the lethality caused by homozygotisation of lethal mutations and the associated purging of lethal load.

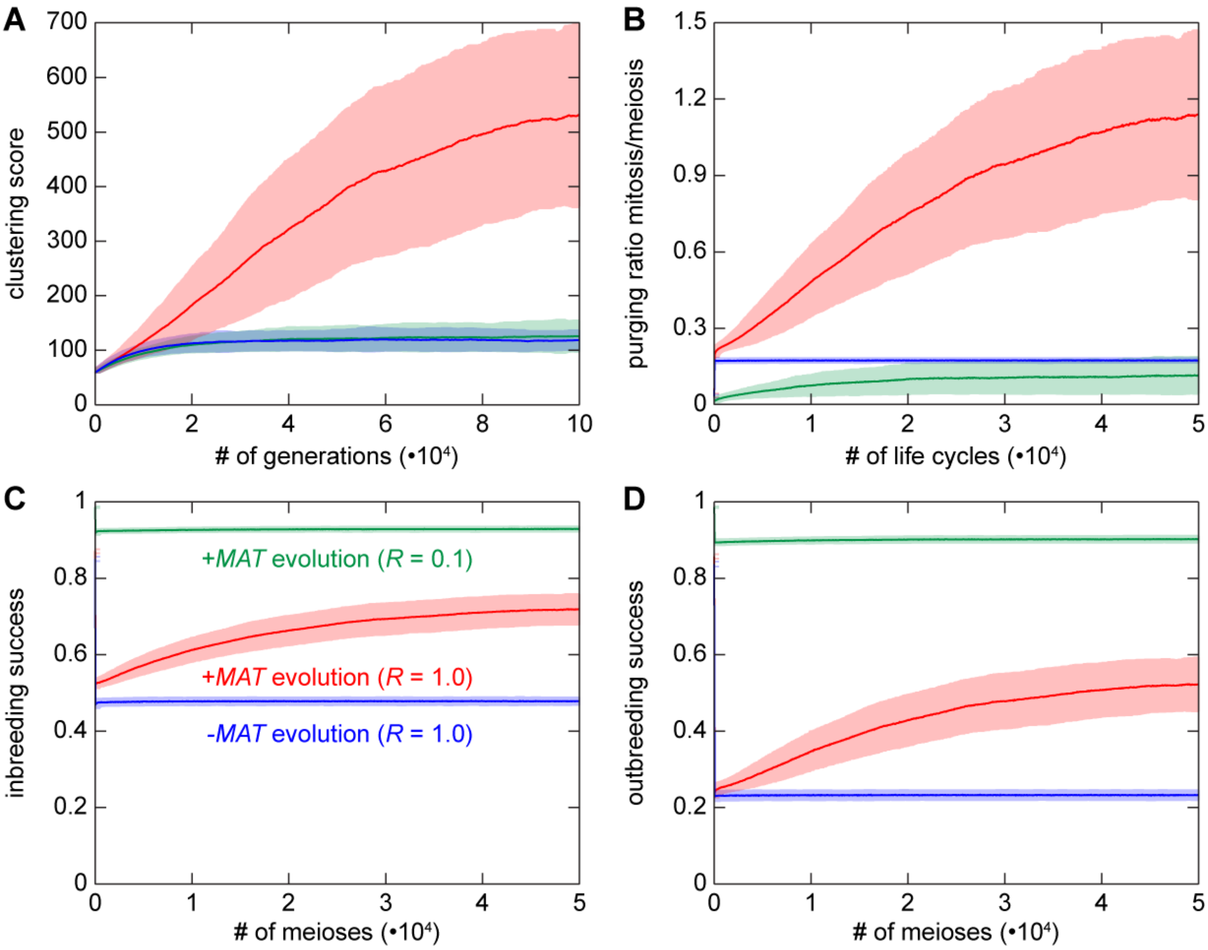

Supplementary Figure 12: Purging and survival rates in the evolution of clustering experiment

Average clustering scores (**A**), purging ratios (mitosis over meiosis, **B**), inbreeding success rates (**C**) and outbreeding success rates (**D**) as a function of simulated generations for the three types of evolution experiments discussed in Figure 8A in Main Text. The light-colored areas indicate standard deviations over the entire set of experiments (n = 816 high-R +*MAT* populations [red], n = 816 low-R +*MAT* populations [green], n = 825 high-R -*MAT* populations [blue]). In all experiments, inbreeding and outbreeding success rates are positively correlated to the clustering score. This effect is particularly strong in high-R +*MAT* populations.

Video S1: Maintenance of EG clustering at low and at high mutation rates

Maintenance of essential gene clustering in evolving inbreeding populations for two of the data points in Figure 7A in Main Text. The right panel corresponds to data point #1, while the one to the left corresponds to data point #2. The graphs at the top show the genomic element densities for the entire population. Chromosomes are vertically aligned. The color-coding reports the differences in density of EGs and recombination hotspots (sliding window analyses of individual genomes, window size is 20 elements). Histograms of the sliding window analyses are shown at the bottom. Initially, all EGs are located on one side of the chromosomes, while recombination hotspots form a cluster in the middle of the non-essential genes (at the other end of the chromosome). At high mutation rates, the EG clustering is maintained (left, movie shows a period of 100,000 generations). At low mutation rates, EG clustering is not maintained and the EGs become distributed in random patterns (right). Both simulations used identical values for all parameters except for *R*.

Video S2: Evolution of EG clustering

Visualization of the evolution of clustered genome architectures as a function of time in a population representative for the high-*R* +*MAT* evolution experiment shown in Figure 8A in Main Text. The top left panel shows a density difference sliding window plot of all genomes (red = only essential genes, blue = only hotspots, green = equal densities of essential genes and hotspots). The bottom two panels show essential gene and hotspot densities in the entire population. The average clustering score in the population (white, SD in red) is indicated in the top right panel (dashed white line = level of clustering in random architectures [107 ± 17]). The simulation starts with a maximally unclustered genome architecture. The inbreeding percentage is 50%. Movie playback speed: 6,000 generations per second (400 generations per frame).

Table 1: Essential gene clustering in *S. cerevisiae*

***S. cerevisiae* chromosome statistics**					***S. cerevisiae* chromosome analysis**				**Random reference architecture analysis**						**Comparison**
#	EGs	non-EGs	hot-spots	cold-spots	clustering score	largest EG cluster	peripheral clustering		clustering score		largest cluster		peripheral clustering		clustering level above random mean (in SD units)
							mean	SD	mean	SD	mean	SD	mean	SD	
**1**	15	76	38	52	**96,20**	5	1,11	0,33	**54,59**	**10,13**	2,31	0,66	1,12	0,27	**4,11**
**2**	74	337	172	238	**327,30**	5	1,38	0,64	**258,71**	**24,09**	3,47	0,83	1,21	0,46	**2,85**
**3**	17	139	73	82	**91,41**	2	1,07	0,27	**90,03**	**13,06**	1,97	0,56	1,06	0,16	0,11
**4**	174	609	277	505	**559,20**	5	1,47	0,82	**499,91**	**34,52**	4,85	1,05	1,37	0,67	**1,72**
**5**	45	223	113	154	**169,73**	3	1,20	0,47	**163,81**	**19,11**	2,95	0,78	1,18	0,40	0,31
**6**	27	94	57	63	**143,93**	6	1,24	0,56	**84,99**	**12,43**	2,76	0,75	1,17	0,39	**4,74**
**7**	113	432	223	321	**446,02**	5	1,42	0,79	**355,62**	**28,41**	3,91	0,87	1,27	0,54	**3,18**
**8**	43	215	96	161	**232,35**	7	1,29	0,53	**149,07**	**18,94**	3,23	0,82	1,21	0,45	**4,40**
**9**	37	164	99	101	**132,43**	3	1,10	0,30	**136,42**	**15,16**	2,69	0,68	1,14	0,35	-0,26
**10**	72	285	130	226	**407,56**	10	1,59	0,82	**220,80**	**22,87**	3,90	0,93	1,30	0,57	**8,17**
**11**	69	263	129	202	**239,28**	4	1,41	0,65	**212,42**	**22,35**	3,72	0,89	1,28	0,54	**1,20**
**12**	100	418	209	308	**369,60**	4	1,35	0,66	**326,55**	**28,09**	3,77	0,86	1,25	0,52	**1,53**
**13**	76	400	179	296	**274,74**	3	1,26	0,52	**273,26**	**25,38**	3,47	0,81	1,23	0,48	0,06
**14**	74	333	145	261	**244,65**	4	1,30	0,57	**239,92**	**24,49**	3,80	0,89	1,28	0,55	0,19
**15**	100	447	198	348	**358,20**	4	1,37	0,68	**325,40**	**29,25**	3,99	0,96	1,28	0,56	**1,12**
**16**	96	377	188	284	**413,44**	7	1,39	0,77	**303,63**	**27,58**	3,90	0,93	1,27	0,54	**3,98**

Conservative estimate of essential gene clustering using whole-genome data from Mancera *et al.* (2008) [1]. Note that these data yield a clustering score for yeast chromosome IX that differs from the data provided by Pal and Hurst (2003) [12]. The data set used for the initial chromosome IX architecture is based on the hotspot distribution from Gerton *et al.* (2000) [5], whereas the data from Mancera *et al.* (2008) [1] used for the analyses underlying Table 1 report on the distribution of actual crossover events. Detailed information about the architectures used in our simulation experiments are provided in Text S1, Section E.

Table 2: The parameters and simulation modules of *S. digitalis*

The table lists all parameters, modules and simulation functionality in *S. digitalis*. The valid parameter space is indicated for each parameter. Some parameters are not accessible via the function interface and are therefore listed as "internal" parameters (see second part of the table).

**Command line parameters of the simulation *S. digitalis* (version 1.79, 10/2005-02/2009)**				
Parameter types – ***red*: simulation core parameters** | ***green*: optional module parameters** | ***blue*: simulation layout parameters**				
**#**	**type**	**parameter name**	**parameter description**	**Restrictions**
**1**	● (red)	nGenes	number of genes per chromosome	$x \in \mathbb{N}$
**2**	● (red)	nEssentialGenes	number of essential genes per chromosome	$x \in \mathbb{N}_0$
**3**	● (red)	nRPIE	number of recombination hotspots	$x \in \mathbb{N}; x \leq (n_{genes} - 1)$
**4**	● (red)	mutationRates	mutation rate(s)<br>note: considered as initial rate(s), if feedback is active (see #29)	$x \in \mathbb{R}_0^+$
**5**	● (green)	mutationRateSpacing	precision of robustness determination for survival screen experiments<br>note: alternatively a list of mutation rates can be provided via parameter #4	$x \in \mathbb{R}_0^+$
**6**	● (red)	rearrangementRates	rearrangement rate(s)	$x \in \mathbb{R}_0^+$
**7**	● (blue)	statisticalAveraging	number of iterations of the simulated experiments	$x \in \mathbb{N}$
**8**	● (green)	seedingSetup	structural layout of the seeding genome<br>note: provides access to templates, see code for details	$x \in \mathbb{Z}; 0 \leq x \leq 13$
**9**	● (red)	clusterFrequency	cluster frequency of the seeding genome<br>note: only required if parameter #8 is set to 12 or 13	$x \in \mathbb{N}; x \leq 100$
**10**	● (green)	clusterFrequencyArray	cluster frequency array of the seeding genome<br>note: only required for seeding knockout survival screen (see #14)	$x \in \mathbb{N}; x \leq n_{genes}$

**11**	●	seedingKnockout	fraction of mutated essential genes at the start of the simulation	$\left(x \cdot n_{genes}\right) \in \mathbb{Z}; 0 \leq x \leq 0.5$
**12**	●	seedingKnockoutArray	fraction array of mutated essential genes at the start of the simulation<br>note: only required for seeding knockout survival screen (see #14)	$\left(x \cdot n_{genes}\right) \in \mathbb{Z}; 0 \leq x \leq 0.5$
**13**	●	fittingScheme	indicator for fitting type of clustering values<br>settings: 0 for linear fitting<br>1 for exponential fitting	$x \in \{0;1\}$
**14**	●	performSurvivalScreen	indicator for screening type<br>settings: 0 for no screening<br>1 for mutation/rearrangement rate survival screen<br>2 for seeding knockout ratio survival screen	$x \in \{0;1;2\}$
**15**	●	screeningArray	indicator for mutation rate or rearrangement rate screen<br>note: only required, if parameter #14 is set to 1<br>settings: 1 for rearrangement rate screen<br>2 for mutation rate screen	$x \in \{1;2\}$
**16**	●	positiveSurvivalRuns	number of successful experiments before switching the screening column<br>note: only required for screening experiments	$x \in \mathbb{N}$
**17**	●	totalSimulationSteps	simulated time period (measured in half life cycles)	$\left(x/2\right) \in \mathbb{N}$
**18**	●	inbreedingFraction	inbreeding fraction in the mating process<br>note: two parameters can be provided in survival competition experiments	$x \in \mathbb{R}; 0 \leq x \leq 1$
**19**	●	requiredHomologousFraction	level of homology required for genome survival<br>note: only required if #44 is set to 0 and outbreeding fraction is non-zero	$\left(x \cdot n_{genes}\right) \in \mathbb{Z}; 0 \leq x \leq 1$
**20**	●	rearrangementMode	indicator for type of rearrangement mode<br>settings: 0 for switching<br>1 for flipping	$x \in \{0;1\}$
**21**	●	recombinationProbability	probability of meiotic recombination (per tetrad)<br>note: two parameters can be provided in survival competition experiments	$x \in \mathbb{R}; 0 \leq x \leq 1$
**22**	●	recombinationUnitFactor	scaling factor for adjustment of crossover rate<br>note: two parameters can be provided in survival competition experiments	$x \in \mathbb{R}^{+}$

**23**		recombinationMode	indicator for the type of recombination position determination<br>settings: 0 for an Erlang-based computation (crossover interference)<br>1 for random positions	$x \in \{0;1\}$
**24**		erlangApplicationFlag	indicator for the distance mode that is used for the Erlang distribution<br>settings: 0 for physical distance<br>1 for genetic distance	$x \in \{0;1\}$
**25**		randomApplicationFlag	indicator for the type of recombination event number determination<br>note: only required, if parameter #23 is set to 1<br>settings: 0 for an Erlang-based event number<br>1 for a constant number of events	$x \in \{0;1\}$
**26**		matingLocusFlag	indicator for the presence of a mating type locus<br>settings: 0 for deactivation<br>1 for activation	$x \in \{0;1\}$
**27**		matingLocusPosition	position of the mating type locus on the genome<br>notes: only required if parameter #26 is set to 1,<br>two parameters can be provided in survival competition experiments	$x \in \mathbb{N}; x \leq (2n_{genes} - 1)$
**28**		externalLocusFlag	indicator for the definition of an externally-linked mating type locus<br>settings: 0 for internal mating type locus<br>1 for mating type locus on external chromosome	$x \in \{0;1\}$
**29**		matingSwitching	mating type switching fraction in the mating process<br>note: two parameters can be provided in survival competition experiments	$x \in \mathbb{R}; 0 \leq x \leq 1$
**30**		adaptiveMutationRate	indicator for the activation of a mutation rate feedback<br>settings: 0 for deactivation<br>1 for activation	$x \in \{0;1\}$
**31**		relativeFeedbackFlag	indicator for the type of mutation rate feedback<br>note: only required if #29 is set to 1<br>settings: 0 for an absolute feedback computation<br>1 for a relative feedback computation	$x \in \{0;1\}$
**32**		feedbackPopulationSize	population size feedback coefficient<br>note: only required if #29 is set to 1	$x \in \mathbb{R}; 0 \leq x \leq 1$
**33**		feedbackPopulationSizeDerivative	population size variation feedback coefficient<br>note: only required if #29 is set to 1	$x \in \mathbb{R}; 0 \leq x \leq 1$

**34**	●	derivativeDistance	time interval that is considered in the computation of population size variations (measured in life cycles)<br>note: only required if #29 is set to 1	$x \in \mathbb{N}$
**35**	●	populationCapSize	indicator for a population size cap<br>settings: 0 for deactivation<br>1 for activation	$x \in \{0;1\}$
**36**	●	initialOrMaxGenomes	maximum number of genomes in the population matrix<br>note: if #34 is set to 0, this is only the initial number of genomes	$x \in \mathbb{N}$
**37**	●	protocolSplittingSize	time period (in generations) that is covered per protocol file	$x \in \mathbb{N}, 2 \leq x \leq 1000$
**38**	●	databaseResolution	temporal spacing (in generations) between two population matrix snapshots	$x \in \mathbb{N}; x \geq 2$
**39**	●	numericalCorrectionFactor	normalization factor for the Erlang distribution that allows setting up a reference crossover rate<br>note: the crossover rate can be modulated by parameter #22	$x \in \mathbb{R}^+$
**40**	●	skipMeiosisFlag	indicator for the simulation of purely vegetative reproduction<br>settings: 0 for standard mitosis-meiosis cycles<br>1 for mitosis-only cycles	$x \in \{0;1\}$
**41**	●	randomizeCycles	indicator for a randomization of the starting point in the life cycle at the beginning of the simulation<br>settings: 0 for no randomization (starting point: mitosis)<br>1 for randomization (starting point: either mitosis or meiosis)	$x \in \{0;1\}$
**42**	●	mutationCycleFrequency	temporal spacing (in mitoses) between the determination of random mutation events<br>note: the standard setting 1 applies random mutations at each mitosis	$x \in \mathbb{N}$
**43**	●	mutationMode	indicator for life cycle steps, in which mutations can occur<br>settings: 0 for mitosis only<br>1 for meiosis only<br>2 for mitosis and meiosis	$x \in \{0;1;2\}$
**44**	●	subPopulationFlag	indicator for the dynamic definition of architecture-linked sub-populations<br>note: sub-populations are treated as isolated domains in the mating-module	$x \in \{0;1\}$

**45**	●	displayInfoFlag	indicator for displaying of simulation statistics at runtime<br>settings: 0 for deactivation of runtime information<br>1 for activation of runtime information	$x \in \{0;1\}$
**46**	●	loadRandomGeneratorSeed	indicator for the seeding type of the random number generator<br>settings: 0 for seeding with the current system time<br>1 for reading of seeding number in “randomGenerator.mat”	$x \in \{0;1\}$
**47**	●	silentIOFlag	indicator for minimal disk space mode<br>settings: 0 for recording of standard protocols at runtime<br>1 for recording of final simulation results only	$x \in \{0;1\}$
**48**	●	analyzeClusteringFlag	indicator for clustering score determination at simulation run-time (1=yes, 0=no)	$x \in \{0;1\}$
**49**	●	frontendFlag	indicator for *S. digitalis* being invoked by a frontend program (1=yes, 0=no)<br>note: allows returning survival competition results as a data array	$x \in \{0;1\}$
**50**	●	clusterModeFlag	indicator for deployment on computer cluster or single workstation<br>settings: 0 for computer cluster<br>1 for single workstation	$x \in \{0;1\}$
**51**	●	centralRepository	runtime and output directory	*String*
**52**	●	centralSeeding	input directory for population seeding database	*String*
**53**	●	touchDirectory	directory name to be subjected to the “touch” command in regular intervals<br>note: prevents data loss on cluster file systems with automated clean-up settings	*String*

**Internal parameters of the simulation *S. digitalis* (version 1.79, 10/2005-02/2009)**					
**#**	**type**	**parameter name**	**parameter description**	**restrictions**	**preset**
**54**	●	homozygosityAdvantage	statistical selection factor for a homozygous hotspot as compared to a single hotspot during meiotic recombination	$x \in \mathbb{R}^+$	2
**55**	●	printoutSeedingGenome	indicator for the visualization of the initial population matrix<br>settings: 0 for deactivation<br>1 for activation	$x \in \{0;1\}$	0

**56**	●	saveASCIIFiles	indicator for the storing of arrays in a text editor compatible format<br>settings: 0 for deactivation<br>1 for activation	$x \in \{0;1\}$	0
**57**	●	printoutMitoticGenome	indicator for the visualization of the population matrix in mitosis<br>settings: 0 for deactivation<br>1 for activation	$x \in \{0;1\}$	0
**58**	●	printoutMeioticGenome	indicator for the visualization of the population matrix in meiosis<br>settings: 0 for deactivation<br>1 for activation	$x \in \{0;1\}$	0
**59**	●	createGenomicDatabase	indicator for the storing of population matrix snapshots<br>settings: 0 for deactivation<br>1 for activation	$x \in \{0;1\}$	1
**60**	●	minimalDiskSpaceMode	indicator for the activation of a non-redundant protocol mode<br>settings: 0 for deactivation<br>1 for activation	$x \in \{0;1\}$	1
**61**	●	analyzeClustering	indicator for a computation of the population matrix clustering value<br>settings: 0 for deactivation<br>1 for activation	$x \in \{0;1\}$	1
**62**	●	clusterAnalysisPower	power parameter in the definition of the clustering value	$x \in \mathbb{R}^+$	2
**63**	●	visualizeProtocols	indicator for the generation of JPEG plots of the protocol arrays<br>settings: 0 for deactivation<br>1 for activation	$x \in \{0;1\}$	1
**64**	●	fullFitnessIdentifier	offset for the identification of functional essential genes in the population matrix	$\left(x/1000\right) \in \mathbb{N}; x \le 64000$	1000
**65**	●	noFitnessIdentifier	offset for the identification of mutated essential genes in the population matrix	$\left(x/1000\right) \in \mathbb{N}; x \le 64000$	2000
**66**	●	matingLocusIdentifier	offset for the identification of the mating type locus in the rearrangement protocols	$\left(x/1000\right) \in \mathbb{N}; x \le 64000$	3000
**67**	●	derivativeFeedbackCap	cut-off value for parameter #32	$x \in \mathbb{R}^+$	0.1

**68**	●	slidingWindowSize	size of the sliding window filter that is used for the histogram analysis	$x \in \mathbb{N}; x \leq n_{genes}$	10
**69**	●	slidingWindowPlotSize	size of the sliding window filter that is used for the visualization of the population matrix	$x \in \mathbb{N}; x \leq n_{genes}$	20

E) *S. digitalis* Simulation Settings

Experimental settings for **Figure 4A**: Mutational robustness R_{max} of *S. cerevisiae* chromosome IX and random architectures with the same number of essential genes and recombination hotspots	
population size	200
crossover interference	Active
mating type	active and linked to the centromere of a (virtual) chromosome; the centromere of the simulated chromosome is located at position 333, which is equivalent to the centromere-position of chromosome IX.
recombination rate	2.63 crossovers per chromosome and meiosis
inbreeding percentage	0-100% in 1% steps
mutational robustness R_{max} precision (step size)	0.007<br>Note: the scored R_{max} values denote the smallest values *R*, at which a population (specified by either chromosome IX or a randomly generated genome, and a particular inbreeding ration) became extinct within the maximally allowed number of generations.
simulation time	2,000 generations
total statistics	3,000 experiments per subset (chromosome IX and random)
chromosome architecture	*S. cerevisiae* chromosome IX building blocks (35 essential genes, 172 non-essential genes, 58 recombination hotspots, 148 recombination coldspots)<br>Note: random chromosomes contained the same number of elements as the yeast chromosome, but using a random distribution that was calculated independently for each simulation run.

Experimental settings for **Figure 4B**: Survival competition of *S. cerevisiae* chromosome IX vs. random architectures with the same number of essential genes and recombination hotspots	
population size	2,000
crossover interference	Active
mating type	active and linked to the centromere of an (virtual) chromosome; the centromere of the simulated chromosome is located at position 333, which is equivalent to the centromere-position of chromosome IX.
recombination rate	*S. cerevisiae* chromosome IX rate
inbreeding percentage	0-100% in 5% steps
mutation rate *R*	0.0-2.8 in 0.07 steps
simulation time	max. 10,000 generations; the simulation stopped as soon as one species became extinct.
total statistics	10 experiments per grid point
chromosome architecture	*S. cerevisiae* chromosome IX building blocks (35 essential genes, 172 non-essential genes, 58 recombination hotspots, 148 recombination coldspots)<br>Note: random chromosomes contained the same number of elements as the yeast chromosome, but using a random distribution that was calculated independently for each simulation run.

Experimental settings for **Figure 4C**: Survival competition of *S. cerevisiae* chromosome IX vs. random architectures for a wide range of mutation rates and for different population sizes	
population size	150; 1,500; 15,000
crossover interference	Active
mating type	active and linked to the centromere of an (virtual) chromosome; the centromere of the simulated chromosome is located at position 333, which is equivalent to the centromere-position of chromosome IX.
recombination rate	*S. cerevisiae* chromosome IX rate
inbreeding percentage	0-100% in 25% steps
mutation rate R	10^{-4}, $3x10^{-4}$, 10^{-3}, $3x10^{-3}$, 10^{-2}, $3x10^{-2}$, 10^{-1}, $3x10^{-1}$, 1
simulation time	max. 40,000 generations; the simulation stopped as soon as one species became extinct.
total statistics	50 experiments per mutation rate and inbreeding fraction
chromosome architecture	*S. cerevisiae* chromosome IX building blocks (35 essential genes, 172 non-essential genes, 58 recombination hotspots, 148 recombination coldspots)<br>Note: random chromosomes contained the same number of elements as the yeast chromosome, but using a random distribution that was calculated independently for each simulation run.

Experimental settings for **Figure 4D**: Results of survival competition experiments for the sixteen yeast chromosomes versus randomly generated chromosome architectures	
population size	150; 1,500; 15,000
crossover interference	Active
mating type	active on external chromosome; centromere-linked locations:<br>135 (chr. I), 215 (chr. II), 115 (chr. III), 489 (chr. IV), 147 (chr. V),<br>123 (chr. VI), 511 (chr. VII), 91 (chr. VIII), 321 (chr. IX), 427 (chr. X),<br>447 (chr. XI), 127 (chr. XII), 277 (chr. XIII), 661 (chr. XIV),<br>319 (chr. XV), 551 (chr. XVI), derived from Mancera *et al*. (2008) [1]
recombination rate	average experimental rate for *S. cerevisiae*
inbreeding percentage	0-100% in 25% steps
mutation rate R	10^{-2}, 10^{-1}, 1
simulation time	max. 40,000 generations; the simulation stopped as soon as one species became extinct.
total statistics	35 experiments per mutation rate and inbreeding fraction
chromosome architecture	*S. cerevisiae* chromosomes were digitized using information about the distribution of approximately 4,000 crossover in 50 meiosis [1]. Essential gene locations were taken from www.yeastgenome.org.<br>Note: random chromosomes contained the same number of elements as the yeast chromosome, but using a random distribution that was calculated independently for each simulation run.

Experimental settings for **Figure 5A**: Mutational robustness R_{max} of a clustered chromosome (seven clusters) as compared to random chromosomes with the same genetic building blocks	
population size	200
crossover interference	Active
mating type	absent or active on the same chromosome
recombination rate	*S. cerevisiae* chromosome IX
inbreeding percentage	0-100% in 1% steps
mutational robustness R_{max} precision (step size)	0.02<br>Note: the scored R_{max} values denote the smallest values *R*, at which a population (specified by either chromosome IX or a randomly generated genome, and a particular inbreeding ration) became extinct within the maximally allowed number of generations.
simulation time	5,000 generations
total statistics	3 experiments per inbreeding fraction and subset (clustered/unclustered)
chromosome architecture	100 essential genes, 400 non-essential genes, 166 recombination hotspots, 333 recombination coldspots

Experimental settings for **Figure 5B**: Mutational robustness R_{max} of chromosome architectures with different levels of essential gene clustering and in populations of different sizes	
population size	50; 100; 200; 400; 1,600
crossover interference	Active
mating type	Absent
recombination rate	*S. cerevisiae* chromosome IX
inbreeding percentage	0-100% in 10% steps (figure shows averaged results)
mutational robustness R_{max} precision (step size)	0.02<br>Note: the scored R_{max} values denote the smallest values *R*, at which a population (specified by either chromosome IX or a randomly generated genome, and a particular inbreeding ration) became extinct within the maximally allowed number of generations.
simulation time	1,000 generations
total statistics	11 experiments per data point (with 11 different inbreeding fractions, see above)
chromosome architecture	100 essential genes, 400 non-essential genes, 166 recombination hotspots, 333 recombination coldspots

Experimental settings for **Figure 5C**: Survival competition of CF1+5 and CF1+R architectures (mating type in essential gene cluster, remaining essential genes in 5 clusters or random arrangement)	
population size	1,000
crossover interference	Active
mating type	active on external chromosome, location inside the essential gene cluster (one sixth of the total number of essential genes)
recombination rate	*S. cerevisiae* chromosome IX rate
inbreeding percentage	0-100% in 5% steps
mutation rate *R*	0.0-2.8 in 0.07 steps
simulation time	max. 10,000 generations, the simulation stopped as soon as one species became extinct.
total statistics	10 experiments per grid point
chromosome architecture	*S. cerevisiae* chromosome IX building blocks (35 essential genes, 172 non-essential genes, 58 recombination hotspots, 148 recombination coldspots)

Experimental settings for **Figure 6A**: Survival competition of super cluster architecture vs. random chromosomes	
population size	2,000
crossover interference	Active
mating type	active on the same chromosome (at position 41 or 333 respectively)
recombination rate	*S. cerevisiae* chromosome IX rate
inbreeding percentage	0-100% in 5% steps
mutation rate *R*	0.0-2.8 in 0.07 steps
simulation time	max. 10,000 generations, the simulation stopped as soon as one species became extinct.
total statistics	5 experiments per grid point
chromosome architecture	*S. cerevisiae* chromosome IX building blocks (35 essential genes, 172 non-essential genes, 58 recombination hotspots, 148 recombination coldspots)

Experimental settings for **Figure 6B/C**: Survival competition of super cluster architecture vs. random architectures for a wide range of mutation rates and for different population sizes	
population size	150; 1,500; 15,000
crossover interference	Active
mating type	active on same (b) or external chromosome (c), located inside super cluster
recombination rate	*S. cerevisiae* chromosome IX rate
inbreeding percentage	0-100% in 25% steps
mutation rate *R*	10^{-4}, $3x10^{-4}$, 10^{-3}, $3x10^{-3}$, 10^{-2}, $3x10^{-2}$, 10^{-1}, $3x10^{-1}$, 1
simulation time	max. 40,000 generations, the simulation stopped as soon as one species became extinct.
total statistics	50 experiments per mutation rate and inbreeding fraction
chromosome architecture	*S. cerevisiae* chromosome IX building blocks (35 essential genes, 172 non-essential genes, 58 recombination hotspots, 148 recombination coldspots)

Experimental settings for **Figure 7A/B**: Maintenance of essential gene clustering	
population size	2,000
crossover interference	Active
mating type	absent or active on the same chromosome
recombination rate	*S. cerevisiae* chromosome IX rate
inbreeding percentage	100%
mutation rate *R*	0.0-2.4 in 0.2 steps
rearrangement rate	$5x10^{-5}$ and 10^{-4} per genetic building block and mitosis
simulation time	150,000 generations
total statistics	6 experiments per grid point in +*MAT* subset (each 3 experiments with *MAT* initially inside/outside of essential gene), 3 experiments per grid point in -*MAT* series
chromosome architecture	*S. cerevisiae* chromosome IX building blocks (35 essential genes, 172 non-essential genes, 58 recombination hotspots, 148 recombination coldspots)

Experimental settings for **Figure 7C/D**: Evolution of essential gene clustering in small genomes	
population size	100
crossover interference	Active
mating type	absent or active on the same chromosome
recombination rate	minimum (1 hotspot) and maximum (all hotspots = 4 hotspots)
inbreeding percentage	100%
mutation rate *R*	2.0
rearrangement rate	10^{-5} and $3x10^{-5}$ per genetic building block and mitosis
simulation time	100,000 generations
total statistics	1,000 experiments per mating type configuration and recombination rate
chromosome architecture	5 essential genes, 5 non-essential genes, 4 recombination hotspots, 5 recombination coldspots

Experimental settings for **Figure 8A**: Evolution of essential gene clustering in large yeast chromosome IX-like genomes under mixed inbreeding/outbreeding conditions	
population size	4,000
crossover interference	Active
mating type	absent or active on the same chromosome
recombination rate	*S. cerevisiae* chromosome IX rate
inbreeding percentage	50%
mutation rate *R*	0.1; 1.0
rearrangement rate	$3x10^{-5}$ per genetic building block and mitosis
simulation time	100,000 generations
total statistics	830 experiments for *R*=1.0/+*MAT*<br>826 experiments for *R*=0.1/+*MAT*<br>839 experiments for *R*=1.0/-*MAT*
chromosome architecture	initial arrangement: maximally unclustered architecture (clustering score 59) consisting of *S. cerevisiae* chromosome IX building blocks (35 essential genes, 172 non-essential genes, 58 recombination hotspots, 148 recombination coldspots)

Experimental settings for **Figure 8B/C**: Survival competition of evolution products from the experiments shown in 5E (red and green subsets) vs. random architectures and *S. cerevisiae* chromosome IX	
population size	2,000
crossover interference	Active
mating type	active on the same chromosome, location at the respective evolved position in the evolution products and at position 333 for *S. cerevisiae* chromosome IX
recombination rate	*S. cerevisiae* chromosome IX rate
inbreeding percentage	0-100% in 25% steps
mutation rate *R*	10^{-3}, 10^{-2}, 10^{-1}, 1
simulation time	max. 10,000 generations, the simulation stopped as soon as one species became extinct.
total statistics	competitions were performed for all evolution products<br>(*n* = 830 populations for the red subset and *n* = 826 populations for the green subset).
chromosome architecture	evolved architectures (products of previous experiment), *S. cerevisiae* chromosome IX and random architectures with *S. cerevisiae* chromosome IX building blocks (35 essential genes, 172 non-essential genes, 58 recombination hotspots, 148 recombination coldspots)

Experimental settings for **Figure 9**: Survival competition of *S. cerevisiae* chromosome IX vs. random architectures with and without mating type switching	
population size	2,000
crossover interference	Active
mating type	active and linked to the centromere of a (virtual) chromosome; the centromere of the simulated chromosome is located at position 333, which is equivalent to the centromere-position of chromosome IX.
recombination rate	*S. cerevisiae* chromosome IX rate
inbreeding percentage	0-100% in 5% (A) or 25% (B) steps, 0/10% mating type switching
mutation rate R	0.0-2.8 in 0.07 steps (A), $10^{-3}/10^{-2}/10^{-1}/1$ (B)
simulation time	max. 10,000 generations; the simulation stopped as soon as one species became extinct.
total statistics	10 (A) or 100 (B) experiments per grid point
chromosome architecture	*S. cerevisiae* chromosome IX building blocks (35 essential genes, 172 non-essential genes, 58 recombination hotspots, 148 recombination coldspots) Note: random chromosomes contained the same number of elements as the yeast chromosome, but using a random distribution that was calculated independently for each simulation run.

Experimental settings for **Figure 10A**: Mutational robustness at different recombination rates	
population size	100
crossover interference	Active
mating type	active on the same chromosome
recombination rate	between 1 and 58 crossovers per chromosome and meiosis
inbreeding percentage	0-100% in 20% steps (figure shows averaged results)
mutational robustness R_{max} precision (step size)	0.01 Note: the scored R_{max} values denote the smallest values R, at which a population became extinct within the maximally allowed number of generations.
simulation time	2,000 generations
total statistics	6 experiments per data point (with 6 different inbreeding fractions, see above)
chromosome architecture	random architecture; 50/100/150/200/300 essential genes (20%), 200/400/600/800/1,200 non-essential genes, 84/166/250/336/498 recombination hotspots, 165/333/499/663/1,001 recombination coldspots

Experimental settings for **Figure 10B**: Survival competition of two *S. cerevisiae* chromosome IX populations with different recombination rates	
population size	1,000
crossover interference	Active
mating type	active on external chromosome, centromere-linked location: 333
recombination rate	first population: 1 crossover per chromosome and meiosis (min. rate), 0.5x/1.0x/2.0x/4.0x *S. cerevisiae* chromosome IX rate, all hotspots active at each meiosis (max. rate)<br>second population: *S. cerevisiae* chromosome IX rate
inbreeding percentage	0-100% in 5% steps
mutation rate *R*	0.0-2.8 in 0.07 steps
simulation time	10,000 generations
total statistics	5 experiments per grid point
chromosome architecture	*S. cerevisiae* chromosome IX building blocks (35 essential genes, 172 non-essential genes, 58 recombination hotspots, 148 recombination coldspots)

Experimental settings for **Figure 10C**: Survival competition of *S. cerevisiae* chromosome IX vs. random chromosomes for different recombination rates	
population size	1,000
crossover interference	Active
mating type	active on external chromosome, centromere-linked location: 333
recombination rate	1 crossover per chromosome and meiosis (min. rate),<br>0.5x/1.0x/2.0x/4.0x *S. cerevisiae* chromosome IX rate,<br>all 58 hotspots active in each meiosis (max. rate)
inbreeding percentage	0-100% in 5% steps
mutation rate *R*	0.0-2.8 in 0.07 steps
simulation time	10,000 generations
total statistics	10 experiments per grid point
chromosome architecture	*S. cerevisiae* chromosome IX building blocks (35 essential genes, 172 non-essential genes, 58 recombination hotspots, 148 recombination coldspots)

Experimental settings for **Supplementary Figure 2A-C/E/F**: Mutational robustness R_{max} of *S. cerevisiae* chromosome IX and random architectures for different mutation and mating type switching settings	
population size	200
crossover interference	Active
mating type	active and linked to the centromere of a (virtual) chromosome; the centromere of the simulated chromosome is located at position 333, which is equivalent to the centromere-position of chromosome IX.
recombination rate	2.63 crossovers per chromosome and meiosis
inbreeding percentage	0-100% in 20% steps (figure shows averaged results)
mating type switching	0% (A-C), 10% (E), 50% (F)
mutational robustness R_{max} precision (step size)	0.007 (A/B), 0.014 (C/E/F)<br>Note: the scored R_{max} values denote the smallest values *R*, at which a population (specified by either chromosome IX or a randomly generated genome, and a particular inbreeding ration) became extinct within the maximally allowed number of generations.
mutation application	mitosis only (A), meiosis only (B), mitosis and meiosis (C/E/F)
simulation time	2,000 generations
total statistics	3,000 (A-C) or 1,000 (E/F) experiments per subset (chromosome IX and random)
chromosome architecture	*S. cerevisiae* chromosome IX building blocks (35 essential genes, 172 non-essential genes, 58 recombination hotspots, 148 recombination coldspots)<br>Note: random chromosomes contained the same number of elements as the yeast chromosome, but using a random distribution that was calculated independently for each simulation run.

Experimental settings for **Supplementary Figure 2D**: Survival competition of mitotic mutators vs. meiotic mutators for *S. cerevisiae* chromosome VI	
population size	200
crossover interference	Active
mating type	active and linked to the centromere of an (virtual) chromosome; the centromere of the simulated chromosome is located at position 123, which is equivalent to the centromere-position of chromosome VI.
recombination rate	*S. cerevisiae* chromosome VI rate
inbreeding percentage	50%
mutation rate *R*	0.1
mutation application	mitosis only (population #1) vs. meiosis only (population #2)
simulation time	max. 2,000 generations; the simulation stopped as soon as one species became extinct.
total statistics	5,000 experiments
chromosome architecture	*S. cerevisiae* chromosome VI (27 essential genes, 94 non-essential genes, 57 recombination hotspots, 63 recombination coldspots)

Experimental settings for **Supplementary Figure 3**: The effect of essential gene clustering on mutational robustness at different inbreeding rates	
population size	200
crossover interference	Active
mating type	absent (-*MAT*) or active inside one of the EG clusters (+*MAT*)
recombination rate	7.79 crossovers per chromosome and meiosis
inbreeding percentage	0-100% in 10% steps
mutational robustness R_{max} precision (step size)	0.02<br>Note: the scored R_{max} values denote the smallest values R, at which a population became extinct within the maximally allowed number of generations.
simulation time	1,000 generations
total statistics	1 experiment per grid point
chromosome architecture	100 essential genes, 400 non-essential genes, 166 recombination hotspots, 333 recombination coldspots; essential genes were arranged in n clusters, with n = 1, 2, 3, 4, 5, 6, 7, 8, 9, 10, 12, 14, 16, 18, 20, 30, 50, 100.

Experimental settings for **Supplementary Figure 4**: Evolution of essential gene clustering in small model chromosomes	
population size	200
crossover interference	Active
mating type	absent (-*MAT*) or active (+*MAT*)
recombination rate	recombination occurring at all four hotspots in each meiosis
inbreeding percentage	100%
mutation rate R	0.5, 1.0, 1.5, 2.0
simulation time	100,000 generations
total statistics	10 experiments per grid point
chromosome architecture	5 essential genes, 5 non-essential genes, 4 recombination hotspots, 5 recombination coldspots; the initial architecture was maximally unclustered (clustering score ν = 5).

Experimental settings for **Supplementary Figure 5A**: Evolution of essential gene clustering in large yeast chromosome X-like genomes under mixed inbreeding/outbreeding conditions	
population size	2,000
crossover interference	Active
mating type	absent or active on the same chromosome
recombination rate	*S. cerevisiae* chromosome X rate
inbreeding percentage	50%
mutation rate *R*	0.1; 1.0
rearrangement rate	10^{-5} per genetic building block and mitosis
simulation time	200,000 generations
total statistics	495 experiments for *R*=1.0/+*MAT*<br>500 experiments for *R*=0.1/+*MAT*<br>500 experiments for *R*=1.0/-*MAT*
chromosome architecture	initial arrangement: maximally unclustered architecture (clustering score 131) consisting of *S. cerevisiae* chromosome X building blocks (72 essential genes, 285 non-essential genes, 130 recombination hotspots, 226 recombination coldspots)

Experimental settings for **Supplementary Figure 5B/C**: Survival competition of evolution products from the experiments shown in 5A (all subsets) vs. random architectures and vs. *S. cerevisiae* chromosome X	
population size	2,000
crossover interference	Active
mating type	active on the same chromosome, location at the respective evolved position in the evolution products and at position 427 for *S. cerevisiae* chromosome X
recombination rate	*S. cerevisiae* chromosome X rate
inbreeding percentage	0-100% in 25% steps
mutation rate *R*	10^{-3}, 10^{-2}, 10^{-1}, 1
simulation time	max. 10,000 generations, the simulation stopped as soon as one species became extinct.
total statistics	competitions were performed for n = 100 randomly chosen evolution products from each subset.
chromosome architecture	evolved architectures (products of previous experiment), *S. cerevisiae* chromosome X and random architectures with *S. cerevisiae* chromosome X building blocks (72 essential genes, 285 non-essential genes, 130 recombination hotspots, 226 recombination coldspots)

Experimental settings for **Supplementary Figure 6**: Survival competition of mating type switching vs. non-switching in *S. cerevisiae* chromosome VI with different levels of mutational pre-load	
population size	1,000
crossover interference	Active
mating type	active and linked to the centromere of a (virtual) chromosome; the centromere of the simulated chromosome is located at position 123, which is equivalent to the centromere-position of chromosome VI.
recombination rate	*S. cerevisiae* chromosome VI rate
inbreeding percentage	0-100% in 25% (B) steps, 0% vs. 2/10/25% mating type switching
mutation rate *R*	10^{-3}, 10^{-2}, 10^{-1}, 1
mutation pre-load	0/2/4/6/8/10 deactivated EGs (distributed on both homologues)
simulation time	max. 5,000 generations; the simulation stopped as soon as one species became extinct.
total statistics	100 experiments per grid point on each matrix
chromosome architecture	*S. cerevisiae* chromosome VI (27 essential genes, 94 non-essential genes, 57 recombination hotspots, 63 recombination coldspots)

Experimental settings for **Supplementary Figure 8**: Survival competition experiments at different recombination rates
all parameter settings as in Figure 10B/C in Main Text

F) References

1. Mancera E, Bourgon R, Brozzi A, Huber W, Steinmetz LM (2008) High-resolution mapping of meiotic crossovers and non-crossovers in yeast. Nature 454: 479-U471.
2. Housworth EA, Stahl FW (2003) Crossover interference in humans. American Journal of Human Genetics 73: 188-197.
3. Stahl FW, Foss HM, Young LS, Borts RH, Abdullah MFF, *et al*. (2004) Does crossover interference count in *Saccharomyces cerevisiae*? Genetics 168: 35-48.
4. Knop M (2006) Evolution of the hemiascomycete yeasts: on life styles and the importance of inbreeding. Bioessays 28: 696-708.
5. Gerton JL, DeRisi J, Shroff R, Lichten M, Brown PO, *et al*. (2000) Inaugural article: global mapping of meiotic recombination hotspots and coldspots in the yeast *Saccharomyces cerevisiae*. Proc Natl Acad Sci U S A 97: 11383-11390.
6. Sherman F, Wakem P (1991) Mapping Yeast Genes. Meth Enzymol 194: 38-57.
7. Taxis C, Keller P, Kavagiou Z, Jensen LJ, Colombelli J, *et al*. (2005) Spore number control and breeding in *Saccharomyces cerevisiae*: a key role for a self-organizing system. J Cell Biol 171: 627-640.
8. Batada NN, Hurst LD (2007) Evolution of chromosome organization driven by selection for reduced gene expression noise. Nat Genet 39: 945-949.
9. Alani E, Cao L, Kleckner N (1987) A method for gene disruption that allows repeated use of URA3 selection in the construction of multiply disrupted yeast strains. Genetics 116: 541-545.
10. Janke C, Magiera MM, Rathfelder N, Taxis C, Reber S, *et al*. (2004) A versatile toolbox for PCR-based tagging of yeast genes: new fluorescent proteins, more markers and promoter substitution cassettes. Yeast 21: 947-962.
11. Coluccio A, Neiman AM (2004) Interspore bridges: a new feature of the *Saccharomyces cerevisiae* spore wall. Microbiology 150: 3189-3196.
12. Pal C, Hurst LD (2003) Evidence for co-evolution of gene order and recombination rate. Nat Genet 33: 392-395.
